# Endpoint-specific mortality associated with malignancy among adults with sepsis or septic shock: a systematic review and meta-analysis

**DOI:** 10.1080/07853890.2026.2752656

**Published:** 2026-10-30

**Authors:** Huei Chung, Hung-Chang Chou, Tsung-Yu Tsai, Ming-Chu Chung

**Affiliations:** a Department of Pharmacy, Taoyuan General Hospital, Ministry of Health and Welfare, Taoyuan, Taiwan; b Department of Pharmacy and Master Program, Tajen University, Pingtung, Taiwan; c Department of Epidemiology, Gillings School of Global Public Health, University of North Carolina at Chapel Hill, NC, USA; d Department of Psychiatry, Taoyuan Armed Forces General Hospital, Taoyuan, Taiwan

**Keywords:** Sepsis, septic shock, cancer, mortality, critical care

## Abstract

**Background:**

Sepsis is a major global health challenge and increasingly affects patients with complex conditions such as malignancy. Prior studies suggest higher mortality in this population, but interpretation is limited by aggregation of heterogeneous endpoints. We conducted a systematic review and meta-analysis to clarify endpoint-specific prognostic associations between malignancy and mortality in adults with sepsis or septic shock.

**Methods:**

This study was registered in PROSPERO (CRD420251141216). PubMed, Embase, Web of Science, Cochrane Library and Epistemonikos were searched through 16 September 2025, for studies comparing all-cause mortality between sepsis patients with and without malignancy. Mortality endpoints were grouped as ICU, in-hospital, early, intermediate and extended follow-up mortality. Crude odds ratios (ORs) were estimated using random-effects meta-analysis. Subgroup, meta-regression and sensitivity analyses were performed to explore heterogeneity and assess consistency.

**Results:**

Among 105 eligible studies comprising 20,089,138 participants, 85 studies contributing 101 endpoint estimates were included in the primary synthesis. Pooled estimates were elevated across endpoints: ICU (OR 1.21, 95% CI 0.94–1.56), in-hospital (OR 1.90, 95% CI 1.69–2.13), early (OR 1.81, 95% CI 1.59–2.06), intermediate (OR 2.28, 95% CI 1.72–3.03) and extended follow-up mortality (OR 2.41, 95% CI 1.70–3.42). Associations were sustained for haematologic malignancy, concentrated in in-hospital and early mortality for metastatic disease and more modest for solid tumours. Sensitivity analyses were directionally consistent, although risk ratio estimates were generally smaller than ORs. Exploratory meta-regression suggested that severity-related covariates may contribute to heterogeneity.

**Conclusions:**

Malignancy was associated with higher mortality among adults with sepsis or septic shock, with variation across mortality endpoints and clinical contexts. These findings support endpoint-specific prognostic assessment and highlight the need for future studies to incorporate more granular cancer characteristics. Because the primary estimates were unadjusted, results represent overall prognostic associations rather than independent causal effects.

## KEY MESSAGES

1. Malignancy was consistently associated with higher mortality among adults with sepsis or septic shock, with pooled ORs ranging from 1.21 for ICU mortality to 2.41 for extended follow-up mortality.
2. Subtype analyses suggested a more sustained pattern for haematologic malignancy than for solid tumours across available endpoints.
3. Endpoint-specific assessment may improve prognostic interpretation by distinguishing mortality associations according to the outcome timeframe assessed.

## Background

Sepsis remains a major global health challenge, with the Global Burden of Disease 2021 [1] estimating 166 million incident cases and 21.4 million deaths – nearly one third of all global deaths. Although mortality from infectious causes declined between 1990 and 2019 [2], the burden rose again during 2020–2021, driven by COVID-19 and ageing populations with chronic comorbidities. Increasingly, sepsis arises at the intersection of infection and chronic non-communicable diseases. As survival among patients with chronic diseases improves, sepsis is now more often encountered in populations with complex baseline health conditions, including malignancy [3]. This convergence of infection, ageing, multimorbidity and cancer survivorship highlights the need to understand how baseline host factors shape both acute and post-acute sepsis outcomes [4].

Malignancy contributes to sepsis prognosis through tumour-related immune dysregulation, treatment-related immunosuppression, reduced physiological reserve and disruption of anticancer therapy [5,6]. The clinical relevance of this population is underscored by a U.S. nationwide analysis of 1.1 million sepsis hospitalizations, in which 21.2% were cancer-related [7]. These mechanisms are particularly relevant in older or multimorbid patients, where sepsis may precipitate delayed recovery and reduced resilience to subsequent treatment [8]. These factors may shape prognosis differently across acute, inpatient and longer-term sepsis outcomes [9–11].

Previous meta-analyses [12–15] consistently reported poor outcomes among patients with cancer and sepsis, including intensive care unit (ICU) mortality of approximately 44–48% and hospital mortality of 54–62%. These studies also found higher mortality associations compared with non-cancer patients. However, prior analyses often combined heterogeneous mortality endpoints into a single estimate, potentially masking endpoint-specific prognostic patterns. This distinction is clinically relevant because mortality endpoints differ in the clinical contexts and risk periods they represent. To address this gap, we conducted a systematic review and meta-analysis to evaluate the crude association between malignancy and mortality among adults with sepsis or septic shock across distinct mortality endpoint categories. We also examined variation by cancer-related characteristics, clinical context and severity-related study factors.

## Methods

This review was registered in PROSPERO (CRD420251141216), where the protocol is accessible. This review was reported in accordance with the Preferred Reporting Items for Systematic Reviews and Meta-Analyses (PRISMA) 2020 guidelines [16]. The completed checklist is in Supplementary Material 1, and protocol amendments with deviations, rationale and timing are in Supplementary Material 2.

### Search strategy

We searched PubMed, Embase, Web of Science, the Cochrane Library and Epistemonikos from inception to 16 September 2025, using terms related to sepsis, septic shock, malignancy, cancer and mortality. Reference lists of relevant reviews and eligible studies were also screened. The complete electronic search strategies for all databases are provided in Supplementary Material 3.

### Eligibility criteria

Eligible studies enrolled hospitalized patients aged ≥16 years with sepsis, severe sepsis, or septic shock, as defined by the original investigators and compared mortality between patients with malignancy and non-cancer comparators. Observational cohort, case-control, cross-sectional studies and randomized clinical trials (RCTs) were eligible. Studies limited to paediatric, pregnant, HIV-associated, or COVID-19–related populations were excluded. General adult sepsis studies remained eligible if HIV-positive participants comprised <5% of the cohort or if analyses excluding HIV-positive participants were available. Conference abstracts, books, case reports, case series, qualitative studies, non-English articles and studies without a relevant comparator group were excluded. For overlapping cohorts, the most appropriate study was retained; other reports were excluded or, when relevant, incorporated into appropriate endpoint or subgroup analyses.

### Study selection

Records were deduplicated using EndNote X20. After duplicate removal, title/abstract screening and full-text eligibility assessment were performed independently and in duplicate. HC screened all records, while HCC, TYT and MCC each independently screened one-third; thus, each record and full-text report was assessed by two reviewers. Each reviewer completed the assessment independently before screening decisions were compared and was unaware of the paired reviewer’s decision during screening. Disagreements were resolved by consensus and, when necessary, by third-reviewer arbitration.

### Data extraction and quality assessment

Data extraction used standardized, piloted tables. MCC extracted data, independently verified by HC. Variables included study characteristics, population features, mortality outcomes with time points and interventions in RCTs. Cancer type was recorded as reported; unclear definitions were categorized as ‘cancer’. Risk of bias was assessed using the Newcastle-Ottawa Scale (NOS) [17] for cohort/case-control studies, an adapted NOS [18] for cross-sectional studies and Cochrane risk-of-bias tool-2 (RoB-2) [19] for RCTs. Given substantial heterogeneity, no formal Grading of Recommendations Assessment, Development and Evaluation (GRADE) assessment was performed; interpretation was supplemented by subgroup, sensitivity and meta-regression analyses.

### Outcome and effect measures

The outcome was defined as all-cause mortality as most studies did not distinguish infection-attributable mortality. The main analysis prioritized crude prognostic associations because covariate adjustment varied across studies, whereas event data were available in more than 80% of included studies. When both adjusted and unadjusted results were reported, unadjusted values were used for the primary analysis, and adjusted results were extracted separately for sensitivity analyses. Mortality endpoints were categorized before synthesis into ICU, in-hospital, early (28/30-day), intermediate (60/90-day) and extended follow-up (≥180-day) mortality. Endpoints shorter than 28 days were identified but not pooled because they were sparse. Extended follow-up pooled 180-day and longer outcomes due to limited data.

Odds ratios (ORs) were the primary effect measure because they were most consistently reported and maximized study inclusion. Crude ORs were calculated from event data or extracted directly when reported. Studies without sufficient data for crude OR calculation were excluded from the primary analysis but considered in sensitivity analyses. ORs were interpreted strictly as relative odds rather than relative risks (RRs), given the high mortality frequency. Sensitivity analyses used alternative available effect measures, including RR-based estimates derived from event data or directly reported RRs, hazard ratios (HRs) and adjusted ORs, when sufficient data were available.

### Data analysis

Descriptive statistics were performed in SPSS (v31). Meta-analyses were conducted in R (v4.5.3) within RStudio (version 2026.01.01 403). Estimates were synthesized on the log scale using random-effects models and back-transformed for interpretation. Forest plots displayed pooled estimates, study weights and 95% confidence intervals (CIs). One estimate per study was retained within each endpoint category to avoid double counting and analyses were restricted to categories with at least three studies. Between-study heterogeneity was assessed using *I*^2^ (%) [20]. Reporting bias was evaluated using Doi plots and the LFK index, supplemented by funnel plots and Egger’s regression when ≥10 studies were available [21,22]. LFK values between −1 and 1 indicated no asymmetry, values from −2 to −1 or 1 to 2 indicated minor asymmetry and values beyond ±2 indicated major asymmetry.

### Subgroup, sensitivity and meta-regression analyses

Subgroup analyses were conducted when sufficient data were available, based on the planned cancer subtype analysis and clinically relevant characteristics reported by eligible studies. An additional post hoc subgroup analysis compared studies using Sepsis-1/2 definitions with those using Sepsis-3, with between-group differences assessed using tests for subgroup interaction. For overlapping cancer subgroups with a shared non-cancer control, data were combined for the primary analysis. Robustness was examined through sensitivity analyses addressing effect-measure choices, exclusion of International Classification of Diseases (ICD)-based studies, exclusion of very large studies, restriction to studies in which malignancy was the primary exposure, leave-one-out analyses and robust variance estimation (RVE). Post hoc exploratory meta-regression was limited to 28/30-day or in-hospital mortality and assessed study-level (analytic context, enrolment setting, geographic region, sepsis definition and study year), severity-related (ICU admission, mechanical ventilation, renal replacement therapy, septic shock and SOFA score) and population-level covariates (age, female proportion and cancer-related proportions). Univariable models were followed by parsimonious multivariable models, adding severity-related covariates sequentially. Explained heterogeneity was quantified by R^2^, with moderator significance tested by omnibus statistics. Cumulative meta-analysis by publication year was used to assess the statistical stability of cumulative evidence.

## Results

### Study selection and characteristics

The study selection process is shown in Figure 1. The database search identified 67,436 records, with an additional 903 records retrieved through manual reference screening. After duplicate removal, 49,999 records were screened and 830 articles underwent full-text review, resulting in 105 studies meeting the inclusion criteria, with detailed study-level data in Supplementary Material 4, which also includes 12 studies with shared cohorts.

**Figure 1.**
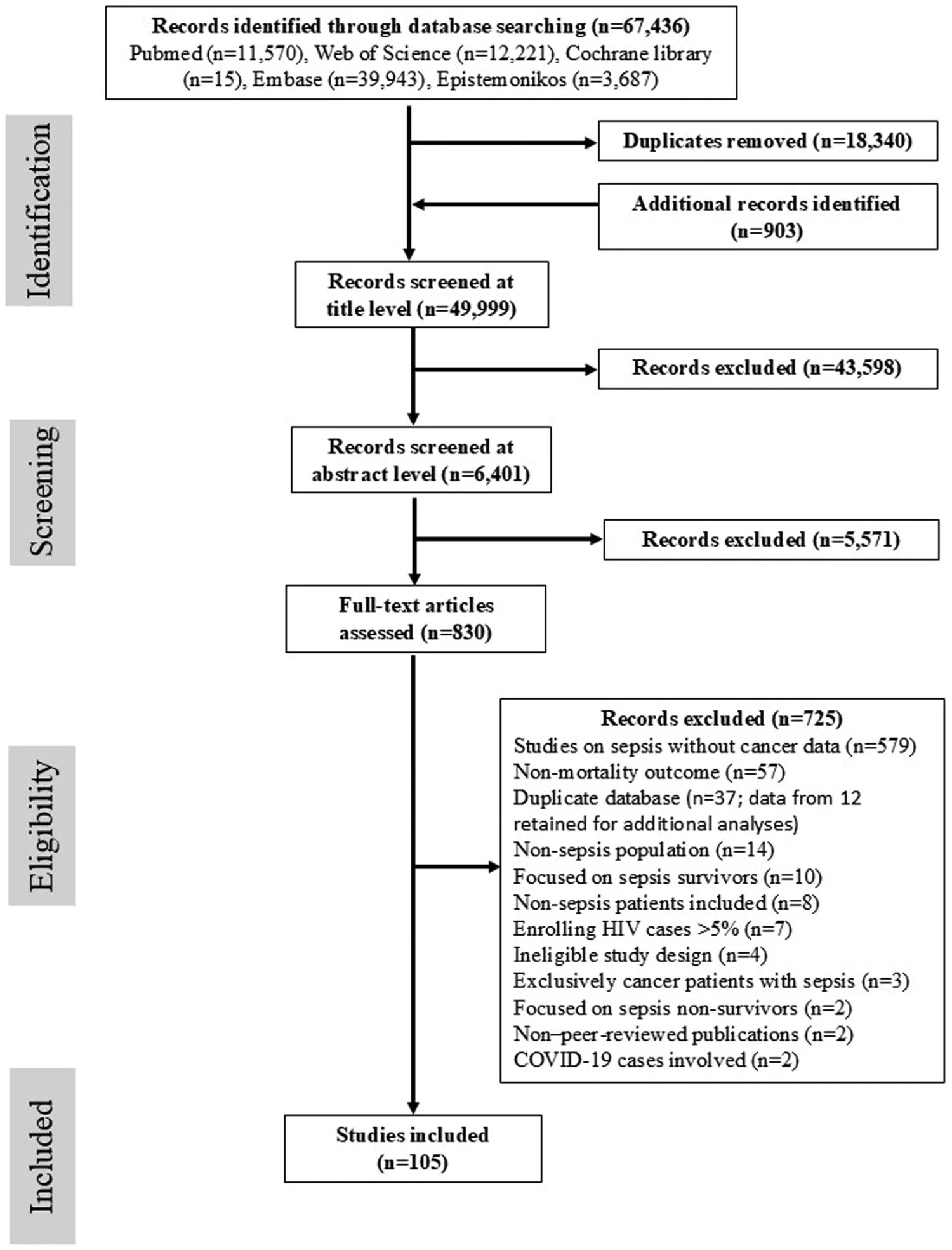
Study selection flow diagram.

Summary characteristics are presented in Table 1. The included studies spanned 1976–2025, with most conducted after 2000 and were predominantly based in Asia and Europe. Sample sizes varied considerably: the majority (62.9%) enrolled 101–1,000 participants, while a small proportion (2.9%) included >100,000 patients. In total, 20,089,138 participants were analysed, with a pooled mean age of 65.5 years (SD 17.4). Sepsis definitions varied, with 17 studies (16.2%) using Sepsis-1, 32 (30.5%) Sepsis-2, 34 (32.4%) Sepsis-3, 12 (11.4%) ICD-based administrative definitions and 9 (8.6%) other criteria. Enrolment settings were predominantly ICU (56.2%), followed by inpatient (24.8%) and emergency department (ED; 15.2%), with the remaining 3.8% from mixed settings.

**Table 1.** Summary characteristics of included studies.

Characteristic	No. (%)	Characteristic	No. (%)
Age (mean)^a^		Study design	
40–49	2 (1.9)	Cohort	99 (94.3)
50–59	5 (4.8)	Randomized controlled trial	4 (3.8)
60–69	70 (66.7)	Cross-sectional	2 (1.9)
≥70	26 (24.8)	Analytic context	
Not reported	2 (1.9)	Cancer as a comorbidity	90 (85.7)
SOFA (mean)		Cancer as primary exposure	15 (14.3)
0–6	13 (12.4)	Sepsis definition	
7–9	33 (31.4)	Sepsis-1	17 (16.2)
>9	7 (6.7)	Sepsis-2	32 (30.5)
Not reported	52 (49.5)	Sepsis-3	34 (32.4)
Enrolment setting		ICD-based	12 (11.4)
ICU-restricted	59 (56.2)	Other definitions	9 (8.6)
General inpatient	26 (24.8)	Not reported	1 (1.0)
ED-based	16 (15.2)	Study scope	
Mixed	4 (3.8)	Single-centre	71 (67.6)
Study year^b^		Multicentre or regional	19 (18.1)
<2002	4 (3.8)	Nationwide	5 (4.8)
2002–2014	52 (49.5)	Population-based	4 (3.8)
2015–2021	45 (42.9)	Multinational	5 (4.8)
>2021	2 (1.9)	Not reported	1 (1.0)
Not reported	2 (1.9)	Geographic regions	
Study sample size		Asia	51 (48.6)
≤100	11 (10.5)	Europe	32 (30.5)
101–1000	66 (62.9)	North America^c^	14 (13.3)
1001–10,000	18 (17.1)	South America	6 (5.7)
10,001–100,000	7 (6.7)	Oceania	1 (1.0)
>100,000	3 (2.9)	Global	1 (1.0)

*Note:* ED: emergency department; ICD: International Classification of Diseases; ICU: intensive care unit; SOFA: sequential organ failure assessment score.

^a^ Age stratification was based on mean age; if only median age was reported, the median was used instead.

^b^If the study year contained multiple years, the median year was used as the basis for temporal classification and analysis.

^c^Beck et al. [41] were classified as a North American study because most sites were conducted in the United States and Canada, with one additional site in Saudi Arabia.

Of the 105 studies, 99 (94.3%) were cohort designs, 4 (3.8%) RCTs and 2 (1.9%) cross-sectional studies. Of the four RCTs, two evaluated sepsis interventions: parenteral fish oil [23] and hypertonic saline/dextran [24]. The remaining two were post hoc analyses of RCTs: the Transfusion Requirements in Septic Shock (TRISS) trial [25] and the Prehospital Antibiotics Against Sepsis (PHANTASi) trial [26]. Fifteen studies [26–40] directly compared mortality between sepsis patients with and without cancer, whereas the remaining 90 studies, across different study designs, treated cancer as a comorbidity rather than the primary exposure.

### Risk of bias assessment

Detailed assessments are provided in Supplementary Material 5. Using RoB 2.0 criteria, the four RCTs were classified as having some concerns, mainly related to the selection or reporting of mortality outcomes. Two trials were post hoc analyses, and the other two reported mortality only as a secondary or exploratory endpoint. The adapted NOS for cross‑sectional studies indicated that one study was rated very good, while another was unsatisfactory due to methodological limitations. Overall study quality assessed with the NOS for cohort studies was acceptable, with 19 studies rated high, 75 moderate and 5 low. Most cohort studies lost points because of limited representativeness from single‑centre designs or incomplete definitions of cancer as a comorbidity in data collection.

### Mortality associations across stratified endpoints

Of 105 eligible studies, seven [40,42–47] reported HRs and three [48–50] reported only adjusted ORs as their only effect estimates; these studies were excluded from the primary meta-analysis. Three studies reported short-term endpoints that were not pooled because they were clinically heterogeneous: 72-hour [51], 3-day [30] and 14-day [29] mortality. Two [29,30] of these studies were retained for other eligible mortality endpoints. Ten studies provided subgroup-specific estimates and were incorporated into cancer subtype or disease-extent analyses. Endpoint handling was further standardized by including both available endpoints from one MIMIC-IV shared-cohort study [52] – 28-day and 90-day mortality – and retaining the 180-day endpoint, rather than the overlapping 1-year endpoint, from one study [31]. Furthermore, eight studies [26,28–32,35,38] contributed multiple endpoint estimates, yielding 15 additional endpoints. In total, 85 studies comprising 101 endpoint estimates were included in the primary synthesis.

Figure 2 presents the forest plots of endpoint-stratified analyses. ICU mortality was reported in 9 studies (*n* = 3,235), in-hospital mortality in 43 studies (*n* = 19,736,664), early mortality in 34 studies (*n* = 31,910), intermediate mortality in 11 studies (*n* = 47,625) and extended follow-up mortality in 4 studies (*n* = 1,881). Pooled unadjusted ORs were 1.21 (95% CI, 0.94–1.56; *I*^2^ = 50.6%; Figure 2(A)) for ICU mortality, 1.90 (95% CI, 1.69–2.13; *I*^2^ = 97.2%; Figure 2(B)) for in-hospital mortality, 1.81 (95% CI, 1.59–2.06; *I*^2^ = 43.4%; Figure 2(C)) for early mortality, 2.28 (95% CI, 1.72–3.03; *I*^2^ = 88.8%; Figure 2(D)) for intermediate mortality and 2.41 (95% CI, 1.70–3.42; *I*^2^ = 59.5%; Figure 2(E)) for extended follow-up mortality. Leave-one-out analyses confirmed the stability of pooled estimates, with no single study materially altering the primary association. Doi plots and funnel plots are presented in Supplementary Material 6. The LFK index suggested no asymmetry for ICU mortality (0.10) and early mortality (−0.67), minor asymmetry for intermediate mortality (1.08) and extended follow-up mortality (−1.65) and major asymmetry for in-hospital mortality (3.57). Egger’s regression did not indicate statistically significant small-study effects for in-hospital, early, or intermediate mortality endpoints (*p* = 0.09, 0.46 and 0.51, respectively).

Figure 2.Forest plots of mortality associations across stratified endpoints: (A) intensive care unit, (B) in-hospital, (C) early (28/30-day), (D) intermediate (60/90-day) and (E) extended follow-up mortality (≥180-day). OR: odds ratio.

**Figure:**
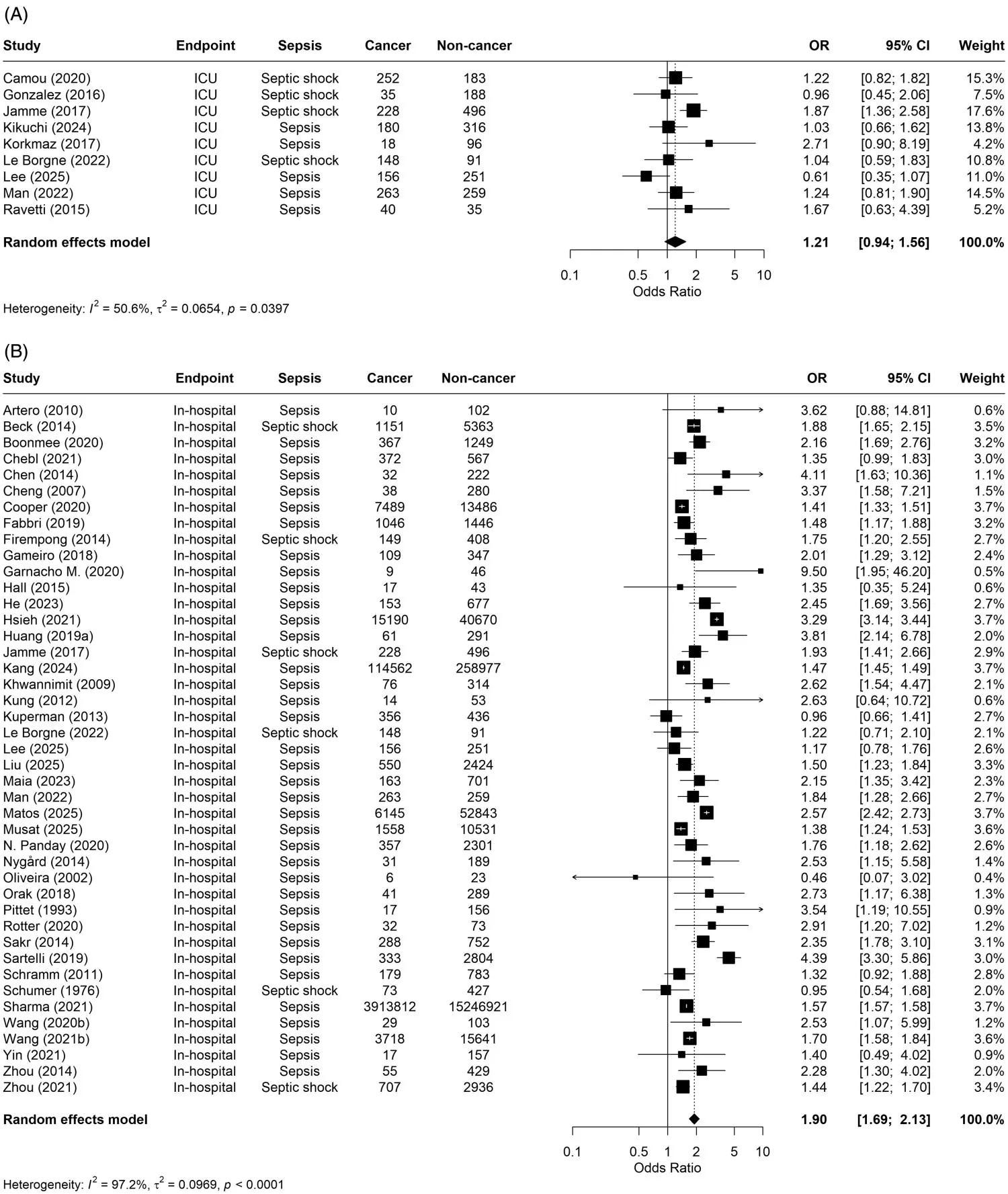

**Figure:**
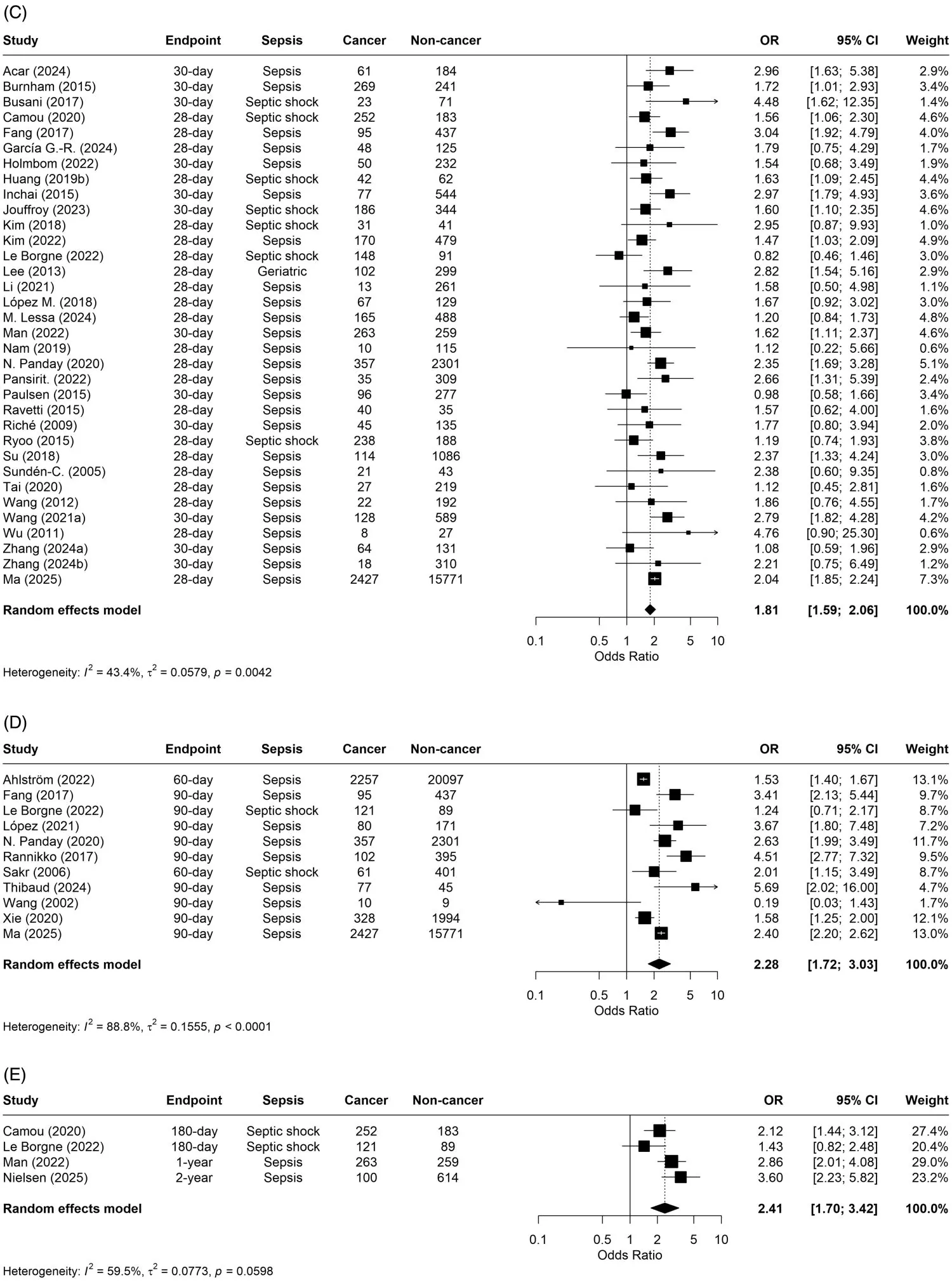

Absolute mortality was additionally summarized among studies reporting raw event counts (Supplementary Material 7). Control mortality ranged from 12.5% for in-hospital endpoints to 42.4% for extended follow-up, while cancer-associated sepsis mortality ranged from 18.5% to 61.1%. The corresponding absolute risk differences were 3.0% for ICU, 6.0% for in-hospital, 13.4% for early, 13.7% for intermediate and 18.7% for extended follow-up, equivalent to approximately 30–187 excess deaths per 1,000 patients.

Across sensitivity analyses (Supplementary Material 8), results remained directionally consistent under varying methodological assumptions. Restriction to studies in which malignancy was the primary exposure also yielded directionally consistent estimates across all mortality endpoints. RVE-based pooled ORs were broadly consistent with the primary analysis across endpoints: ICU (OR 1.42, 95% CI 1.10–1.83), in-hospital (OR 1.89, 95% CI 1.69–2.12), early (OR 1.85, 95% CI 1.63–2.11), intermediate (OR 2.21, 95% CI 1.89–2.59) and extended follow-up (OR 2.74, 95% CI 1.95–3.86). Adjusted analyses showed more pronounced elevations for in-hospital (OR 2.16, 95% CI 1.78–2.63) and early mortality (OR 2.06, 95% CI 1.18–3.61). RR-based estimates also indicated consistent increases, though attenuated compared with ORs, as expected with high event rates. HR-based sensitivity analyses, available for fewer endpoints, were also directionally consistent with the primary findings. Restriction to Sepsis-1/2/3 cohorts yielded persistent associations, while excluding studies with >10,000 participants mainly affected in-hospital, early and intermediate outcomes, with modest reductions in heterogeneity.

### Mortality associations across subgroup analyses

#### Cancer subtype and disease extent

Table 2 summarizes all subgroup analyses. For cancer subtype and disease extent, 38 studies contributed 80 mortality estimates; 19 estimates were excluded because of unavailable raw event data (*n* = 8), insufficient data for meta-analysis (*n* = 9), or non-separable cancer subgroup categories (*n* = 2). Analyses included 23 solid tumour estimates (*n* = 18,766,724), 30 haematologic malignancy estimates (*n* = 16,663,335) and 8 metastatic disease estimates (*n* = 288,548). In subtype-specific analyses, solid tumours were associated with increased in-hospital, early and intermediate mortality (ORs 1.40–2.31), although ICU estimates were imprecise. Haematologic malignancies showed pronounced associations across all available endpoint categories, including ICU mortality (OR 1.72, 95% CI 1.13–2.62; *I*^2^ = 76.6%), in-hospital mortality (OR 1.68, 95% CI 1.48–1.91; *I*^2^ = 96.8%), early mortality (OR 1.95, 95% CI 1.22–3.13; *I*^2^ = 70.7%) and intermediate mortality (OR 3.74, 95% CI 2.27–6.15; *I*^2^ = 73.0%). Metastatic disease was associated with higher in-hospital mortality (OR 1.83, 95% CI 1.78–1.89; *I*^2^ = 57.3%) and early mortality (OR 2.88, 95% CI 1.42–5.82; *I*^2^ = 61.2%).

**Table 2.** Endpoint-specific subgroup analyses of the association between malignancy and crude mortality.

Mortality endpoints	ICU	In-hospital	Early (28/30-day)	Intermediate (60/90-day)	Extended follow-up (≥180-day)
Primary analysis	1.21 (0.94–1.56; 50.6)	1.90 (1.69–2.13; 97.2)	1.81 (1.59–2.06; 43.4)	2.28 (1.72–3.03; 88.8)	2.41 (1.70–3.42; 59.5)
Cancer subtypes					
Solid	1.40 (0.68–2.91; 85.6)	1.48 (1.42–1.54; 81.2)	1.50 (1.03–2.20; 68.8)	2.31 (1.06–5.03; 87.2)	–
Haematologic	1.72 (1.13–2.62; 76.6)	1.68 (1.48–1.91; 96.8)	1.95 (1.22–3.13; 70.7)	3.74 (2.27–6.15; 73.0)	–
Metastatic cancer	–	1.83 (1.78–1.89; 57.3)	2.88 (1.42–5.82; 61.2)	–	–
Septic shock only cohorts	1.32 (1.03–1.69; 34.2)	1.63 (1.39–1.92; 51.3)	1.57 (1.39–1.77; 39.0)	1.72 (1.30–2.27; 0.0)	–
Sepsis definition					
Sepsis-1	–	2.25 (1.85–2.75; 16.5)	1.91 (1.38–2.63; 0.0)	–	–
Sepsis-2	1.29 (0.94–1.78; 0.0)	2.15 (1.53–3.00; 75.7)	1.97 (1.58–2.46; 46.4)	2.54 (1.73–3.74; 89.8)	–
Sepsis-3	1.09 (0.58–2.09; 84.6)	1.92 (1.60–2.30; 86.8)	1.89 (1.49–2.39; 39.9)	3.05 (1.91–4.85; 49.6)	–
ICD-codes	–	1.63 (1.28–2.07; 99.3)	1.28 (0.78–2.10; 45.0)	–	–
Enrolment setting					
ICU-restricted	1.33 (1.07–1.65; 22.5)	2.17 (1.91–2.47; 61.1)	1.79 (1.51–2.11; 48.0)	2.14 (1.61–2.85; 89.7)	2.15 (1.49–3.10; 55.0)
General inpatient	–	1.80 (1.38–2.34; 99.1)	1.62 (1.20–2.20; 41.5)	–	–
ED-based	–	1.54 (1.26–1.87; 64.1)	2.13 (1.55–2.92; 45.1)	–	–
Mixed setting	–	1.57 (1.24–1.98; 0.0)	–	–	–
Geographic regions					
Asia	1.07 (0.69–1.64; 57.5)	2.01 (1.69–2.39; 98.4)	2.00 (1.66–2.41; 38.0)	1.36 (0.35–5.22; 84.7)	–
Europe	1.33 (0.96–1.84; 46.2)	1.80 (1.50–2.14; 60.5)	1.62 (1.27–2.05; 44.4)	2.35 (1.53–3.61; 86.4)	2.24 (1.36–3.70; 68.7)
North America	–	1.74 (1.48–2.04; 97.0)	–	–	–

*Notes:* Values are pooled odds ratios (ORs) with 95% CIs and *I*^2^ values (%). Cancer subtype estimates compare each subtype with its corresponding non-subtype comparison group within contributing studies, not necessarily with a pure non-cancer control group. ED: emergency department; CI: confidence interval; ICU: intensive care unit.

#### Septic shock only cohorts

Twenty studies contributed 31 mortality estimates, with heterogeneous septic shock definitions: Sepsis-1 in 3 studies, Sepsis-2 in 8, Sepsis-3 in 5, ICD-based definition in 1 and other definitions in 3. Five estimates were excluded because data were insufficient for endpoint-specific meta-analysis. The pooled analyses included 5 ICU, 8 in-hospital, 9 early and 4 intermediate estimates, comprising 2,755, 12,519, 4,035 and 3,890 participants, respectively. Pooled estimates indicated modest but consistent increases in mortality: ICU mortality (OR 1.32, 95% CI 1.03–1.69; *I*^2^ = 34.2%), in-hospital mortality (OR 1.63, 95% CI 1.39–1.92; *I*^2^ = 51.3%), early mortality (OR 1.57, 95% CI 1.39–1.77; *I*^2^ = 39.0%) and intermediate mortality (OR 1.72, 95% CI 1.30–2.27; *I*^2^ = 0.0%).

#### Sepsis definition

Of the 101 mortality estimates included in the primary analysis dataset, 15 were not pooled in the sepsis definition subgroup analysis because they belonged to other-definition categories (*n* = 8) or had insufficient data for endpoint-specific pooling (*n* = 7). Analyses included 13 Sepsis-1 estimates (*n* = 10,547), 32 Sepsis-2 estimates (*n* = 36,300), 29 Sepsis-3 estimates (*n* = 93,847) and 12 ICD-based estimates (*n* = 19,626,000). Sepsis-1 showed elevated associations for in-hospital mortality (OR 2.25, 95% CI 1.85–2.75; *I*^2^ = 16.5%) and early mortality (OR 1.91, 95% CI 1.38–2.63; *I*^2^ = 0.0%). Sepsis-2 demonstrated consistent increases across ICU, in-hospital, early and intermediate mortality (ORs 1.29–2.54). Sepsis-3 also showed elevated associations, with the highest estimate for intermediate mortality (OR 3.05, 95% CI 1.91–4.85; *I*^2^ = 49.6%). ICD-coded cohorts showed lower estimates but marked heterogeneity, particularly for in-hospital mortality (OR 1.63, 95% CI 1.28–2.07; *I*^2^ = 99.3%). In an additional post hoc comparison, mortality associations did not differ significantly between studies using Sepsis-1/2 and Sepsis-3 definitions for ICU (p for subgroup difference = 0.64), in-hospital (*p* = 0.21), early (*p* = 0.81), or intermediate mortality (*p* = 0.27) (Supplementary Material 9).

#### Enrolment setting

In the enrolment setting subgroup analysis, 6 mortality estimates were excluded from pooling because data were insufficient for endpoint-specific meta-analysis. Across 60 estimates from ICU-restricted studies (*n* = 123,197), malignancy was consistently associated with higher mortality, ranging from ICU mortality (OR 1.33, 95% CI 1.07–1.65; *I*^2^ = 22.5%) to extended follow-up mortality (OR 2.15, 95% CI 1.49–3.10; *I*^2^ = 55.0%). In 19 estimates from general inpatient studies (*n* = 19,641,366), in-hospital mortality was higher (OR 1.80, 95% CI 1.38–2.34; *I*^2^ = 99.1%), as was early mortality (OR 1.62, 95% CI 1.20–2.20; *I*^2^ = 41.5%), although heterogeneity was substantial. Thirteen estimates from ED-based studies (*n* = 23,354) showed higher early mortality (OR 2.13, 95% CI 1.55–2.92; *I*^2^ = 45.1%) and a more moderate association with in-hospital mortality (OR 1.54, 95% CI 1.26–1.87; *I*^2^ = 64.1%). Three estimates from mixed-setting studies (*n* = 3,254) showed moderate in-hospital associations (OR 1.57, 95% CI 1.24–1.98; *I*^2^ = 0.0%).

#### Geographic region

Eleven mortality estimates were excluded from the geographic region subgroup analysis because data were insufficient for endpoint-specific meta-analysis. The remaining 90 mortality estimates comprised 45 from Asian studies (*n* = 471,869), 36 from European studies (*n* = 47,017) and 9 from North American studies (*n* = 19,250,126). Across Asian studies, malignancy was associated with higher in-hospital mortality (OR 2.01, 95% CI 1.69–2.39; *I*^2^ = 98.4%) and early mortality (OR 2.00, 95% CI 1.66–2.41; *I*^2^ = 38.0%), although intermediate estimates were imprecise and heterogeneous (OR 1.36, 95% CI 0.35–5.22; *I*^2^ = 84.7%). In European studies, mortality estimates were elevated for in-hospital mortality (OR 1.80, 95% CI 1.50–2.14; *I*^2^ = 60.5%), early mortality (OR 1.62, 95% CI 1.27–2.05; *I*^2^ = 44.4%), intermediate mortality (OR 2.35, 95% CI 1.53–3.61; *I*^2^ = 86.4%) and extended follow-up mortality (OR 2.24, 95% CI 1.36–3.70; *I*^2^ = 68.7%). North American studies showed elevated in-hospital mortality (OR 1.74, 95% CI 1.48–2.04; *I*^2^ = 97.0%), but data for other endpoints were limited.

#### Surgical-only cohorts

Analyses were drawn from eight surgical sepsis studies, including three surgical ICU studies (*n* = 1,531) [53–55], two neurosurgical studies (*n* = 662) [56,57] and three peritonitis surgery studies (*n* = 3,563) [58–60]. Because these studies represented distinct surgical populations with heterogeneous infection sources, we did not derive an overall pooled estimate across all surgical studies. Malignancy was associated with higher in-hospital mortality in surgical ICU studies (OR 2.50, 95% CI 1.94–3.22; *I*^2^ = 0.0%). The association was also elevated in peritonitis surgery studies but was less precise and more heterogeneous (OR 2.26, 95% CI 0.98–5.19; *I*^2^ = 82.0%). Neurosurgical studies were summarized descriptively because only two estimates were available.

### Exploratory meta-regression analyses

Exploratory meta-regression analyses are summarized in Supplementary Materials 10 and 11. In univariable models, most study-level characteristics were not clearly associated with between-study heterogeneity. Among severity-related indicators, each 10% increase in ICU admission was associated with a larger mortality association (β = 0.029, 95% CI 0.003–0.056; *p* = 0.03; R^2^ = 16.8%), as was each 10% increase in mechanical ventilation (β = 0.059, 95% CI 0.010–0.109; *p* = 0.02; R^2^ = 20.2%). Among population-level covariates, each 10% increase in cancer proportion was inversely associated with the mortality association (β = −0.119, 95% CI −0.185 to −0.053; *p* < 0.001), explaining 21.0% of heterogeneity. In exploratory multivariable meta-regression, the main model including age, cancer proportion and sepsis definition explained 24.2% of between-study heterogeneity (QM *p* < 0.01; *k* = 72). Alternative models replacing sepsis definition with enrolment setting, geographic region, or analytic context showed broadly comparable explanatory performance, with R^2^ values of 24.4%, 32.6% and 22.9%, respectively. Adding severity-related covariates produced variable increases in explained heterogeneity, but the number of contributing studies differed substantially across models. R^2^ increased to 40.7% with ICU admission (*k* = 52), 30.1% with mechanical ventilation (*k* = 25), 53.5% with sequential organ failure assessment (SOFA) score (*k* = 35) and 72.0% with renal replacement therapy (*k* = 19), whereas the septic shock model explained less heterogeneity (R^2^ = 4.3%; *k* = 31).

### Exploratory cumulative meta-analysis

Cumulative meta-analysis by publication year is shown in Supplementary Material 12. Results suggested progressive stabilization of the accumulated evidence, with the pooled association between malignancy and sepsis mortality becoming statistically significant and remaining stable from 2006 onward (cumulative OR 2.42, 95% CI 1.46–4.01).

## Discussion

In this systematic review of 105 studies, endpoint-specific meta-analyses showed higher mortality estimates among adults with sepsis or septic shock and malignancy than among those without malignancy, although the magnitude and precision of associations varied across endpoints. Associations also varied across cancer-related characteristics and clinical contexts, highlighting substantial prognostic heterogeneity and the need for more granular characterization of malignancy in future studies. Descriptive analyses of raw mortality events further contextualized these findings, with absolute mortality differences ranging from 3.0% for ICU mortality to 18.7% for extended follow-up, equivalent to approximately 30–187 excess deaths per 1,000 patients. Because the primary analysis used unadjusted estimates to maximize study inclusion and comparability, the pooled results should be interpreted as overall prognostic associations rather than independent causal effects. Although reporting-bias assessments showed no consistent evidence of small-study effects across endpoints, major asymmetry suggested by the LFK index for in-hospital mortality warrants cautious interpretation.

Haematologic malignancy showed more sustained associations at later endpoints, whereas metastatic disease was more strongly associated with earlier mortality; solid tumours generally showed more modest increases across available endpoints. These findings may reflect differences in disease burden, immune reserve and vulnerability to recurrent infection or delayed recovery [61–64]. Because they were derived from separate subgroup and aggregate-level analyses, they should be interpreted as exploratory rather than definitive evidence of differences between cancer subgroups. Beyond cancer-related characteristics, exploratory meta-regression based primarily on in-hospital and early mortality data suggested that study-level acute severity indicators may partly explain between-study variability. Together, these findings indicate endpoint-specific variation in the malignancy–mortality association, potentially reflecting both cancer-related characteristics and acute illness context.

Although earlier estimates were more variable, the cumulative meta-analysis suggested greater statistical stability of the malignancy–mortality association as evidence accumulated after the mid-2000s. This stabilization coincided with broader standardization of sepsis recognition and management through the Surviving Sepsis Campaign and increasing use of large-scale epidemiologic data [65,66]. In this review, elevated estimates were observed across Sepsis-1, Sepsis-2 and Sepsis-3, suggesting that the association was not limited to a single diagnostic framework. Because studies using Sepsis-1/2 generally preceded those using Sepsis-3, differences by sepsis definition may be partly intertwined with temporal changes in sepsis management, cancer care and cohort construction. However, the additional Sepsis-1/2 versus Sepsis-3 analysis showed no evidence of subgroup differences across mortality endpoints, and study year was not associated with effect size in exploratory meta-regression. The 60/90-day intermediate window may also be clinically informative for evaluating sustained treatment effects beyond the acute phase, as illustrated by a recent phenotype-enriched sepsis trial [67] assessing outcomes through 90 days. Nevertheless, the 1976–2025 study period spans major advances in critical care and oncology, and residual era-related confounding cannot be excluded. Accordingly, pooled estimates should be interpreted as associations across heterogeneous clinical eras rather than as representing a single contemporary care context. In line with current interest in sepsis heterogeneity and phenotype-informed approaches [68,69], these findings support considering malignancy-related characteristics as relevant contextual factors in sepsis risk assessment and care planning. Future studies integrating detailed cancer characteristics with molecular or immunological host-response phenotyping may improve prognostic stratification and help inform future investigation of ICU triage and treatment strategies.

Variation in cohort entry may represent an important structural source of between-study variability. Methodological work in critical care [70] has shown that inappropriate time-zero handling can introduce time-related bias, especially in ICU-restricted studies requiring survival to cohort inclusion. In this review, ICU-restricted studies contributed to all mortality endpoints, whereas ED-based and general inpatient studies mainly informed in-hospital and early mortality, suggesting partial linkage between enrolment setting and outcome timeframe. Nevertheless, our classification was based only on reported enrolment setting and did not distinguish patients enrolled through the ED by illness severity, resuscitation status, or subsequent ICU transfer. Accordingly, ED-based studies should not be interpreted as representing a homogeneous earlier-stage or lower-severity population. This caution is consistent with prior sepsis research [71] showing that ED-presenting and hospital-presenting sepsis differ in clinical characteristics, care processes and outcomes. Because sepsis time zero is often operationally defined rather than self-evident [72], endpoint-specific estimates should be interpreted in light of differences in cohort construction and admission pathways rather than malignancy-related prognosis alone. Against this background, the lower malignancy-associated estimate for ICU mortality than for in-hospital mortality should not be interpreted as evidence of a survival benefit from ICU admission, because these estimates arose from different endpoints and cohort structures rather than a direct ICU-versus-ward comparison. Whether selected patients with malignancy and sepsis derive differential benefit from ICU admission, and whether this could inform triage or admission criteria, warrants further investigation.

Clinically, the observed variation across endpoint timing, cancer-related characteristics and enrolment context suggests that malignancy should be viewed as a context-dependent prognostic factor rather than a static comorbidity label in sepsis. Hospital-based mortality alone may not fully capture prognosis, particularly for patients with haematologic malignancy or metastatic disease, in whom elevated associations were most apparent at intermediate or non-hospital endpoints. Future prognostic frameworks should therefore incorporate both cancer-related factors and acute illness context, alongside the timing of outcome assessment. In ageing populations, care models may benefit from closer integration of oncology, critical care, infectious diseases and post-sepsis care to address both acute stabilization and longer-term recovery.

One important limitation concerns potential confounding by treatment-limitation and goals-of-care decisions. Orders such as do-not-resuscitate, transitions to palliative or comfort-focused care and other care-process choices may differ substantially between patients with and without malignancy [73]. These decisions can directly influence ICU and in-hospital mortality but were rarely reported or adjusted for in the included studies [74,75]. Their application may also vary according to cultural norms, national regulations and health-system practices across regions [76,77]. Therefore, the present meta-analysis cannot fully separate cancer-related biological vulnerability from differences in treatment-limitation practices, particularly for hospital-based mortality endpoints.

Several other limitations warrant consideration. As the evidence was predominantly observational, residual confounding cannot be excluded. Endpoint-specific mortality estimates were pooled across studies rather than tracked repeatedly within the same patient cohorts, so the resulting estimates represent study-level aggregation rather than patient-level dynamics. Most included studies treated malignancy as a baseline comorbidity and lacked detailed information on cancer activity, stage, or treatment status, limiting cancer-specific granularity and interpretation of stage- or treatment-specific differences. However, restriction to studies in which malignancy was the primary exposure yielded directionally consistent findings across mortality endpoints. Septic shock definitions were also heterogeneous and did not consistently require both vasopressor support and hyperlactatemia according to contemporary Sepsis-3 criteria, limiting inference for patients meeting the full contemporary septic shock definition. Extended follow-up data were sparse and synthesized timepoints varied considerably across studies, reducing the robustness of these estimates. Because most studies reported all-cause rather than sepsis-attributable mortality, later deaths may increasingly reflect cancer progression or other competing causes; thus, the contribution of the index septic episode cannot be isolated, and extended follow-up estimates should be interpreted as overall prognostic associations rather than sepsis-specific effects. Restriction to English-language publications may have introduced language bias. Risk of bias was assessed using the NOS, a widely applied tool for observational studies but not specifically tailored for prognostic factor reviews; future work may benefit from tools designed for prognostic factor studies, such as QUIPS [78]. Finally, no formal GRADE assessment was performed, which limits certainty grading of the evidence.

## Conclusions

In summary, malignancy was consistently associated with higher mortality among adults with sepsis or septic shock, with variation across mortality endpoints, cancer-related characteristics and clinical contexts. These findings support endpoint-specific prognostic assessment that considers cancer-related factors, acute illness context and enrolment setting, while highlighting the need for future studies to incorporate more granular cancer characteristics. Interpretation should consider potential confounding by treatment-limitation and goals-of-care decisions, as well as other care-process factors. Future studies should move beyond hospital mortality alone and incorporate longer-term and patient-centred outcomes, including resumption of anticancer therapy, functional recovery, recurrent infection, rehospitalization and post-sepsis care needs.

## Supplementary Material

Supplementary material.docx

## Data Availability

All data used in this study were obtained from previously published studies and are publicly available. Extracted data are available from the corresponding author upon reasonable request.
