# Supplementary material for: Endpoint-specific mortality associated with malignancy among adults with sepsis or septic shock: a systematic review and meta-analysis
Source: Ann Med. 2026 Oct 30;58(1):2752656. doi: 10.1080/07853890.2026.2752656 (PMC13726248; doi:10.1080/07853890.2026.2752656)
Supplement: Supplementary file 1 [file IANN_A_2752656_SM8617.docx]

**Endpoint-specific mortality associated with malignancy among adults with sepsis or septic shock: a systematic review and meta-analysis**

**Supplementary Material**

**Contents**

[**Supplementary Material 1.** PRISMA checklist.	2](#_Toc239778083)

[**Supplementary Material 2.** Protocol amendments and deviations.	4](#_Toc239778084)

[**Supplementary Material 3.** Search strategies.	6](#_Toc239778085)

[**Supplementary Material 4.** Study-level characteristics.	8](#_Toc239778086)

[**Supplementary Material 5.** Risk of bias assessment	31](#_Toc239778087)

[**Supplementary Material 6.** Publication bias assessment.	39](#_Toc239778088)

[**Supplementary Material 7.** Descriptive absolute mortality among studies reporting raw event counts.	41](#_Toc239778089)

[**Supplementary Material 8.** Sensitivity analyses.	42](#_Toc239778090)

[**Supplementary Material 9.** Sepsis-1/2 versus Sepsis-3 subgroup analysis.	43](#_Toc239778091)

[**Supplementary Material 10.** Exploratory univariable meta-regression analyses.	44](#_Toc239778092)

[**Supplementary Material 11.** Exploratory multivariable meta-regression analyses.	45](#_Toc239778093)

[**Supplementary Material 12.** Cumulative meta-analysis.	48](#_Toc239778094)

[**References**	49](#_Toc239778095)

**Supplementary Material 1.** PRISMA checklist.

| **Section and Topic** | **Item #** | **Checklist item** | **Location where item is reported** |
| --- | --- | --- | --- |
| **TITLE** | | |  |
| Title | 1 | Identify the report as a systematic review. | P1 |
| **ABSTRACT** | | |  |
| Abstract | 2 | See the PRISMA 2020 for Abstracts checklist. | P3-4 |
| **INTRODUCTION** | | |  |
| Rationale | 3 | Describe the rationale for the review in the context of existing knowledge. | P5-6 |
| Objectives | 4 | Provide an explicit statement of the objective(s) or question(s) the review addresses. | P6 |
| **METHODS** | | |  |
| Eligibility criteria | 5 | Specify the inclusion and exclusion criteria for the review and how studies were grouped for the syntheses. | P7 |
| Information sources | 6 | Specify all databases, registers, websites, organisations, reference lists and other sources searched or consulted to identify studies. Specify the date when each source was last searched or consulted. | P6 |
| Search strategy | 7 | Present the full search strategies for all databases, registers and websites, including any filters and limits used. | Supplementary Material 3 |
| Selection process | 8 | Specify the methods used to decide whether a study met the inclusion criteria of the review, including how many reviewers screened each record and each report retrieved, whether they worked independently, and if applicable, details of automation tools used in the process. | P7 |
| Data collection process | 9 | Specify the methods used to collect data from reports, including how many reviewers collected data from each report, whether they worked independently, any processes for obtaining or confirming data from study investigators, and if applicable, details of automation tools used in the process. | P8 |
| Data items | 10a | List and define all outcomes for which data were sought. Specify whether all results that were compatible with each outcome domain in each study were sought (e.g. for all measures, time points, analyses), and if not, the methods used to decide which results to collect. | P8 |
|  | 10b | List and define all other variables for which data were sought (e.g. participant and intervention characteristics, funding sources). Describe any assumptions made about any missing or unclear information. | P8 |
| Study risk of bias assessment | 11 | Specify the methods used to assess risk of bias in the included studies, including details of the tool(s) used, how many reviewers assessed each study and whether they worked independently, and if applicable, details of automation tools used in the process. | P8 |
| Effect measures | 12 | Specify for each outcome the effect measure(s) (e.g. risk ratio, mean difference) used in the synthesis or presentation of results. | P9 |
| Synthesis methods | 13a | Describe the processes used to decide which studies were eligible for each synthesis (e.g. tabulating the study intervention characteristics and comparing against the planned groups for each synthesis (item #5)). | P8-9 |
|  | 13b | Describe any methods required to prepare the data for presentation or synthesis, such as handling of missing summary statistics, or data conversions. | P8-9 |
|  | 13c | Describe any methods used to tabulate or visually display results of individual studies and syntheses. | P9 |
|  | 13d | Describe any methods used to synthesize results and provide a rationale for the choice(s). If meta-analysis was performed, describe the model(s), method(s) to identify the presence and extent of statistical heterogeneity, and software package(s) used. | P9 |
|  | 13e | Describe any methods used to explore possible causes of heterogeneity among study results (e.g. subgroup analysis, meta-regression). | P10 |
|  | 13f | Describe any sensitivity analyses conducted to assess robustness of the synthesized results. | P10 |
| Reporting bias assessment | 14 | Describe any methods used to assess risk of bias due to missing results in a synthesis (arising from reporting biases). | P9 |
| Certainty assessment | 15 | Describe any methods used to assess certainty (or confidence) in the body of evidence for an outcome. | P8 |
| **RESULTS** | | |  |
| Study selection | 16a | Describe the results of the search and selection process, from the number of records identified in the search to the number of studies included in the review, ideally using a flow diagram. | P10-11  Figure 1 |
|  | 16b | Cite studies that might appear to meet the inclusion criteria, but which were excluded, and explain why they were excluded. | Figure 1 |
| Study characteristics | 17 | Cite each included study and present its characteristics. | Table 1; Supplementary Material 4 |
| Risk of bias in studies | 18 | Present assessments of risk of bias for each included study. | P12;  Supplementary Material 5 |
| Results of individual studies | 19 | For all outcomes, present, for each study: (a) summary statistics for each group (where appropriate) and (b) an effect estimate and its precision (e.g. confidence/credible interval), ideally using structured tables or plots. | Supplementary Material 4 |
| Results of syntheses | 20a | For each synthesis, briefly summarise the characteristics and risk of bias among contributing studies. | P12; P15-17 |
|  | 20b | Present results of all statistical syntheses conducted. If meta-analysis was done, present for each the summary estimate and its precision (e.g. confidence/credible interval) and measures of statistical heterogeneity. If comparing groups, describe the direction of the effect. | Figure 2 |
|  | 20c | Present results of all investigations of possible causes of heterogeneity among study results. | P20-23; P25;  Table 2;  Supplementary Material 9-11 |
|  | 20d | Present results of all sensitivity analyses conducted to assess the robustness of the synthesized results. | P16-17;  Supplementary Material 8 |
| Reporting biases | 21 | Present assessments of risk of bias due to missing results (arising from reporting biases) for each synthesis assessed. | P16;  Supplementary Material 6 |
| Certainty of evidence | 22 | Present assessments of certainty (or confidence) in the body of evidence for each outcome assessed. | Formal GRADE assessment was not performed due to heterogeneity and observational design |
| **DISCUSSION** | | |  |
| Discussion | 23a | Provide a general interpretation of the results in the context of other evidence. | P26 |
|  | 23b | Discuss any limitations of the evidence included in the review. | P26-31 |
|  | 23c | Discuss any limitations of the review processes used. | P29-31 |
|  | 23d | Discuss implications of the results for practice, policy, and future research. | P29 |
| **OTHER INFORMATION** | | |  |
| Registration and protocol | 24a | Provide registration information for the review, including register name and registration number, or state that the review was not registered. | P6 |
|  | 24b | Indicate where the review protocol can be accessed, or state that a protocol was not prepared. | P6 |
|  | 24c | Describe and explain any amendments to information provided at registration or in the protocol. | P6; Supplementary Material 2 |
| Support | 25 | Describe sources of financial or non-financial support for the review, and the role of the funders or sponsors in the review. | P32-33 |
| Competing interests | 26 | Declare any competing interests of review authors. | P32 |
| Availability of data, code and other materials | 27 | Report which of the following are publicly available and where they can be found: template data collection forms; data extracted from included studies; data used for all analyses; analytic code; any other materials used in the review. | P33 |

**Supplementary Material 2.** Protocol amendments and deviations.

| **Protocol domain** | **Original** | **Change** | **Rationale** | **Timing classification** | **Protocol version** |
| --- | --- | --- | --- | --- | --- |
| Review objectives | Cancer vs non-cancer mortality comparison | Original objective retained; clarified as endpoint-specific prognostic associations across mortality endpoints and clinical populations. | Mortality endpoints varied substantially across studies | Protocol-consistent refinement | PROSPERO v4.0, 25 May 2026 |
| Population | Not specified in the original protocol | Excluded HIV-associated sepsis cohorts; retained general adult cohorts with <5% HIV-positive patients or separable results. | To clarify eligibility criteria and reduce heterogeneity from populations with distinct clinical characteristics | Registered amendment | PROSPERO v2.0, 11 Dec 2025 |
| Population | Not specified in the original protocol | Clarified predominantly adult and non-HIV eligibility using reported data or clinical context. | To clarify population eligibility and reduce clinical heterogeneity | Registered amendment | PROSPERO v3.0, 10 Mar 2026 |
| Risk-of-bias assessment | Cochrane RoB-1 and NOS selected | Cochrane RoB 2 for randomized trials; NOS retained for cohort/case-control studies; adapted NOS manually clarified for cross-sectional studies. | Cochrane RoB 2 updated; PROSPERO NOS field did not distinguish adapted versions by study design | Protocol-consistent update and refinement | PROSPERO v4.0, 25 May 2026 |
| Reporting bias assessment | Doi plot and LFK | Doi plots and LFK index retained; funnel plots and Egger’s regression test added. | To align with protocol and standard approaches | Protocol-consistent supplement | PROSPERO v4.0, 25 May 2026 |
| Main outcomes | All-cause infectious mortality | All-cause mortality | Most studies did not reliably distinguish infectious from all-cause death | Post-registration deviation | PROSPERO v4.0, 25 May 2026 |
| Strategy for data synthesis | Most fully adjusted estimates prioritized | Crude or unadjusted estimates were prioritized for the main analysis; adjusted estimates were evaluated in sensitivity analyses where available | Adjusted covariates varied across studies; crude data were more consistently available and improved comparability. | Post-registration deviation | PROSPERO v4.0, 25 May 2026 |
| Strategy for data synthesis | Risk ratios manually calculated from crude mortality data | Odds ratios used as the primary common effect measure; risk ratios used in sensitivity analyses. | Odds ratios allowed inclusion of additional model-based estimates. | Post-registration deviation | PROSPERO v4.0, 25 May 2026 |
| Strategy for data synthesis | MetaXL v5.3 and SPSS Statistics 22 | RStudio and SPSS Statistics. | RStudio supported flexible meta-analytic methods, sensitivity analyses, and prediction intervals. | Post-registration deviation | PROSPERO v4.0, 25 May 2026 |
| Exploratory analyses | Not specified in the original protocol | Additional endpoint‑specific, subgroup, and sensitivity analyses were added and interpreted as exploratory. | To examine heterogeneity, temporal stability, influence of individual studies, and endpoint-specific patterns. | Post hoc exploratory | PROSPERO v4.0, 25 May 2026 |
| Additional exploratory analyses | Not specified in the original protocol | Added a Sepsis-1/2 vs Sepsis-3 subgroup analysis and a sensitivity analysis restricted to studies with malignancy as the primary exposure. | To assess heterogeneity by sepsis definition and robustness to cancer analytic context. | Post hoc exploratory | Within the scope of PROSPERO v4.0 |

NOS, Newcastle-Ottawa Scale; RoB, Risk of Bias.

**Supplementary Material 3.** Search strategies.

**PubMed (from database inception to September 16, 2025)**

| #1 | "Sepsis"[Mesh] OR "Shock, Septic"[Mesh] OR sepsis OR "severe sepsis" OR "septic shock" |
| --- | --- |
| #2 | neoplasms, Second Primary [Mesh] OR neoplasms [MeSH] OR carcinoma [Mesh] OR cancer* OR tumor* OR tumour* OR neoplasm* OR malignanc* OR carcinoma* OR "hematologic malignancy" OR "solid tumor" |
| #3 | "Mortality"[Mesh] OR "Survival"[Mesh] OR Life expectancy [Mesh] OR analysis, survival [Mesh] OR mortality OR death OR survival OR "life expectancy" OR "standardized mortality ratio*" OR SMR OR "case fatality rate" OR "hospital mortality" OR "ICU mortality" |
| #4 | #1 AND #2 AND #3 |
| #5 | Filters: English, Humans |

**Embase (from database inception to September 16, 2025)**

| #1 | 'sepsis'/exp OR 'septic shock'/exp OR sepsis:ti,ab OR 'severe sepsis':ti,ab OR 'septic shock':ti,ab |
| --- | --- |
| #2 | 'neoplasm'/exp OR cancer*:ti,ab OR tumor*:ti,ab OR tumour*:ti,ab OR malignanc*:ti,ab OR  carcinoma*:ti,ab OR 'hematologic malignancy':ti,ab OR 'solid tumor':ti,ab |
| #3 | 'mortality'/exp OR 'survival'/exp OR mortality:ti,ab OR death:ti,ab OR survival:ti,ab OR survive:ti,ab OR 'life expectancy':ti,ab OR 'standardized mortality ratio*':ti,ab OR smr:ti,ab OR 'case fatality rate':ti,ab OR 'hospital mortality':ti,ab OR 'ICU mortality':ti,ab |
| #4 | #1 AND #2 AND #3 |
| #5 | [english]/lim AND [humans]/lim |

**Web of Science (from database inception to September 16, 2025)**

| #1 | sepsis OR "severe sepsis" OR "septic shock" (Topic) |
| --- | --- |
| #2 | cancer* OR tumor* OR tumour* OR neoplasm* OR malignanc* OR carcinoma* OR "hematologic malignancy" OR "solid tumor" (Topic) |
| #3 | mortality OR death OR survival OR survive OR "life expectancy" OR "standardized mortality ratio*" OR SMR OR "case fatality rate" OR "hospital mortality" OR "ICU mortality" (Topic) |
| #4 | #1 AND #2 AND #3 |
| #5 | English (Languages) |

**Cochrane library (from database inception to September 16, 2025)**

| #1 | (sepsis OR "severe sepsis" OR "septic shock") in Title Abstract Keyword |
| --- | --- |
| #2 | (cancer* OR tumor* OR tumour* OR neoplasm* OR malignanc* OR carcinoma* OR "hematologic malignancy" OR "solid tumor") in Title Abstract Keyword |
| #3 | (mortality OR death OR survival OR survive OR "life expectancy" OR "standardized mortality ratio*" OR SMR OR "case fatality rate" OR "hospital mortality" OR "ICU mortality") in Title Abstract Keyword |
| #4 | #1 AND #2 AND #3 |

**Epistemonikos (from database inception to September 16, 2025)**

| #1 | title:(sepsis OR "severe sepsis" OR "septic shock") OR abstract:(sepsis OR "severe sepsis" OR "septic shock") |
| --- | --- |
| #2 | title:(cancer* OR tumor* OR tumour* OR neoplasm* OR malignanc* OR carcinoma* OR "hematologic malignancy" OR "solid tumor") OR abstract:(cancer* OR tumor* OR tumour* OR neoplasm* OR malignanc* OR carcinoma* OR "hematologic malignancy" OR "solid tumor") |
| #3 | title:(mortality OR death OR survival OR survive OR "life expectancy" OR "standardized mortality ratio*" OR SMR OR "case fatality rate" OR "hospital mortality" OR "ICU mortality") OR abstract:(mortality OR death OR survival OR survive OR "life expectancy" OR "standardized mortality ratio*" OR SMR OR "case fatality rate" OR "hospital mortality" OR "ICU mortality") |
| #4 | #1 AND #2 AND #3 |

**Supplementary Material 4.** Study-level characteristics.

**Table S4-1.** Baseline characteristics of primary studies.

| **Study** | **Country/ Territory** | **Study design** | **Study scope** | **Enrollment setting** | **Study year** | **Sepsis definition** | **Clinical population** | **Cancer type** | **Mortality endpoint** | **Sample size** | **Age** | **Female**  **(%)** | **Cancer**  **(%)** |
| --- | --- | --- | --- | --- | --- | --- | --- | --- | --- | --- | --- | --- | --- |
| Acar (2024) [1] | Turkey | Cohort | Single-center | ICU | 2013-2021 | Sepsis-3 | Sepsis | Solid, hema | 30-day | 245 | 60.9 ± 17.1 | 50.6 | 24.9 |
| Ahlström (2022) [2] | Sweden | Cohort | Population-based | ICU | 2011-2016 | Sepsis-2 | Sepsis | Cancer, hema | 60-day | 22,354 | 69.3 ± 13.3 | 42.5 | 10.10 |
| Al Omar (2024) [3] | Jordan | Cohort | 2 hospitals | ICU | 2022 | Sepsis-3 | Sepsis | Solid, hema | 30-day | 148 | 66.1 ± 17.5 | 54.7 | 14.2 |
| Artero (2010) [4] | Spain | Cohort | Single-center | ICU | 1998-2008 | Sepsis-1 | Severe sepsis and septic shock | Cancer | In-hospital | 112 | 63.5 ± 15.8 | 40.2 | 8.9 |
| Beck (2014) [5] | Canada, USA, Saudi Arabia | Cohort | Multinational, 28 institutions | ICU | 1996-2008 | Sepsis-1 | Septic shock | Hema, meta | In-hospital | 6,514 | 62.1 ± 16.1 | 43.0 | 17.7 |
| Boonmee (2020) [6] | Thailand | Cohort | Single-center | ED-based | 2018-2019 | Sepsis-3 | Sepsis | Cancer | In-hospital | 1,616 | 72.6 ± 15.4 | 51.1 | 22.7 |
| Burnham (2015) [7] | USA | Cohort | Single-center | General inpatient | 2009-2013 | ICD-9 | Enterobacterales sepsis | Solid, hema | 30-day | 510 | 60.0 ± 15.7 | 47.6 | 52.8 |
| Busani (2017) [8] | Italy | Cohort | Single-center | ICU | 2008-2013 | Sepsis-2 | Septic shock caused by MDR bacteria | Cancer | 30-day | 94 | 70.9 ± 12.2 | 38.3 | 24.5 |
| Camou (2020) [9] | France | Cohort | Single-center | ICU | 2012-2016 | Sepsis-2 | Septic shock | Solid, hema | ICU,  28/180-day | 435 | 65.1 ± 15.0 | 43.4 | 57.9 |
| Cebeci (2025) [10] | Turkey | Cohort | Single-center | ICU | 2018-2023 | Sepsis-3 | Sepsis | Solid | 28-day | 446 | 72 (18-99) | 44.8 | 26.2 |
| Chebl (2021) [11] | Lebanon | Cohort | Single-center | ED-based | 2018-2021 | Sepsis-3 | Sepsis | Cancer | In-hospital | 939 | 72.4 ± 15.6 | 40.9 | 39.6 |
| Chen (2014) [12] | Taiwan | Cohort | Single-center | ICU | 2004-2005 | Sepsis-1 | Severe sepsis | Active cancer | In-hospital | 254 | 67.3 ± 14.9 | 37.4 | 12.6 |
| Chen (2022) [13] | Taiwan | Cohort | Single-center | Surgical ICU | 2009-2011 | Sepsis-2 | Sepsis | Cancer | 1-year | 739 | 64.8 ± 15.3 | 31.8 | 36.3 |
| Cheng (2007) [14] | China | Cohort | 10 hospitals | Surgical ICU | 2004-2005 | Sepsis-2 | Severe sepsis | Cancer | In-hospital | 318 | 61.7 ± 20.0 | 35.2 | 12.0 |
| Cooper (2020) [15] | USA | Cohort | Single-center | General inpatient | 2003-2014 | Modified Sepsis-3 | Sepsis | Solid, hema | In-hospital | 20,975 | 61.4 ± 17.0 | 44.8 | 35.7 |
| Fabbri (2019) [16] | Italy | Cohort | Single-center | ED-based | 2009-2016 | ICD-9  Sepsis-3 | Sepsis | Cancer | In-hospital | 2,492 | 77.9 ± 14.6 | 45.6 | 42.0 |
| Fang (2017) [17] | Taiwan | Cohort | Single-center | ICU | 2013-2016 | Sepsis-2 Sepsis-3 | Severe sepsis and septic shock | Active cancer | 14/28/90-day | 532 | 66.5 ± 15.2 | 41.0 | 17.9 |
| Firempong (2014) [18] | USA | Cohort | Statewide based | General inpatient | 2001-2009 | Sepsis-2 | Post-neurosurgical septic shock | Cancer | In-hospital | 557 | 61.8 ± 17.2 | 41.5 | 26.8 |
| Gameiro (2018) [19] | Portugal | Cohort | Single-center | ICU | 2008-2014 | Sepsis-3 | Sepsis | Cancer | In-hospital | 456 | 64.1 ± 16.4 | 42.1 | 23.9 |
| García G.-R. (2024) [20] | Spain | Cohort | Single-center | ICU | 2015-2016 | Sepsis-2 Sepsis-3 | Sepsis | Active cancer | 28-day | 173 | 66.0 ± 15.6 | 37.0 | 27.8 |
| Garnacho M. (2020) [21] | Spain | Cohort | Single-center | ICU | 2015-2017 | Sepsis-3 | Sepsis | Cancer | In-hospital | 55 | 61.3 ± 17.0 | 40.0 | 16.4 |
| Gonzalez (2016) [22] | France | Cohort | Single-center | ICU | 2009-2014 | Sepsis-2 | Septic shock | Cancer, hema | ICU | 223 | 64.0 ± 13.0 | 37.2 | 15.7 |
| Hall (2015) [23] | UK | RCT | Single-center | ICU  HDU | 2010-2012 | Sepsis-2 | Sepsis | Cancer | In-hospital | 60 | 64.2 ± 12.5 | 45.0 | 28.3 |
| He (2023) [24] | China | Cohort | Single-center | ICU | 2019-2022 | Sepsis-3 | Sepsis | Cancer | In-hospital | 830 | 62.3 ± 15.8 | 37.2 | 18.4 |
| Holmbom (2022) [25] | Sweden | Cohort | County-level | General inpatient | 2019-2020 | ICD-10  Culture | Community-onset urosepsis | Cancer | 30-day | 282 | 72.0 ± 14.0 | 41.8 | 17.7 |
| Hsieh (2021) [26] | Taiwan | Cohort | Population-based | General inpatient | 1999-2013 | ICD-9 and antibiotics use | Sepsis with T2DM | Cancer | In-hospital | 55,860 | 68.8 ± 13.1 | 48.0 | 27.2 |
| Huang (2019a) [27] | Taiwan | Cohort | Single-center | ICU | 2010-2014 | Sepsis-3 | Sepsis with APACHE II≦15 | Cancer | In-hospital | 352 | 64.6 ± 18.2 | 40.1 | 17.3 |
| Huang (2019b) [28] | Taiwan | Cohort | Single-center | ICU | 2018 | Sepsis-3 | Septic shock | Cancer | 28-day | 104 | 66.7 ± 14.7 | 32.7 | 40.4 |
| Inchai (2015) [29] | Thailand | Cohort | Single-center | ICU | 2005-2011 | Sepsis-2 | Severe sepsis and septic shock with VAP | Cancer | 30-day | 621 | 62.2 ± 18.1 | 46.4 | 12.4 |
| Jamme (2017) [30] | France | Cohort | Single-center | ICU | 2008-2015 | Sepsis-3 | Septic shock | Solid, hema | 3-day, ICU, in-hospital | 724 | 68.7 ± 16.1 | 35.2 | 31.5 |
| Jouffroy (2023) [31] | France | Cohort | Multicenter | ICU | 2016-2021 | Sepsis-2 | Septic shock | Cancer history | 30-day | 530 | 69.0 ± 15.0 | 35.7 | 35.1 |
| Kang (2024) [32] | South Korea | Cohort | Nationwide | General inpatient | 2011-2016 | ICD-10 | Sepsis | Solid, hema, meta | In-hospital | 373,539 | 68.9 ± 15.3 | 50.0 | 30.7 |
| Kattouf (2022) [33] | USA | Cohort | Nationwide | General inpatient | 2017 | Medical records | Septic trauma | Meta | In-hospital | 1,782 | 56.2 ± 19.6 | 27.1 | 1.0 |
| Khwannimit (2009) [34] | Thailand | Cohort | Single-center | ICU | 2004-2006 | Sepsis-1 | Severe sepsis and septic shock | Hema, meta | In-hospital | 390 | 56.0 ± 23.7 | 43.1 | 19.5 |
| Kikuchi (2024) [35] | Japan | Cohort | Single-center | ICU | 2005-2019 | Sepsis-3 | Sepsis | Solid, hema | ICU | 496 | 65.4 ± 15.1 | 35.7 | 36.3 |
| Kim (2018) [36] | South Korea | Cohort | Single-center | ICU | 2013-2015 | Sepsis-1 | Septic shock | Solid, hema, meta | 28-day | 72 | 69.6 ± 10.7 | 41.7 | 43.1 |
| Kim (2022) [37] | South Korea | Cohort | Single-center | ICU | 2013-2021 | Sepsis-1 | Sepsis-induced AKI (receiving CRRT) | Solid, hema | 28-day | 649 | 69.5 ± 13.8 | 39.0 | 26.2 |
| Koçak (2025) [38] | Turkey | Cohort | Single-center | ICU | 2020-2024 | Sepsis-3 | Sepsis in geriatrics | Solid (hema excluded) | 28-day | 247 | 77.3 ± 8.8 | 41.7 | 19.0 |
| Korkmaz (2017) [39] | Turkey | Cohort | Single-center | ICU | 2014-2015 | Sepsis-2 | Sepsis | Cancer | ICU | 114 | 66.5 ± 14.7 | 50.0 | 15.8 |
| Kung (2012) [40] | Taiwan | Cohort | Single-center | ED-based | 2011 | Sepsis-2 | Severe sepsis and septic shock | Cancer | In-hospital | 67 | 64.8 ± 13.8 | 34.3 | 20.9 |
| Kuperman (2013) [41] | USA | Cohort | Single-center | ED-based | 2007-2010 | ICD-9 | Sepsis | Cancer | In-hospital | 792 | 61.9 ± 17.2 | 45.7 | 45.0 |
| Le Borgne (2022) [42] | France | Cohort | Single-center | ICU | 2013-2019 | ICD-10 | Septic shock with candidemia | Cancer, solid, hema | ICU, 28/90/180-day, in-hospital, 1-year | 239 (ICU stays) | 66.6 ± 15.3 | 40.5 | 61.9 |
| Lee (2013) [43] | South Korea | Cohort | Single-center | ED-based | 2010-2011 | Sepsis-1 | Sepsis in geriatrics | Cancer, meta | 28-day | 401 | 74.0 ± 7.4 | 50.1 | 25.4 |
| Lee (2025) [44] | South Korea | Cohort | 20 hospitals | General inpatient | 2019-2021 | Sepsis-3 | Fungal sepsis | Solid, hema, both | ICU, In-hospital | 407 | 70.8 ± 13.6 | 47.7 | 38.3 |
| Li (2021) [45] | China | Cohort | Single-center | ICU | 2015-2020 | Sepsis-3 | Sepsis | Cancer history | 28-day | 274 | 57.7 ± 10.7 | 38.7 | 4.7 |
| Lin (2022) [46] | Taiwan | Cohort | Single-center | ICU | 2013-2016 | Sepsis-3 | Sepsis-induced AKI | Cancer | In-hospital | 388 | 67.6 ± 15.1 | 38.4 | 25.3 |
| Liu (2025) [47] | China | Cohort | 5 hospitals | ICU  ED-based | 2015-2023 | Sepsis-3 | Sepsis | Cancer | In-hospital | 2,974 | 69.5 ± 15.5 | 62.5 | 18.5 |
| López (2021) [48] | Chile | Cohort | Single-center | ICU | 2017-2019 | Sepsis-3 | Sepsis | Cancer, solid, hema, meta | 90-day | 251 | 64.7 ± 18.4 | 43.0 | 31.9 |
| López M. (2018) [49] | Spain | Cohort | Single-center | General inpatient | 2011-2015 | Sepsis-2 | Hospital-acquired sepsis | Cancer | 28-day | 196 | Median, 73 | 31.6 | 34.2 |
| M. Lessa (2024) [50] | Brazil | Cohort | Single-center | ICU | 2018-2019 | Sepsis-1, 2, 3 | Sepsis | Cancer, hema | 28-day | 653 | 63.0 ± 11.9 | 46.7 | 25.3 |
| Macphail (2024) [51] | Aus/NZ | Cohort | Binational | ICU | 2000-2022 | Sepsis-3 | Sepsis | Hema | In-hospital/ICU | 282,627 | 65.6 ± 17.3 | 43.9 | 6.1 |
| Maia (2023) [52] | Brazil | Cohort | Single-center | ICU | 2019-2020 | Sepsis-3 | Sepsis | Cancer | In-hospital | 864 | 62.5 ± 21.3 | 56.1 | 18.9 |
| Man (2022) [53] | Hong Kong | Cohort | Single-center | ICU | 2010-2019 | Diagnosis and culture | Sepsis | Active cancer, hema | 30-day/ ICU/ In-hospital/1-year | 522 | 69.7 ± 14.1 | 35.2 | 50.4 |
| Matos (2025) [54] | USA | Cohort | Nationwide | ICU | 2008-2019 | Sepsis-3 | Sepsis | Cancer | In-hospital | 58,988 | 66.7 ± 17.0 | 45.6 | 10.4 |
| Mazzone (2016) [55] | Italy | Cohort | Regional multicenter | ICU | 2012 | Sepsis-2 | Sepsis | Solid, hema | In-hospital | 533 | 73.3 ± 14.6 | 50.8 | 23.6 |
| Musat (2025) [56] | Romania | Cohort | Single-center | ED-based | 2007-2024 | Medical records | Sepsis | Cancer, solid, hema | In-hospital | 12,089 | 68.7 ± 16.0 | 49.1 | 12.9 |
| Nam (2019) [57] | South Korea | Cohort | Single-center | ICU | 2013-2016 | Sepsis-3 | Sepsis | Cancer | 28-day | 125 | 70.7 ± 15.6 | 46.4 | 8.0 |
| N. Panday (2020) [58] | Netherlands | RCT | 34 hospitals | ED-based | 2014-2016 | Sepsis-2 | Sepsis | Cancer, solid, hema | In-hospital, 28/90-day | 2,658 | 72.8 ± 13.9 | 42.5 | 13.4 |
| Nielsen (2025) [59] | Denmark | Cohort | Single-center | ED-based | 2017-2018 | Sepsis-3 | Sepsis | Cancer | 2-year | 714 | 75.2 ± 13.3 | 41.6 | 14.0 |
| Nygård (2014) [60] | Norway | Cohort | Single-center | ICU  HDU | 2008 | Sepsis-2 | Community acquired severe sepsis | Cancer | In-hospital | 220 | 63.6 ± 18.7 | 46.8 | 14.1 |
| Oliveira (2002) [61] | Brazil | RCT | Single-center | ICU | — | Sepsis-2 | Severe sepsis | Cancer | In-hospital | 29 | 46.0 ± 13.1 | 37.9 | 20.7 |
| Oliveros (2024) [62] | Colombia | Cohort | Population-based | ICU | 2019 | ICD-10 | Sepsis | Cancer, meta | 1-year | 12,056 | 64.8 ± 18.7 | 51.9 | 8.6 |
| Orak (2018) [63] | Turkey | Cohort | Single-center | ED | 2012-2015 | Medical records | Sepsis | Cancer | In-hospital | 330 | 62.8 ± 19.4 | 44.2 | 12.4 |
| Orsetti (2019) [64] | Italy | Cohort | Single-center | General inpatient | 2010-2016 | — | Septic shock with candidemia | Solid | 30-day | 56 | — | — | 26.8 |
| Pansirit. (2022) [65] | Thailand | Cohort | Single-center | ED-based | 2016-2020 | Sepsis-3 | ED incubated sepsis | Cancer | 28-day | 344 | 69.2 ± 15.5 | 45.6 | 10.2 |
| Park (2012) [66] | South Korea | Cohort | 12 hospitals | General inpatient | 2005-2009 | Sepsis-1 | Severe sepsis and septic shock | Cancer | In-hospital | 1,192 | 65.0 ± 14.2 | 45.0 | 14.7 |
| Paulsen (2015) [67] | Norway | Cohort | County-level | General inpatient | 1996-2011 | Sepsis-2 | Staphylococcus aureus sepsis | Cancer | 30-day | 373 | 68.5 ± 17.5 | 39.7 | 25.7 |
| Pittet (1993) [68] | Switzerland | Cohort | Single-center | Surgical ICU | 1984-1988 | Sepsis-1 | Sepsis | Cancer | In-hospital | 173 | 50.0 ± 13.7 | 32.4 | 9.8 |
| Proaño-Z. (2023) [69] | USA | Cohort | Nationwide | General inpatient | 2013-2017 | NSQIP defined | Sepsis in older surgical patients | Meta | 30-day | 1,339 | 73.7 ± 7.4 | 54.7 | 6.7 |
| Qu (2021) [70] | China | Cohort | 16 hospitals | ICU | 2014-2015 | Sepsis-3 | Sepsis | Cancer | 28-day | 1,194 | 67.8 ± 18.4 | 34.4 | 9.1 |
| Rannikko (2017) [71] | Finland | Cohort | Single-center | ED-based | 2012-2014 | Sepsis-2 | Blood culture-positive sepsis | Hema, meta | 90-day | 497 | 68 (16-95) | 47.3 | 20.5 |
| Ravetti (2015) [72] | Brazil | Cohort | Single-center | ICU | 2012-2014 | Sepsis-2 | Severe sepsis and septic shock | Cancer, solid, hema | ICU, 28-day | 75 | 67.0 ± 17.9 | 25.3 | 53.3 |
| Reaven (2022) [73] | USA | Cohort | 5 hospitals | ED-based | 2014-2018 | Modified Sepsis-3 | Sepsis | Cancer | 3-day | 2,414 | 65.6 ± 16.3 | 48.6 | 20.7 |
| Riché (2009) [74] | France | Cohort | Single-center | Surgical ICU | 6 years | Sepsis-1 | Peritonitis-associated sepsis | Cancer | 30-day | 180 | 62.0 ± 18.0 | 46.1 | 25.0 |
| Ripa (2017) [75] | Spain | Cohort | Single-center | General inpatient | 2010-2015 | Sepsis-2/3 | Septic shock | Solid, hema | 7-day | 576 | 64.8 ± 16.9 | 42.0 | 37.3 |
| Roh (2019) [76] | South Korea | Cohort | Single-center | ED-based | 2014-2017 | Sepsis-3 | Sepsis | Hema | 180-day, 1-year | 446 | 61.3 ± 57.0 | 39.2 | 16.8 |
| Rotter (2020) [77] | USA | Cross-sec | Single-center | General inpatient | 2012-2017 | ICD-9, 10 | Post-neurosurgical sepsis | Cancer history | In-hospital | 105 | 61.5 ± 15.5 | 44.8 | 30.5 |
| Rygard (2017) [78] | Denmark, Sweden, Norway, Finland | RCT | Multinational | 32 ICUs | 2011-2013 | Sepsis-1 | Septic shock | Hema or meta | 28/90-day | 998 | 66.1 ± 11.9 | — | 16.3 |
| Ryoo (2015) [79] | South Korea | Cohort | Single-center | ED-based | 2010-2012 | Sepsis-3 | Septic shock | Cancer | 28-day | 426 | 62.9 ± 13.4 | 39.0 | 55.9 |
| Sakr (2006) [80] | 24 European countries | Cohort | Multinational | 98 ICUs | 2002 | Sepsis-2 | Septic shock | Cancer, hema | 60-day | 462 | 63.0 ± 16.0 | 38.5 | 13.2 |
| Sakr (2014) [81] | Germany | Cohort | Single-center | Surgical ICU | 2004-2010 | Sepsis-1 | Post-surgery sepsis | Cancer | In-hospital | 1,040 | 67.0 ± 16.0 | 32.6 | 27.7 |
| Sartelli (2019) [82] | 56 countries | Cohort | Global collaborative | 153 surgical units | 2018 | Sepsis-2 | Peritonitis-associated sepsis | Cancer | In-hospital | 3,137 | 47.0 ± 28.2 | 42.1 | 10.6 |
| Schramm (2011) [83] | USA | Cohort | Single-center | ICU | 2007-2009 | Sepsis-2 | Severe sepsis and septic shock | Hema, meta | In-hospital | 962 | 66.8 ± 15.9 | 44.4 | 18.6 |
| Schumer (1976) [84] | USA | Cohort | Single-center | General inpatient | 1967-1976 | Culture and blood pressure | Septic shock | Cancer | In-hospital | 500 | 54 (20-86) | 6.4 | 14.6 |
| Sharma (2021) [85] | USA | Cohort | Nationwide | General inpatient | 2008-2017 | ICD-9/10 | Sepsis | Cancer, hema, solid | In-hospital | 19,160,733 | 65.4 ±17.4 | 50.2 | 20.4 |
| Shi (2025) [86] | China | Cohort | Single-center | ICU | 2020-2022 | Sepsis-3 | Sepsis | Cancer | In-hospital | 2,389 | 66.3 ± 20.0 | 35.2 | 11.3 |
| Su (2018) [87] | Taiwan | Cohort | Single-center | ED-based | 2007-2013 | Sepsis-2 | Parkinson’s disease with sepsis | Cancer | 28-day | 1,200 | 77.3 ± 8.3 | 48.1 | 9.5 |
| Sundén-C. (2005) [88] | Sweden | Cohort | Single-center | General inpatient | 1998-2001 | Sepsis-1 | Sepsis | Cancer history | 28-day | 64 | — | 37.5 | 32.8 |
| Tai (2020) [89] | China | Cohort | Single-center | ICU | 2015-2018 | Sepsis-2 | Peritonitis-associated sepsis | Cancer | 28-day | 246 | 69 (52-82) | 37.4 | 11.0 |
| Thibaud (2024) [90] | France | Cohort | Single-center | ICU | 2008-2019 | Sepsis-3 | Biliary sepsis | Cancer, solid, hema, meta | 90-day | 122 | 67.7 ± 13.3 | 45.1 | 63.1 |
| Tolsma (2014) [91] | France | Cohort | 11 units | ICU | 1997-2011 | Main diagnosis | Severe sepsis and septic shock | Solid, hema | 28-day | 1,981 | 64.7 ± 17.0 | 41.0 | 14.1 |
| Wang (2002) [92] | Taiwan | Cohort | Single-center | General inpatient | 1994-1999 | Sepsis-1 | Community Acinetobacter Sepsis | Cancer, solid, hema | 90-day | 19 | 61.5 ± 14.4 | 26.3 | 52.6 |
| Wang (2012) [93] | China | Cohort | — | General inpatient | 2010-2011 | Sepsis-1 | Sepsis | Cancer | 28-day | 214 | 60.2 ± 18.9 | 36.0 | 10.3 |
| Wang (2014) [94] | USA | Cohort | Population-based | General inpatient | 2003-2007 | Sepsis-2 | Community-acquired sepsis | Cancer history | 6-year | 970 | 68.2 ± 10.3 | 47.6 | 13.5 |
| Wang (2020a) [95] | China | Cohort | 16 hospitals | ICU | 2014-2015 | Sepsis-2 | Sepsis | Cancer | ICU | 2,086 | 62.5 ± 17.8 | 34.7 | 9.9 |
| Wang (2020b) [96] | Taiwan | Cohort | Single-center | ICU | 2015 | Sepsis-2 | Severe sepsis | Cancer, meta or hema | In-hospital | 132 | 79.7 ± 9.6 | 41.7 | 22.0 |
| Wang (2021a) [97] | China | Cohort | Single-center | General inpatient | 2010-2020 | Sepsis-3 | Sepsis-induced blood pressure drop | Cancer history | 30-day | 717 | 69.0 ± 17.0 | — | 17.9 |
| Wang (2021b) [98] | China | Cohort | Regional-based | General inpatient | 2005-2017 | ICD-10 | Sepsis-associated AKI | Cancer | In-hospital | 19,359 | 75.3 ± 14.8 | 37.2 | 19.2 |
| Wu (2011) [99] | Taiwan | Cohort | Single-center | ICU | 2008-2009 | Sepsis-2 | Severe sepsis with organ dysfunction | Active cancer | 28-day | 35 | 73.1 ± 3.2 | 31.4 | 22.9 |
| Xie (2020) [100] | China | Cross-sec | 44 hospitals | ICU | 2015-2016 | Sepsis-1 | Sepsis | Solid, hema | 90-day | 2,322 | 60.8 ± 18.4 | 35.4 | 14.1 |
| Yin (2021) [101] | China | Cohort | Single-center | ICU | 2016-2018 | Sepsis-3 | Sepsis | Cancer | In-hospital | 174 | 64.9 ± 19.7 | 37.4 | 9.8 |
| Zhang (2024a) [102] | China | Cohort | Single-center | General inpatient | 2010-2022 | Sepsis-3 | Post-ERCP  Enterobacterales sepsis | Cancer | 30-day | 195 | 71.1 ± 12.0 | 30.8 | 32.8 |
| Zhang (2024b) [103] | China | Cohort | Single-center | ICU  ED-based | 2013-2017 | Sepsis-3 | Sepsis-associated NTIS | Cancer history | 30-day | 328 | 72.6 ± 15.4 | 39.9 | 5.5 |
| Zhou (2014) [104] | China | Cohort | 22 units | ICU | 2009 | Sepsis-1 | Severe sepsis and septic shock | Cancer, hema | In-hospital | 484 | 63.0 ± 19.3 | 30.6 | 11.4 |
| Zhou (2021) [105] | China | Cohort | Regional-based | General inpatient | 2019 | Sepsis-3 | Septic shock | Cancer | In-hospital | 3,643 | 74.7 ± 17.0 | 35.6 | 19.4 |

Continuous variables are expressed as mean ± SD unless otherwise specified. Where applicable, medians with IQRs or ranges were converted to estimated means and SDs using established methods [106]. APACHE II, Acute Physiology and Chronic Health Evaluation; AKI, acute kidney injury; Aus/NZ, Australian and New Zealand; CRRT, continuous renal replacement therapy; ED, emergency department; ERCP, endoscopic retrograde cholangiopancreatography; HDU, high dependency unit; hema, hematological cancer; ICD, International Classification of Diseases; ICU, intensive care unit; meta, metastatic cancer; NSQIP, National Surgical Quality Improvement Program; NTIS, non-thyroidal illness syndrome; RCT, randomized controlled trial; T2DM, type 2 diabetes mellitus; VAP, ventilator-associated pneumonia.

**Table S4-2.** Baseline characteristics of supplementary shared cohort studies.

| **Study** | **Country/ Territory** | **Study design** | **Study scope** | **Enrollment setting** | **Study year** | **Sepsis definition** | **Clinical population** | **Cancer type** | **Mortality endpoint** | **Sample size** | **Age** | **Female**  **(%)** | **Cancer**  **(%)** |
| --- | --- | --- | --- | --- | --- | --- | --- | --- | --- | --- | --- | --- | --- |
| Garnacho M. (2006) [107] | Spain | Cohort | Single-center | ICU | 2002-2004 | Sepsis-1 | Septic shock | Cancer | In-hospital | 114 | 61.2 ± 18.0 | 53.5 | 6.1 |
| Jouffroy (2021) [108] | France | Cohort | Single-center | ICU | 2017-2019 | 2005 French intensive association definition | Septic shock | Cancer history | In-hospital,  30/90-day | 406 | 71.0 ± 15.0 | 34.0 | 27.8 |
| Lai (2023) [109] | USA | Cohort | Single-center | ICU | 2008-2019 | Sepsis-3 | Sepsis-associated AKI | Cancer | 28-day | 5,056 | 68.6 ± 15.2 | 47.5 | 15.3 |
| Li (2024a) [110] | USA | Cohort | Single-center | ICU | 2008-2022 | Sepsis-3 | Sepsis with T2DM | Cancer, meta | 28-day | 1,489 | 76.5 ± 8.7 | 42.8 | 13.8 |
| Li (2024b) [111] | USA | Cohort | Single-center | ICU | 2008-2019 | Sepsis-3 | Sepsis-associated AKI (renal replacement) | Cancer | In-hospital | 1,663 | 65.0 ± 14.8 | 39.6 | 11.4 |
| Liu (2022) [112] | USA | Cohort | Single-center | ICU | 2008-2019 | Sepsis-3 | Sepsis induced liver injury | Cancer | In-hospital | 770 | 66.8 ± 14.5 | 42.1 | 15.8 |
| Ma (2025) [113] | USA | Cohort | Single-center | ICU | 2008-2019 | Sepsis-3 | Sepsis | Cancer | 28/90-day | 18,198 | 68.7 ± 12.6 | 43.5 | 13.3 |
| Mu (2025) [114] | USA | Cohort | Single-center | ICU | 2001-2012 | Sepsis-3 | Sepsis induced ARDS | Meta | In-hospital | 1,727 | 62.0 ± 17.3 | 36.9 | 1.6 |
| Peng (2021) [115] | USA | Cohort | Single-center | ICU | 2001-2012 | Sepsis-3 | Sepsis | Cancer | 1-year | 2,784 | 67.3 ± 26.2 | 45.5 | 22.6 |
| Peng (2022) [116] | USA | Cohort | Single-center | ICU | 2008-2019 | Sepsis-3 | SAE | Cancer, meta | 30-day | 6,994 | 70.8 ± 16.6 | 45.1 | 16.9 |
| Weng (2021) [117] | USA | Cohort | Nationwide | ICU | 2014-2015 | Sepsis-3 | Sepsis | Hema, meta | ICU | 4,236 | 68.7 ± 17.0 | 46.8 | 5.7 |
| Zhang (2025) [118] | USA | Cohort | Single-center | ICU | 2008-2022 | Sepsis-3 | Septic shock | Cancer | 30-day | 4,942 | 66.5 ± 16.0 | 43.0 | 13.8 |

Continuous variables are expressed as mean ± SD unless otherwise specified. Where applicable, medians with IQRs or ranges were converted to estimated means and SDs using established methods [106]. These studies were not included in the primary overall analysis when overlapping with a more appropriate primary cohort but were used for endpoint-specific, subgroup, or sensitivity analyses where relevant. AKI, acute kidney injury; ARDS, acute respiratory distress syndrome; hema, hematological cancer; ICU, intensive care unit; meta, metastatic cancer; SAE, sepsis-associated encephalopathy; T2DM, type 2 diabetes mellitus.

**Table S4-3.** Mortality outcomes in primary studies.

| **Study** | **Endpoint** | **Cancer**  **(died/survived)** | **Non-cancer (died/survived)** | **Reported effect^a^**  **(95% CI)** | **Calculated crude RR (95% CI)** | **Calculated crude OR (95% CI)** | **Analysis use^b^** | | |
| --- | --- | --- | --- | --- | --- | --- | --- | --- | --- |
|  |  |  |  |  |  |  | **Primary** | **Sensitivity^c^** | **Subgroup^d^** |
| Acar (2024) [1] | 30-day | 37/24 | 63/121 | — | 1.77 (1.33-2.35) | 2.96 (1.63-5.38) | Early | RR | — |
| Acar (2024) [1] | 30-day | 2/8 | 63/121 | — | 0.58 (0.17-2.05) | 0.48 (0.10-2.33) | — | — | Solid |
| Acar (2024) [1] | 30-day | 35/16 | 63/121 | — | 2.00 (1.53-2.63) | 4.20 (2.16-8.17) | — | — | Hema |
| Ahlström (2022) [2] | 60-day | 973/1284 | 6658/13439 | aOR 1.67 (1.52-1.85) | 1.30 (1.24-1.37) | 1.53 (1.40-1.67) | Intermediate | RR | — |
| Ahlström (2022) [2] | 60-day | 505/481 | 6658/13439 | aOR 1.85 (1.58-2.17) | 1.55(1.45-1.65) | 2.12 (1.86-2.41) | — | — | Hema |
| Al Omar (2024) [3] | 30-day | — | — | aOR 3.61 (1.07-12.13) | — | — | — | — | — |
| Artero (2010) [4] | In-hospital | 7/3 | 40/62 | OR 3.61 (0.88-14.80) | 1.79 (1.11-2.86) | 3.62 (0.88-14.81) | In-hospital | RR | — |
| Beck (2014) [5] | In-hospital | 755/396 | 2697/2666 | — | 1.30 (1.24-1.37) | 1.88 (1.65-2.15) | In-hospital | RR | Septic shock |
| Beck (2014) [5] | In-hospital | 390/195 | 2697/2666 | aOR 1.88 (1.46-2.41) | 1.33 (1.24-1.41) | 1.98 (1.65-2.37) | — | — | Hema |
| Beck (2014) [5] | In-hospital | 365/201 | 2697/2666 | aOR 1.63 (1.32-2.01) | 1.28 (1.20-1.37) | 1.80 (1.50-2.15) | — | — | Meta |
| Boonmee (2020) [6] | In-hospital | 151/216 | 305/944 | — | 1.68 (1.44-1.97) | 2.16 (1.69-2.76) | In-hospital | RR | — |
| Burnham (2015) [7] | 30-day | 43/226 | 24/217 | — | 1.61 (1.01-2.56) | 1.72 (1.01-2.93) | Early | RR | — |
| Burnham (2015) [7] | 30-day | 32/112 | 24/217 | aOR 2.92 (2.14-3.97) | 2.23 (1.37-3.63) | 2.58 (1.45-4.60) | — | — | Solid |
| Burnham (2015) [7] | 30-day | 11/114 | 24/217 | — | 0.88 (0.45-1.74) | 0.87 (0.41-1.84) | — | — | Hema |
| Busani (2017) [8] | 30-day | 16/7 | 24/47 | aOR 2.97 (1.14-7.71) | 2.06 (1.35-3.14) | 4.48 (1.62-12.35) | Early | aOR, RR | Septic shock |
| Camou (2020) [9] | ICU | 100/152 | 64/119 | — | 1.13 (0.88-1.46) | 1.22 (0.82-1.82) | ICU | RR | Septic shock |
| Camou (2020) [9] | 28-day | 121/131 | 68/115 | — | 1.29 (1.03-1.62) | 1.56 (1.06-2.30) | Early | RR | Septic shock |
| Camou (2020) [9] | 180-day | 150/102 | 75/108 | — | 1.45 (1.19-1.78) | 2.12 (1.44-3.12) | Extended | RR | Not pooled  (septic shock, <3 estimates) |
| Camou (2020) [9] | ICU | 60/73 | 64/119 | — | 1.29 (0.98-1.69) | 1.53 (0.97-2.41) | — | — | Solid |
| Camou (2020) [9] | 28-day | 74/59 | 68/115 | — | 1.50 (1.18-1.91) | 2.12 (1.35-3.34) | — | — | Solid |
| Camou (2020) [9] | 180-day | 90/43 | 75/108 | aHR 1.16 (0.70-1.93) | 1.65 (1.34-2.04) | 3.01 (1.89-4.81) | — | — | Not pooled  (Solid, <3 estimates) |
| Camou (2020) [9] | ICU | 40/79 | 64/119 | — | 0.96 (0.70-1.32) | 0.94 (0.58-1.53) | — | — | Hema |
| Camou (2020) [9] | 28-day | 47/72 | 68/115 | — | 1.06 (0.79-1.42) | 1.10 (0.69-1.77) | — | — | Hema |
| Camou (2020) [9] | 180-day | 60/59 | 75/108 | aHR 0.9 (0.68-1.21) | 1.23 (0.96-1.58) | 1.46 (0.92-2.33) | — | — | Not pooled  (Hema, <3 estimates) |
| Cebeci (2025) [10] | 28-day | 76/41 | 235/94 | — | 0.91 (0.78-1.06) | 0.74 (0.47-1.16) | — | — | Solid |
| Chebl (2021) [11] | In-hospital | 99/273 | 120/447 | — | 1.26 (1.00-1.59) | 1.35 (0.99-1.83) | In-hospital | RR | — |
| Chen (2014) [12] | In-hospital | 26/6 | 114/108 | aOR 7.24 (2.25-23.31) | 1.58 (1.28-1.95) | 4.11 (1.63-10.36) | In-hospital | aOR, RR | — |
| Chen (2022) [13] | 1-year | — | — | HR 1.22 (1.00-1.48) | — | — | Not pooled  (no raw data) | HR | — |
| Cheng (2007) [14] | In-hospital | 28/10 | 127/153 | aOR 4.60 (1.83-11.55) | 1.62 (1.29-2.04) | 3.37 (1.58-7.21) | In-hospital | aOR, RR | — |
| Cooper (2020) [15] | In-hospital | 2130/5359 | 2959/10527 | — | 1.30 (1.24-1.36) | 1.41 (1.33-1.51) | In-hospital | RR | — |
| Cooper (2020) [15] | In-hospital | 1305/3318 | 2959/10527 | — | 1.29 (1.22-1.36) | 1.40 (1.30-1.51) | — | — | Solid |
| Cooper (2020) [15] | In-hospital | 825/2041 | 2959/10527 | — | 1.31 (1.23-1.40) | 1.44(1.31-1.57) | — | — | Hema |
| Fabbri (2019) [16] | In-hospital | — | — | OR 1.48 (1.17-1.88) | — | 1.48 (1.17-1.88) | In-hospital | — | — |
| Fang (2017) [17] | 14-day | 37/58 | 81/356 | — | 2.10 (1.53-2.89) | 2.80 (1.74-4.52) | Descriptive only | — | — |
| Fang (2017) [17] | 28-day | 48/47 | 110/327 | — | 2.01 (1.55-2.59) | 3.04 (1.92-4.79) | Early | RR | — |
| Fang (2017) [17] | 90-day | 63/32 | 160/277 | — | 1.81 (1.50-2.19) | 3.41 (2.13-5.44) | Intermediate | RR | — |
| Firempong (2014) [18] | In-hospital | 86/63 | 179/229 | OR 1.75 (1.20-2.55); aOR 2.10 (1.38-3.19) | 1.32 (1.10-1.57) | 1.75 (1.20-2.55) | In-hospital | aOR, RR | Septic shock |
| Gameiro (2018) [19] | In-hospital | 50/59 | 103/244 | HR 1.42 (1.14-1.77); aHR 1.20 (0.93-1.55) | 1.55 (1.19-2.01) | 2.01 (1.29-3.12) | In-hospital | aHR, RR | — |
| García G.-R. (2024) [20] | 28-day | 10/38 | 16/109 | OR 1.79 (0.75-4.29) | 1.63 (0.79-3.33) | 1.79 (0.75-4.29) | Early | RR | — |
| Garnacho M. (2020) [21] | In-hospital | 6/3 | 8/38 | OR 0.17 (0.05-0.59)  for survival | 3.83 (1.76-8.37) | 9.50 (1.95-46.21) | In-hospital | RR | — |
| Gonzalez (2016) [22] | ICU | 12/23 | 66/122 | — | 0.98 (0.59-1.61) | 0.96 (0.45-2.06) | ICU | RR | Septic shock |
| Gonzalez (2016) [22] | ICU | 9/1 | 66/122 | — | 2.56 (1.93-3.40) | 16.64 (2.06-134.18) | — | — | Hema |
| Hall (2015) [23] | In-hospital | 4/13 | 8/35 | aRR 1.16 (0.30-4.44) | 1.26 (0.44-3.65) | 1.35 (0.35-5.24) | In-hospital | RR | — |
| He (2023) [24] | In-hospital | 104/49 | 314/363 | aOR 2.80 (1.68-4.67) | 1.47 (1.28-1.68) | 2.45 (1.69-3.56) | In-hospital | aOR, RR | — |
| Holmbom (2022) [25] | 30-day | 9/41 | 29/203 | — | 1.44 (0.73-2.85) | 1.54 (0.68-3.49) | Early | RR | — |
| Hsieh (2021) [26] | In-hospital | — | — | OR 3.29 (3.14-3.44); aOR 3.23 (3.08-3.39) | — | 3.29 (3.14-3.44) | In-hospital | aOR | — |
| Huang (2019a) [27] | In-hospital | 30/31 | 59/232 | aOR 3.28 (1.78-6.07) | 2.43 (1.72-3.41) | 3.81 (2.14-6.78) | In-hospital | aOR, RR | — |
| Huang (2019b) [28] | 28-day | — | — | OR 1.63 (1.09-2.46); aOR 3.45 (1.23-10.0) | — | 1.63 (1.09-2.46) | Early | aOR | Septic shock |
| Inchai (2015) [29] | 30-day | 52/25 | 224/320 | HR 2.14 (1.60-2.90); aHR 1.60 (1.02-2.42) | 1.64 (1.36-1.97) | 2.97 (1.79-4.93) | Early | RR, HR | — |
| Jamme (2017) [30] | 3-day | 50/178 | 60/436 | — | 1.81 (1.29-2.55) | 2.04 (1.35-3.09) | Descriptive only | — | — |
| Jamme (2017) [30] | ICU | 109/119 | 163/333 | — | 1.45 (1.21-1.75) | 1.87 (1.36-2.58) | ICU | RR | Septic shock |
| Jamme (2017) [30] | In-hospital | 119/109 | 179/317 | — | 1.45 (1.22-1.72) | 1.93 (1.41-2.66) | In-hospital | RR | Septic shock |
| Jamme (2017) [30] | 3-day | 30/92 | 60/436 | — | 2.03 (1.37-3.01) | 2.37 (1.45-3.88) | Descriptive only | — | — |
| Jamme (2017) [30] | ICU | 67/55 | 163/333 | — | 1.67 (1.36-2.05) | 2.49 (1.66-3.72) | — | — | Solid |
| Jamme (2017) [30] | In-hospital | 72/50 | 179/317 | aHR 2.20(1.64-2.96) | 1.64 (1.35-1.97) | 2.55 (1.70-3.82) | — | — | Solid |
| Jamme (2017) [30] | 3-day | 20/86 | 60/436 | — | 1.56 (0.98-2.47) | 1.69 (0.97-2.95) | Descriptive only | — | — |
| Jamme (2017) [30] | ICU | 42/64 | 163/333 | — | 1.21 (0.92-1.57) | 1.34 (0.87-2.07) | — | — | Hema |
| Jamme (2017) [30] | In-hospital | 47/59 | 179/317 | aHR 1.02(0.69-1.51) | 1.23 (0.96-1.57) | 1.41 (0.92-2.16) | — | — | Hema |
| Jouffroy (2023) [31] | 30-day | 70/116 | 94/250 | — | 1.38 (1.07-1.77) | 1.60 (1.10-2.35) | Early | RR | Septic shock |
| Kang (2024) [32] | In-hospital | 38196/76366 | 65735/193242 | OR 1.47 (1.45-1.49) | 1.31 (1.30-1.33) | 1.47 (1.45-1.49) | In-hospital | RR | — |
| Kang (2024) [32] | In-hospital | 33717/67603 | 65735/193242 | OR 1.44 (1.41-1.46) aOR 1.83 (1.79-1.87) | 1.31 (1.30-1.33) | 1.47 (1.44-1.49) | — | — | Solid |
| Kang (2024) [32] | In-hospital | 5650/11261 | 65735/193242 | OR 1.32 (1.28-1.36); aOR 2.10 (2.01-2.18) | 1.32 (1.29-1.35) | 1.47 (1.43-1.52) | — | — | Hema |
| Kang (2024) [32] | In-hospital | 7277/11661 | 65735/193242 | OR 1.67 (1.62-1.72); aOR 2.22 (2.05-2.32) | 1.51 (1.49-1.54) | 1.83 (1.78-1.89) | — | — | Meta |
| Kattouf (2022) [33] | In-hospital | 10/8 | 557/1207 | — | 1.76 (1.16-2.67) | 2.71 (1.06-6.90) | — | — | Meta |
| Khwannimit (2009) [34] | In-hospital | 52/24 | 142/172 | — | 1.51 (1.24-1.84) | 2.62 (1.54-4.47) | In-hospital | RR | — |
| Khwannimit (2009) [34] | In-hospital | 37/23 | 142/172 | — | 1.36 (1.08-1.72) | 1.95 (1.11-3.43) | — | — | Hema |
| Khwannimit (2009) [34] | In-hospital | 15/1 | 142/172 | — | 2.07 (1.74-2.47) | 18.17 (2.37-139.23) | — | — | Meta |
| Kikuchi (2024) [35] | ICU | 38/142 | 65/251 | — | 1.03 (0.72-1.47) | 1.03 (0.66-1.62) | ICU | RR | — |
| Kikuchi (2024) [35] | ICU | 20/113 | 65/251 | aOR 1.07 (0.61-1.84) | 0.73 (0.46-1.16) | 0.68 (0.40-1.18) | — | — | Solid |
| Kikuchi (2024) [35] | ICU | 18/29 | 65/251 | aOR 2.48 (1.19-5.07) | 1.86 (1.22-2.84) | 2.40 (1.25-4.58) | — | — | Hema |
| Kim (2018) [36] | 28-day | 9/22 | 5/36 | — | 2.38 (0.89-6.40) | 2.95 (0.87-9.93) | Early | RR | Septic shock |
| Kim (2018) [36] | 28-day | 8/19 | 5/36 | — | 2.43 (0.89-6.65) | 3.03 (0.87-10.56) | — | — | Solid |
| Kim (2018) [36] | 28-day | 1/3 | 5/36 | — | 2.05 (0.31-13.51) | 2.40 (0.21-27.78) | — | — | Hema |
| Kim (2018) [36] | 28-day | 3/4 | 5/36 | OR 4.75 (0.78-27.53) | 3.51 (1.07-11.50) | 5.40 (0.92-31.55) | — | — | Meta |
| Kim (2022) [37] | 28-day | 95/75 | 222/257 | — | 1.21 (1.02-1.42) | 1.47 (1.03-2.09) | Early | RR | — |
| Kim (2022) [37] | 28-day | 74/55 | 222/257 | HR 1.30 (1.00-1.68); aHR 1.20 (0.91-1.57) | 1.24 (1.04-1.48) | 1.56 (1.05-2.31) | — | — | Solid |
| Kim (2022) [37] | 28-day | 21/20 | 222/257 | HR 1.10 (0.71-1.71) | 1.11 (0.81-1.51) | 1.22 (0.64-2.30) | — | — | Hema |
| Koçak (2025) [38] | 28-day | 19/28 | 95/105 | — | 0.85 (0.58-1.24) | 0.75 (0.39-1.43) | — | — | Solid |
| Korkmaz (2017) [39] | ICU | 13/5 | 47/49 | — | 1.48 (1.04-2.10) | 2.71 (0.90-8.19) | ICU | RR | — |
| Kung (2012) [40] | In-hospital | 4/10 | 7/46 | OR 0.38 (0.09-1.55)  for survival | 2.16 (0.74-6.36) | 2.63 (0.64-10.72) | In-hospital | RR | — |
| Kuperman (2013) [41] | In-hospital | 57/299 | 72/364 | OR 0.96 (0.66-1.41); aOR 1.03 (0.64-1.65) | 0.97 (0.71-1.33) | 0.96 (0.66-1.41) | In-hospital | aOR, RR | — |
| Le Borgne (2022) [42] | 28-day | 38/110 | 27/64 | — | 0.87 (0.57-1.32) | 0.82 (0.46-1.46) | Early | RR | Septic shock |
| Le Borgne (2022) [42] | ICU | 45/103 | 27/64 | aOR 1.41 (0.65-3.04) | 1.02 (0.69-1.53) | 1.04 (0.59-1.83) | ICU | RR | Septic shock |
| Le Borgne (2022) [42] | In-hospital | 59/89 | 32/59 | aOR 1.99 (0.98-4.03) | 1.13 (0.81-1.60) | 1.22 (0.71-2.10) | In-hospital | aOR, RR | Septic shock |
| Le Borgne (2022) [42] | 90-day | 51/70 | 33/56 | — | 1.14 (0.81-1.60) | 1.24 (0.71-2.17) | Intermediate | RR | Septic shock |
| Le Borgne (2022) [42] | 180-day | 61/60 | 37/52 | — | 1.21 (0.90-1.64) | 1.43 (0.82-2.48) | Extended | RR | Not pooled  (septic shock, <3 estimates) |
| Le Borgne (2022) [42] | 1-year | 69/52 | 40/49 | — | 1.27 (0.96-1.67) | 1.63 (0.94-2.82) | Not pooled  (overlapped estimate) | — | Not pooled  (septic shock, overlapped estimate) |
| Le Borgne (2022) [42] | ICU | — | — | aOR 1.48 (0.62-3.51) | — | — | — | — | Not pooled  (solid, no raw data) |
| Le Borgne (2022) [42] | In-hospital | — | — | aOR 2.52 (1.12-5.67) | — | — | — | — | Not pooled  (solid, no raw data) |
| Le Borgne (2022) [42] | ICU | — | — | aOR 1.33 (0.53-3.32) | — | — | — | — | Not pooled  (hema, no raw data) |
| Le Borgne (2022) [42] | In-hospital | — | — | aOR 1.62 (0.67-3.87) | — | — | — | — | Not pooled  (hema, no raw data) |
| Lee (2013) [43] | 28-day | 23/79 | 28/271 | — | 2.41 (1.46-3.98) | 2.82 (1.54-5.16) | Early | RR | — |
| Lee (2013) [43] | 28-day | 21/49 | 28/271 | OR 4.30 (2.28-8.11); aOR 4.39 (2.01-9.60) | 3.20 (1.94-5.29) | 4.15 (2.18-7.89) | — | — | Meta |
| Lee (2025) [44] | ICU | 20/136 | 49/202 | — | 0.66 (0.41-1.06) | 0.61 (0.35-1.07) | ICU | RR | — |
| Lee (2025) [44] | In-hospital | 65/91 | 95/156 | — | 1.10 (0.86-1.41) | 1.17 (0.78-1.76) | In-hospital | RR | — |
| Lee (2025) [44] | In-hospital | — | — | HR 1.02 (0.72-1.43) | — | — | — | — | Not pooled  (solid, no raw data) |
| Lee (2025) [44] | In-hospital | — | — | HR 1.74 (1.00-3.02); aHR 1.49 (0.74-3.02) | — | — | — | — | Not pooled  (hema, no raw data) |
| Lee (2025) [44] | ICU | — | — | HR 0.67 (0.37-1.23) | — | — | — | — | Not pooled  (solid, no raw data) |
| Lee (2025) [44] | ICU | — | — | HR 3.62 (1.71-7.67); aHR 2.68 (1.26-5.70) | — | — | — | — | Not pooled  (hema, no raw data) |
| Li (2021) [45] | 28-day | 5/8 | 74/187 | — | 1.36 (0.66-2.77) | 1.58 (0.50-4.98) | Early | RR | — |
| Lin (2022) [46] | In-hospital | — | — | HR 2.03 (1.51-2.73); aHR 2.23 (1.59-3.11) | — | — | Not pooled  (no raw data) | aHR | — |
| Liu (2025) [47] | In-hospital | 172/378 | 563/1861 | — | 1.35 (1.17-1.55) | 1.50 (1.23-1.84) | In-hospital | RR | — |
| López (2021) [48] | 90-day | 22/58 | 16/155 | aHR 2.69 (1.40-5.16) | 2.94 (1.63-5.28) | 3.67 (1.80-7.48) | Intermediate | RR | — |
| López M. (2018) [49] | 28-day | 36/31 | 53/76 | — | 1.31 (0.97-1.77) | 1.67 (0.92-3.02) | Early | RR | — |
| M. Lessa (2024) [50] | 28-day | 104/61 | 286/202 | — | 1.08 (0.94-1.24) | 1.20 (0.84-1.73) | Early | RR | — |
| M. Lessa (2024) [50] | 28-day | 29/9 | 286/202 | — | 1.30 (1.07-1.58) | 2.28 (1.05-4.91) | — | — | Hema |
| Macphail (2024) [51] | ICU | 3537/13776 | 29846/235468 | — | 1.82 (1.76-1.87) | 2.03 (1.95-2.11) | — | — | Hema |
| Macphail (2024) [51] | In-hospital | 5405/11908 | 45218/220096 | OR 2.26 (2.17-2.36); aOR 2.29 (2.02-2.60) | 1.83 (1.79-1.88) | 2.21 (2.14-2.29) | — | — | Hema |
| Maia (2023) [52] | In-hospital | 31/132 | 69/632 | aOR 1.89 (0.88-4.36) | 1.93 (1.31-2.85) | 2.15 (1.35-3.42) | In-hospital | aOR, RR | — |
| Man (2022) [53] | ICU | 59/204 | 49/210 | — | 1.19 (0.85-1.66) | 1.24 (0.81-1.90) | ICU | RR | — |
| Man (2022) [53] | 30-day | 90/173 | 63/196 | — | 1.41 (1.07-1.85) | 1.62 (1.11-2.37) | Early | RR | — |
| Man (2022) [53] | In-hospital | 108/155 | 71/188 | — | 1.50 (1.17-1.91) | 1.84 (1.28-2.66) | In-hospital | RR | — |
| Man (2022) [53] | 1-year | 164/99 | 95/164 | — | 1.70 (1.41-2.05) | 2.86 (2.00-4.08) | Extended | RR | — |
| Man (2022) [53] | 30-day | 30/46 | 63/196 | — | 1.62 (1.14-2.31) | 2.03 (1.18-3.48) | — | — | Hema |
| Matos (2025) [54] | In-hospital | 1864/4281 | 7656/45187 | — | 2.09 (2.01-2.19) | 2.57 (2.42-2.73) | In-hospital | RR | — |
| Mazzone (2016) [55] | In-hospital | 25/91 | 53/364 | aOR 2.14 (1.16-3.94) | 1.70 (1.10-2.60) | 1.89 (1.11-3.20) | — | — | Solid |
| Musat (2025) [56] | In-hospital | 946/612 | 5570/4961 | OR 1.38 (1.24-1.53); aOR 1.51 (1.37-1.61) | 1.15 (1.10-1.20) | 1.38 (1.24-1.53) | In-hospital | aOR, RR | — |
| Musat (2025) [56] | In-hospital | 661/431 | 5570/4961 | OR 1.35 (1.19-1.53) aOR 1.57 (1.40-1.76) | 1.14 (1.09-1.20) | 1.37 (1.20-1.55) | — | — | Solid |
| Musat (2025) [56] | In-hospital | 285/181 | 5570/4961 | OR 1.36 (1.13-1.65); aOR 1.54 (1.26-1.87) | 1.16 (1.07-1.25) | 1.40 (1.16-1.70) | — | — | Hema |
| Nam (2019) [57] | 28-day | 2/8 | 21/94 | — | 1.10 (0.30-4.01) | 1.12 (0.22-5.66) | Early | RR | — |
| N. Panday (2020) [58] | In-hospital | 33/324 | 126/2175 | — | 1.69 (1.17-2.44) | 1.76 (1.18-2.62) | In-hospital | RR | — |
| N. Panday (2020) [58] | 28-day | 54/303 | 162/2139 | — | 2.15 (1.61-2.86) | 2.35 (1.69-3.28) | Early | RR | — |
| N. Panday (2020) [58] | 90-day | 82/275 | 234/2067 | — | 2.26 (1.80-2.83) | 2.63 (1.99-3.49) | Intermediate | RR | — |
| N. Panday (2020) [58] | In-hospital | 7/11 | 21/60 | — | 1.50 (0.76-2.98) | 1.82 (0.62-5.30) | — | — | Septic shock |
| N. Panday (2020) [58] | 28-day | 9/9 | 19/62 | — | 2.13 (1.16-3.91) | 3.26 (1.13-9.39) | — | — | Septic shock |
| N. Panday (2020) [58] | 90-day | 9/9 | 25/56 | — | 1.62 (0.92-2.85) | 2.24 (0.79-6.32) | — | — | Septic shock |
| N. Panday (2020) [58] | In-hospital | 20/222 | 126/2175 | — | 1.51 (0.96-2.37) | 1.56 (0.95-2.54) | — | — | Solid |
| N. Panday (2020) [58] | 28-day | 33/209 | 162/2139 | — | 1.94 (1.36-2.75) | 2.08 (1.40-3.11) | — | — | Solid |
| N. Panday (2020) [58] | 90-day | 53/189 | 234/2067 | — | 2.15 (1.65-2.81) | 2.48 (1.77-3.46) | — | — | Solid |
| N. Panday (2020) [58] | In-hospital | 6/34 | 126/2175 | — | 2.74 (1.28-5.84) | 3.05 (1.26-7.39) | — | — | Hema |
| N. Panday (2020) [58] | 28-day | 11/29 | 162/2139 | — | 3.91 (2.31-6.60) | 5.01 (2.46-10.21) | — | — | Hema |
| N. Panday (2020) [58] | 90-day | 12/28 | 234/2067 | — | 2.95 (1.81-4.81) | 3.79 (1.90-7.54) | — | — | Hema |
| Nielsen (2025) [59] | 2-year | 75/25 | 279/335 | HR 2.16 (1.67–2.78); aHR 1.91 (1.44–2.53) | 1.65 (1.43-1.90) | 3.60 (2.23-5.82) | Extended | aHR, HR, RR | — |
| Nygård (2014) [60] | In-hospital | 13/18 | 42/147 | OR 2.53 (1.15-5.58); aOR 5.50 (1.92-15.78) | 1.89 (1.15-3.09) | 2.53 (1.15-5.58) | In-hospital | aOR, RR | — |
| Oliveira (2002) [61] | In-hospital | 2/4 | 12/11 | — | 0.64 (0.19-2.12) | 0.46 (0.07-3.02) | In-hospital | RR | — |
| Oliveros (2024) [62] | 1-year | — | — | aHR 2.07 (1.92-2.23) | — | — | Not pooled  (no raw data) | aHR | — |
| Oliveros (2024) [62] | 1-year | — | — | aHR 2.03 (1.92-2.15) | — | — | — | — | — |
| Orak (2018) [63] | In-hospital | 34/7 | 185/104 | — | 1.30 (1.10-1.53) | 2.73 (1.17-6.38) | In-hospital | RR | — |
| Orsetti (2019) [64] | 30-day | 13/2 | 30/11 | — | 1.18 (0.90-1.55) | 2.38 (0.46-12.30) | — | — | Solid |
| Pansirit. (2022) [65] | 28-day | 18/17 | 88/221 | HR 1.95 (1.17-3.24) | 1.81 (1.25-2.61) | 2.66 (1.31-5.39) | Early | RR, HR | — |
| Park (2012) [66] | In-hospital | — | — | aOR 1.89 (1.13-3.17) | — | — | Not pooled  (no raw data) | aOR | — |
| Paulsen (2015) [67] | 30-day | 26/70 | 76/201 | aOR 0.97 (0.57-1.66) | 0.99 (0.68-1.44) | 0.98 (0.58-1.66) | Early | aOR, RR | — |
| Pittet (1993) [68] | In-hospital | 12/5 | 63/93 | aRR 2.02 (0.96-4.28) | 1.75 (1.22-2.51) | 3.54 (1.19-10.55) | In-hospital | RR | — |
| Proaño-Z. (2023) [69] | 30-day | 57/32 | 621/629 | aOR 2.61 (1.55-4.38) | 1.29 (1.09-1.52) | 1.80 (1.15-2.82) | — | — | Meta |
| Qu (2021) [70] | 28-day | — | — | HR 1.55(1.14-2.11); aHR 1.40 (1.02-1.91) | — | — | Not pooled  (no raw data) | HR | — |
| Rannikko (2017) [71] | 90-day | 43/59 | 55/340 | — | 3.03 (2.17-4.23) | 4.51 (2.77-7.32) | Intermediate | RR | — |
| Rannikko (2017) [71] | 90-day | 15/30 | 55/340 | — | 2.39 (1.48-3.87) | 3.09 (1.56-6.11) | — | — | Hema |
| Rannikko (2017) [71] | 90-day | 28/29 | 55/340 | — | 3.53 (2.46-5.06) | 5.97 (3.30-10.79) |  |  | Not pooled  (meta, <3 estimates) |
| Ravetti (2015) [72] | ICU | 16/24 | 10/25 | — | 1.40 (0.73-2.67) | 1.67 (0.63-4.39) | ICU | RR | — |
| Ravetti (2015) [72] | 28-day | 18/22 | 12/23 | — | 1.31 (0.74-2.33) | 1.57 (0.62-4.00) | Early | RR | — |
| Reaven (2022) [73] | 72-hrs | 87/413 | 191/1723 | OR 1.90 (1.45-2.51); aOR 1.53 (1.11-2.11) | 1.74 (1.38-2.20) | 1.90 (1.44-2.51) | Descriptive only | — | — |
| Riché (2009) [74] | 30-day | 12/33 | 23/112 | aRR 1.54 (0.62-3.83) | 1.57 (0.85-2.88) | 1.77 (0.80-3.94) | Early | RR | — |
| Ripa (2017) [75] | 7-day | — | — | OR 1.48 (0.87-2.50) | — | — | Descriptive only | — | Not pooled  (hema, <3 estimates) |
| Roh (2019) [76] | 180-day | 43/32 | 138/233 | OR 2.27 (1.37-3.75) aOR 2.28 (1.32-3.93) | 1.54 (1.22-1.95) | 2.27 (1.37-3.75) | — | — | Not pooled  (hema, <3 estimates) |
| Roh (2019) [76] | 1-year | 53/22 | 160/211 | OR 3.18 (1.86-5.44) aOR 3.23 (1.83-5.70) | 1.64 (1.36-1.98) | 3.18 (1.86-5.44) | — | — | Not pooled  (overlapped estimate) |
| Rotter (2020) [77] | In-hospital | 15/17 | 17/56 | OR 3.09 (1.27-7.61) aOR 14.69 (3.06-105.20) | 2.01 (1.15-3.51) | 2.91 (1.20-7.02) | In-hospital | aOR, RR | — |
| Rygard (2017) [78] | 90-day | 54/41 | 385/518 | — | 1.33 (1.10-1.61) | 1.77 (1.16-2.72) | — | — | Not pooled  (Hema/meta) |
| Ryoo (2015) [79] | 28-day | 51/187 | 35/153 | OR 1.19 (0.74-1.93) | 1.15 (0.78-1.69) | 1.19 (0.74-1.93) | Early | RR | Septic shock |
| Sakr (2006) [80] | 60-day | 38/23 | 181/220 | — | 1.38 (1.10-1.73) | 2.01 (1.15-3.49) | Intermediate | RR | Septic shock |
| Sakr (2006) [80] | 60-day | 18/5 | 181/220 | — | 1.73 (1.36-2.21) | 4.38 (1.59-12.02) | — | — | Hema |
| Sakr (2014) [81] | In-hospital | — | — | OR 2.35 (1.76-3.06); aOR 2.12 (1.50-2.99) |  | 2.35 (1.76-3.06) | In-hospital | aOR |  |
| Sartelli (2019) [82] | In-hospital | 83/250 | 197/2607 | aOR 3.02 (2.15-4.24) | 3.55 (2.82-4.46) | 4.39 (3.30-5.86) | In-hospital | aOR, RR | — |
| Schramm (2011) [83] | In-hospital | 55/124 | 197/586 | — | 1.22 (0.95-1.57) | 1.32 (0.92-1.88) | In-hospital | RR | — |
| Schramm (2011) [83] | In-hospital | 42/84 | 197/586 | aOR (leuke) 1.68 (1.08-2.61) (lymph) 1.49 (1.44-5.01) | 1.32 (1.01-1.74) | 1.49 (0.99-2.23) | — | — | Hema |
| Schramm (2011) [83] | In-hospital | 13/40 | 197/586 | aOR 1.10 (0.56-2.13) | 0.97 (0.60-1.59) | 0.97 (0.51-1.84) | — | — | Meta |
| Schumer (1976) [84] | In-hospital | 19/54 | 115/312 | — | 0.97 (0.64-1.47) | 0.95 (0.54-1.68) | In-hospital | RR | Septic shock |
| Sharma (2021) [85] | In-hospital | 699719/3214093 | 1852501/13394420 | aOR: 18-44, 3.4 (3.24-3.57); 45-64, 1.99 (1.95-2.03); 65-84, 1.27 (1.26-1.29); >=85, 0.93 (0.91-0.95); | 1.47 (1.47-1.48) | 1.57 (1.57-1.58) | In-hospital | aOR, RR | — |
| Sharma (2021) [85] | In-hospital | 543019/2577779 | 1852501/13394420 | — | 1.43 (1.43-1.44) | 1.52 (1.52-1.53) | — | — | Solid |
| Sharma (2021) [85] | In-hospital | 156700/636314 | 1852501/13394420 | — | 1.63 (1.62-1.63) | 1.78 (1.77-1.79) | — | — | Hema |
| Shi (2025) [86] | In-hospital | — | — | aHR 1.65 (1.26-2.16) | — | — | Not pooled  (no raw data) | aHR | — |
| Su (2018) [87] | 28-day | 16/98 | 70/1016 | aOR 2.80 (1.47-5.54) | 2.18 (1.31-3.62) | 2.37 (1.32-4.24) | Early | aOR, RR |  |
| Sundén-C. (2005) [88] | 28-day | 5/16 | 5/38 | — | 2.05 (0.67-6.30) | 2.38 (0.60-9.35) | Early | RR | — |
| Tai (2020) [89] | 28-day | 7/20 | 52/167 | — | 1.09 (0.55-2.16) | 1.12 (0.45-2.81) | Early | RR | — |
| Thibaud (2024) [90] | 90-day | 32/45 | 5/40 | HR 4.33 (1.69-11.11) | 3.74 (1.57-8.91) | 5.69 (2.02-16.00) | Intermediate | RR | — |
| Thibaud (2024) [90] | 90-day | 30/42 | 5/40 | HR 3.47 (1.524–7.903) | 3.75 (1.57-8.95) | 5.71 (2.02-16.18) | — | — | Solid |
| Thibaud (2024) [90] | 90-day | 2/3 | 5/40 | HR 1.24 (0.30–5.2) | 3.60 (0.93-13.95) | 5.33 (0.71-40.06) | — | — | Hema |
| Thibaud (2024) [90] | 90-day | 13/12 | 5/40 | HR 2.57 (1.30-5.04); aHR 3.50 (1.51-8.10) | 4.68 (1.89-11.61) | 8.67 (2.57-29.25) | — | — | Not pooled  (meta, <3 estimates) |
| Tolsma (2014) [91] | 28-day | — | — | aHR 1.41 (1.00-1.99) | — | — | — | — | — |
| Tolsma (2014) [91] | 28-day | — | — | aHR 1.81 (1.25-2.62) | — | — | — | — | — |
| Wang (2002) [92] | 90-day | 4/6 | 7/2 | — | 0.51 (0.22-1.19) | 0.19 (0.03-1.43) | Intermediate | RR | — |
| Wang (2012) [93] | 28-day | 13/9 | 84/108 | — | 1.35 (0.92-1.98) | 1.86 (0.76-4.55) | Early | RR | — |
| Wang (2014) [94] | 6 years | — | — | HR 1.68 (1.24-2.27); aHR 1.21 (1.09-1.34) | — | — | Not pooled  (no raw data) | aHR, HR | — |
| Wang (2020a) [95] | ICU | — | — | aOR 2.43 (1.39-4.23) | — | — | Not pooled  (no raw data) | aOR | — |
| Wang (2020a) [95] | ICU | 61/80 | 381/612 | — | 1.13 (0.92-1.38) | 1.22 (0.86-1.75) | — | — | Septic shock |
| Wang (2020b) [96] | In-hospital | 13/16 | 25/78 | aOR 0.63 (0.15-2.72) | 1.85 (1.09-3.13) | 2.53 (1.07-5.99) | In-hospital | aOR, RR | — |
| Wang (2020b) [96] | In-hospital | 11/5 | 25/78 | aOR 10.05 (1.27-79.74) | 2.83 (1.76-4.55) | 6.86 (2.18-21.66) | — | — | Not pooled  (Hema/meta) |
| Wang (2020b) [96] | In-hospital | 2/11 | 25/78 | — | 0.63 (0.17-2.37) | 0.57 (0.12-2.73) | — | — | Solid |
| Wang (2021a) [97] | 30-day | 44/84 | 93/496 | aOR 3.92 (2.36-6.55) | 2.18 (1.61-2.95) | 2.79 (1.82-4.28) | Early | RR | — |
| Wang (2021b) [98] | In-hospital | 2430/1288 | 8217/7424 | aOR 2.03 (1.86-2.23) | 1.24 (1.21-1.28) | 1.70 (1.58-1.84) | In-hospital | aOR, RR | — |
| Wu (2011) [99] | 28-day | 5/3 | 7/20 | — | 2.41 (1.05-5.55) | 4.76 (0.90-25.30) | Early | RR | — |
| Xie (2020) [100] | 90-day | 147/181 | 677/1317 | — | 1.32 (1.15-1.51) | 1.58 (1.25-2.00) | Intermediate | RR | — |
| Xie (2020) [100] | 90-day | 112/174 | 677/1317 | — | 1.15 (0.99-1.35) | 1.25 (0.97-1.62) | — | — | Solid |
| Xie (2020) [100] | 90-day | 35/7 | 677/1317 | aOR 4.23(1.73-10.34) | 2.45 (2.12-2.85) | 9.73 (4.30-22.01) | — | — | Hema |
| Yin (2021) [101] | In-hospital | 6/11 | 44/113 | — | 1.26 (0.63-2.51) | 1.40 (0.49-4.02) | In-hospital | RR | — |
| Zhang (2024a) [102] | 30-day | 31/33 | 61/70 | aOR 0.90 (0.50-1.70) | 1.04 (0.76-1.42) | 1.08 (0.59-1.96) | Early | aOR, RR | — |
| Zhang (2024b) [103] | 30-day | 5/13 | 46/264 | — | 1.87 (0.85-4.13) | 2.21 (0.75-6.49) | Early | RR | — |
| Zhou (2014) [104] | In-hospital | 28/27 | 134/295 | OR 2.28 (1.30-4.02); aOR 2.25 (1.14-4.42) | 1.63 (1.21-2.19) | 2.28 (1.30-4.02) | In-hospital | aOR, RR | — |
| Zhou (2014) [104] | In-hospital | 3/7 | 134/295 | — | 0.96 (0.37-2.50) | 0.94 (0.24-3.71) | — | — | Hema |
| Zhou (2021) [105] | In-hospital | 416/291 | 1461/1475 | OR 0.69 (0.59-0.82);  aOR 0.64 (0.54-0.76) for survival | 1.18 (1.10-1.27) | 1.44 (1.22-1.70) | In-hospital | aOR, RR | Septic shock |

^a^ All crude ORs and RRs in this table use non-cancer patients as the comparator when raw event data were available. For studies reporting multiple cancer subgroups with a shared non-cancer control group, subgroup data were combined for the overall cancer versus non-cancer estimate when appropriate. Recalculated crude estimates may differ from originally reported effects because of differences in comparator group, adjustment status, or available raw event counts. Cancer subgroup estimates were calculated using non-cancer patients as the reference group whenever data were available. ^b^ The “Analysis use” column indicates how each estimate contributed to the review. ^c^ Sensitivity labels indicate estimate-level use, including adjusted OR, RR-based, or HR-derived. Study-level sensitivity analyses, such as exclusion of ICD-based studies, exclusion by sample size, RVE analyses, and leave-one-out analyses, were conducted using the relevant primary dataset. ^d^ Estimates included in the primary endpoint analyses also contributed to applicable study-level subgroup analyses; these subgroup results are not shown in “Subgroup” column. CI, confidence interval; HR, hazard ratio; hema, hematological cancer; ICU, intensive care unit; meta, metastatic cancer; NA, not applicable; OR, odds ratio; RR, relative risk.

**Table S4-4.** Mortality outcomes supplementary analyses of shared cohorts.

| **Study** | **Cohort origin** | **Mortality endpoint** | **Clinical enrollment scope** | **Cancer**  **(died/survived)** | **Comparator (died/survived)** | **Reported effect**  **(95% CI)** | **Calculated crude RR (95% CI)** | **Calculated crude OR (95% CI)** | **Analysis use** |
| --- | --- | --- | --- | --- | --- | --- | --- | --- | --- |
| Garnacho M. (2006) [107] | Hospital Virgen | In-hospital | Septic shock | 6/1 | 43/64 | RR 8.90 (1.01-417) | 2.13 (1.46-3.12) | 8.93 (1.04-76.82) | Subgroup: Septic shock |
| Jouffroy (2021) [108] | Prehospital EMS | 90-day | Septic shock | 57/78 | 78/193 | — | 1.47 (1.12-1.93) | 1.81 (1.18-2.78) | Subgroup: Septic shock |
| Lai (2023) [109] | MIMIC-IV | 28-day | Sepsis-associated AKI | NA | NA | HR 1.58 (1.35-1.83); aHR 1.87 (1.60-2.19) | — | — | Not pooled  (<3 estimates) |
| Li (2024a) [110] | MIMIC-IV | 28-day | Sepsis with T2DM | 56/150 | 277/1006 | — | 1.26 (0.98-1.61) | 1.36 (0.97-1.89) | Not pooled  (<3 estimates) |
| Li (2024b) [111] | MIMIC-IV | In-hospital | AKI (renal replacement) | 102/88 | 657/816 | — | 1.20 (1.04-1.39) | 1.44 (1.06-1.95) | Not pooled  (<3 estimates) |
| Liu (2022) [112] | MIMIC-IV | In-hospital | Sepsis induced liver injury | 66/56 | 269/379 | — | 1.30 (1.08-1.57) | 1.66 (1.13-2.45) | Not pooled  (<3 estimates) |
| Ma (2025) [113] | MIMIC-IV | 28-day | Sepsis 28-day mortality | 692/1735 | 2584/13187 | — | 1.74 (1.62-1.87) | 2.04 (1.85-2.24) | Primary: early |
| Ma (2025) [113] | MIMIC-IV | 90-day | Sepsis 90-day mortality | 1002/1425 | 3573/12198 | — | 1.82 (1.72-1.93) | 2.40 (2.20-2.62) | Primary: intermediate  Sensitivity: RR |
| Mu (2025) [114] | MIMIC-III | In-hospital | Sepsis induced ARDS | 26/1 | 741/959 | — | 2.21 (2.02-2.42) | 33.65 (4.56-248.54) | Not pooled  (<3 estimates) |
| Peng (2021) [115] | MIMIC-III | 1-year | Sepsis 1 year | NA | NA | HR 1.86 (1.47-2.36); aHR 1.69 (1.31-2.17) | — | — | Not pooled  (no raw data) |
| Peng (2022) [116] | MIMIC-IV | 30-day | SAE | 342/839 | 890/4923 | — | 1.89 (1.70-2.11) | 2.25 (1.95-2.61) | Not pooled  (<3 estimates) |
| Weng (2021) [117] | eICU-CRD | ICU | Metastatic cancer subgroup | NA | NA | aOR 1.80 (1.05-3.00) | — | — | Not pooled  (no raw data) |
| Zhang (2025) [118] | MIMIC-IV | 30-day | Septic shock | 290/390 | 1356/2906 | — | 1.34 (1.22-1.48) | 1.59 (1.35-1.88) | Subgroup: Septic shock |

AKI, acute kidney injury; ARDS, acute respiratory distress syndrome; CRD, Collaborative Research database; CI, confidence interval; HR, hazard ratio; meta, metastatic cancer; ICU, intensive care unit; MIMIC, Medical Information Mart for Intensive Care; NA, not applicable; OR, odds ratio; SAE, sepsis-associated encephalopathy; T2DM, type 2 diabetes mellitus; RR, relative risk.

**Supplementary Material 5.** Risk of bias assessment

**Table S5-1.** Cohort studies (n = 99)

| **Study** | **Total Stars** | **Quality**  **tier** | **Selection** | | | | **Comparability** | | **Outcome** | | |
| --- | --- | --- | --- | --- | --- | --- | --- | --- | --- | --- | --- |
|  |  |  | **Representativeness** | **Selection** | **Ascertainment of exposure** | **Outcome not at Start** | **Comparability** | **Critical factors** | **Assessment** | **Follow-up** | **Adequacy** |
| Acar (2024) [1] | 6 | Moderate | 0 | 1 | 1 | 0 | 1 | 0 | 1 | 1 | 1 |
| Ahlström (2022) [2] | 7 | High | 1 | 1 | 1 | 0 | 1 | 0 | 1 | 1 | 1 |
| Al Omar (2024) [3] | 6 | Moderate | 1 | 1 | 1 | 0 | 0 | 0 | 1 | 1 | 1 |
| Artero (2010) [4] | 4 | Moderate | 0 | 1 | 0 | 0 | 0 | 0 | 1 | 1 | 1 |
| Beck (2014) [5] | 7 | High | 1 | 1 | 1 | 0 | 1 | 0 | 1 | 1 | 1 |
| Boonmee (2020) [6] | 5 | Moderate | 0 | 1 | 0 | 0 | 1 | 0 | 1 | 1 | 1 |
| Burnham (2015) [7] | 6 | Moderate | 0 | 1 | 1 | 0 | 1 | 0 | 1 | 1 | 1 |
| Busani (2017) [8] | 5 | Moderate | 0 | 1 | 0 | 0 | 1 | 0 | 1 | 1 | 1 |
| Camou (2020) [9] | 9 | High | 1 | 1 | 1 | 1 | 1 | 1 | 1 | 1 | 1 |
| Cebeci (2025) [10] | 5 | Moderate | 0 | 1 | 1 | 0 | 0 | 0 | 1 | 1 | 1 |
| Chebl (2021) [11] | 4 | Moderate | 0 | 1 | 0 | 0 | 0 | 0 | 1 | 1 | 1 |
| Chen (2014) [12] | 6 | Moderate | 0 | 1 | 1 | 0 | 1 | 0 | 1 | 1 | 1 |
| Chen (2022) [13] | 3 | Low | 0 | 1 | 0 | 0 | 0 | 0 | 1 | 0 | 1 |
| Cheng (2007) [14] | 6 | Moderate | 1 | 1 | 0 | 0 | 1 | 0 | 1 | 1 | 1 |
| Cooper (2020) [15] | 7 | High | 1 | 1 | 1 | 1 | 0 | 0 | 1 | 1 | 1 |
| Fabbri (2019) [16] | 4 | Moderate | 0 | 1 | 0 | 0 | 0 | 0 | 1 | 1 | 1 |
| Fang (2017) [17] | 7 | High | 1 | 1 | 1 | 1 | 0 | 0 | 1 | 1 | 1 |
| Firempong (2014) [18] | 7 | High | 1 | 1 | 1 | 0 | 1 | 0 | 1 | 1 | 1 |
| Gameiro (2018) [19] | 5 | Moderate | 0 | 1 | 0 | 0 | 1 | 0 | 1 | 1 | 1 |
| García G.-R. (2024) [20] | 5 | Moderate | 0 | 1 | 1 | 0 | 0 | 0 | 1 | 1 | 1 |
| Garnacho M. (2020) [21] | 4 | Moderate | 0 | 1 | 0 | 0 | 0 | 0 | 1 | 1 | 1 |
| Gonzalez (2016) [22] | 5 | Moderate | 0 | 1 | 1 | 0 | 0 | 0 | 1 | 1 | 1 |
| He (2023) [24] | 4 | Moderate | 0 | 1 | 0 | 0 | 1 | 0 | 1 | 1 | 0 |
| Holmbom (2022) [25] | 5 | Moderate | 1 | 1 | 0 | 0 | 0 | 0 | 1 | 1 | 1 |
| Hsieh (2021) [26] | 6 | Moderate | 1 | 1 | 0 | 0 | 1 | 0 | 1 | 1 | 1 |
| Huang (2019a) [27] | 5 | Moderate | 0 | 1 | 0 | 0 | 1 | 0 | 1 | 1 | 1 |
| Huang (2019b) [28] | 5 | Moderate | 0 | 1 | 0 | 0 | 1 | 0 | 1 | 1 | 1 |
| Inchai (2015) [29] | 5 | Moderate | 0 | 1 | 0 | 0 | 1 | 0 | 1 | 1 | 1 |
| Jamme (2017) [30] | 9 | High | 1 | 1 | 1 | 1 | 1 | 1 | 1 | 1 | 1 |
| Jouffroy (2023) [31] | 5 | Moderate | 1 | 1 | 0 | 0 | 0 | 0 | 1 | 1 | 1 |
| Kang (2024) [32] | 7 | High | 1 | 1 | 1 | 0 | 1 | 0 | 1 | 1 | 1 |
| Kattouf (2022) [33] | 6 | Moderate | 1 | 1 | 1 | 0 | 0 | 0 | 1 | 1 | 1 |
| Khwannimit (2009) [34] | 5 | Moderate | 0 | 1 | 1 | 0 | 0 | 0 | 1 | 1 | 1 |
| Kikuchi (2024) [35] | 6 | Moderate | 0 | 1 | 1 | 0 | 1 | 0 | 1 | 1 | 1 |
| Kim (2018) [36] | 5 | Moderate | 0 | 1 | 1 | 0 | 1 | 0 | 1 | 1 | 0 |
| Kim (2022) [37] | 6 | Moderate | 1 | 1 | 0 | 0 | 1 | 0 | 1 | 1 | 1 |
| Koçak (2025) [38] | 5 | Moderate | 0 | 1 | 1 | 0 | 0 | 0 | 1 | 1 | 1 |
| Korkmaz (2017) [39] | 5 | Moderate | 0 | 1 | 1 | 0 | 0 | 0 | 1 | 1 | 1 |
| Kung (2012) [40] | 4 | Moderate | 0 | 1 | 0 | 0 | 0 | 0 | 1 | 1 | 1 |
| Kuperman (2013) [41] | 5 | Moderate | 0 | 1 | 0 | 0 | 1 | 0 | 1 | 1 | 1 |
| Le Borgne (2022) [42] | 9 | High | 1 | 1 | 1 | 1 | 1 | 1 | 1 | 1 | 1 |
| Lee (2013) [43] | 6 | Moderate | 0 | 1 | 1 | 0 | 1 | 0 | 1 | 1 | 1 |
| Lee (2025) [44] | 7 | High | 1 | 1 | 1 | 0 | 1 | 0 | 1 | 1 | 1 |
| Li (2021) [45] | 5 | Moderate | 0 | 1 | 1 | 0 | 0 | 0 | 1 | 1 | 1 |
| Lin (2022) [46] | 4 | Moderate | 0 | 1 | 0 | 0 | 1 | 0 | 1 | 1 | 0 |
| Liu (2025) [47] | 4 | Moderate | 1 | 1 | 0 | 0 | 0 | 0 | 1 | 1 | 0 |
| López (2021) [48] | 9 | High | 1 | 1 | 1 | 1 | 1 | 1 | 1 | 1 | 1 |
| López M. (2018) [49] | 4 | Moderate | 0 | 1 | 0 | 0 | 0 | 0 | 1 | 1 | 1 |
| M. Lessa (2024) [50] | 5 | Moderate | 0 | 1 | 0 | 0 | 1 | 0 | 1 | 1 | 1 |
| Macphail (2024) [51] | 9 | High | 1 | 1 | 1 | 1 | 1 | 1 | 1 | 1 | 1 |
| Maia (2023) [52] | 5 | Moderate | 0 | 1 | 0 | 0 | 1 | 0 | 1 | 1 | 1 |
| Man (2022) [53] | 9 | High | 1 | 1 | 1 | 1 | 1 | 1 | 1 | 1 | 1 |
| Matos (2025) [54] | 6 | Moderate | 1 | 1 | 1 | 1 | 0 | 0 | 1 | 1 | 0 |
| Mazzone (2016) [55] | 7 | High | 1 | 1 | 1 | 0 | 1 | 0 | 1 | 1 | 1 |
| Musat (2025) [56] | 6 | Moderate | 0 | 1 | 1 | 0 | 1 | 0 | 1 | 1 | 1 |
| Nam (2019) [57] | 5 | Moderate | 0 | 1 | 1 | 0 | 0 | 0 | 1 | 1 | 1 |
| Nielsen (2025) [59] | 4 | Moderate | 0 | 1 | 0 | 0 | 1 | 0 | 1 | 0 | 1 |
| Nygård (2014) [60] | 5 | Moderate | 0 | 1 | 0 | 0 | 1 | 0 | 1 | 1 | 1 |
| Oliveros (2024) [62] | 6 | Moderate | 1 | 1 | 1 | 0 | 1 | 0 | 1 | 0 | 1 |
| Orak (2018) [63] | 3 | Low | 0 | 1 | 0 | 0 | 0 | 0 | 1 | 1 | 0 |
| Orsetti (2019) [64] | 5 | Moderate | 1 | 1 | 1 |  | 0 | 0 | 1 | 1 | 0 |
| Pansirit. (2022) [65] | 4 | Moderate | 0 | 1 | 0 | 0 | 0 | 0 | 1 | 1 | 1 |
| Park (2012) [66] | 6 | Moderate | 1 | 1 | 0 | 0 | 1 | 0 | 1 | 1 | 1 |
| Paulsen (2015) [67] | 6 | Moderate | 1 | 1 | 0 | 0 | 1 | 0 | 1 | 1 | 1 |
| Pittet (1993) [68] | 4 | Moderate | 0 | 1 | 0 | 0 | 0 | 0 | 1 | 1 | 1 |
| Proaño-Z. (2023) [69] | 7 | High | 1 | 1 | 1 | 0 | 1 | 0 | 1 | 1 | 1 |
| Qu (2021) [70] | 6 | Moderate | 1 | 1 | 0 | 0 | 1 | 0 | 1 | 1 | 1 |
| Rannikko (2017) [71] | 5 | Moderate | 0 | 1 | 1 | 0 | 0 | 0 | 1 | 1 | 1 |
| Ravetti (2015) [72] | 9 | High | 1 | 1 | 1 | 1 | 1 | 1 | 1 | 1 | 1 |
| Reaven (2022) [73] | 5 | Moderate | 1 | 1 | 0 | 0 | 1 | 0 | 1 | 0 | 1 |
| Riché (2009) [74] | 4 | Moderate | 0 | 1 | 0 | 0 | 0 | 0 | 1 | 1 | 1 |
| Ripa (2017) [75] | 5 | Moderate | 0 | 1 | 1 | 0 | 0 | 0 | 1 | 1 | 1 |
| Roh (2019) [76] | 6 | Moderate | 0 | 1 | 1 | 0 | 1 | 0 | 1 | 1 | 1 |
| Ryoo (2015) [79] | 4 | Moderate | 0 | 1 | 0 | 0 | 0 | 0 | 1 | 1 | 1 |
| Sakr (2006) [80] | 6 | Moderate | 1 | 1 | 1 | 0 | 0 | 0 | 1 | 1 | 1 |
| Sakr (2014) [81] | 5 | Moderate | 0 | 1 | 0 | 0 | 1 | 0 | 1 | 1 | 1 |
| Sartelli (2019) [82] | 6 | Moderate | 1 | 1 | 0 | 0 | 1 | 0 | 1 | 1 | 1 |
| Schramm (2011) [83] | 6 | Moderate | 0 | 1 | 1 | 0 | 1 | 0 | 1 | 1 | 1 |
| Schumer (1976) [84] | 3 | Low | 0 | 1 | 0 | 0 | 0 | 0 | 1 | 1 | 0 |
| Sharma (2021) [85] | 9 | High | 1 | 1 | 1 | 1 | 1 | 1 | 1 | 1 | 1 |
| Shi (2025) [86] | 5 | Moderate | 0 | 1 | 0 | 0 | 1 | 0 | 1 | 1 | 1 |
| Su (2018) [87] | 6 | Moderate | 0 | 1 | 1 | 0 | 1 | 0 | 1 | 1 | 1 |
| Sundén-C. (2005) [88] | 4 | Moderate | 0 | 1 | 0 | 0 | 0 | 0 | 1 | 1 | 1 |
| Tai (2020) [89] | 4 | Moderate | 0 | 1 | 0 | 0 | 0 | 0 | 1 | 1 | 1 |
| Thibaud (2024) [90] | 6 | Moderate | 0 | 1 | 1 | 0 | 1 | 0 | 1 | 1 | 1 |
| Tolsma (2014) [91] | 9 | High | 1 | 1 | 1 | 1 | 1 | 1 | 1 | 1 | 1 |
| Wang (2002) [92] | 5 | Moderate | 0 | 1 | 1 | 0 | 0 | 0 | 1 | 1 | 1 |
| Wang (2012) [93] | 2 | Low | 0 | 0 | 0 | 0 | 0 | 0 | 1 | 1 | 0 |
| Wang (2014) [94] | 5 | Moderate | 1 | 1 | 0 | 0 | 1 | 0 | 1 | 0 | 1 |
| Wang (2020a) [95] | 6 | Moderate | 1 | 1 | 0 | 0 | 1 | 0 | 1 | 1 | 1 |
| Wang (2020b) [96] | 6 | Moderate | 0 | 1 | 1 | 0 | 1 | 0 | 1 | 1 | 1 |
| Wang (2021a) [97] | 5 | Moderate | 0 | 1 | 1 | 0 | 1 | 0 | 1 | 1 | 0 |
| Wang (2021b) [98] | 6 | Moderate | 1 | 1 | 0 | 0 | 1 | 0 | 1 | 1 | 1 |
| Wu (2011) [99] | 5 | Moderate | 0 | 1 | 1 | 0 | 0 | 0 | 1 | 1 | 1 |
| Yin (2021) [101] | 5 | Moderate | 0 | 1 | 1 | 0 | 0 | 0 | 1 | 1 | 1 |
| Zhang (2024a) [102] | 3 | Low | 0 | 1 | 0 | 0 | 0 | 0 | 1 | 1 | 0 |
| Zhang (2024b) [103] | 5 | Moderate | 0 | 1 | 1 | 0 | 0 | 0 | 1 | 1 | 1 |
| Zhou (2014) [104] | 7 | High | 1 | 1 | 1 | 0 | 1 | 0 | 1 | 1 | 1 |
| Zhou (2021) [105] | 6 | Moderate | 1 | 1 | 0 | 0 | 1 | 0 | 1 | 1 | 1 |

**Table S5-2.** Cross-sectional studies (n=2)

| **Study** | **Quality tier** | **Total stars** | **Selection** | | | | | **Comparability** | | **Outcome** | | |
| --- | --- | --- | --- | --- | --- | --- | --- | --- | --- | --- | --- | --- |
|  |  |  | **Representativeness** | **Sample size** | **Non-respondents** | **Ascertainment of the exposure** | **Cancer Status** | **Basic adjustment** | **Critical Adjustment** | **Outcome assessment** | **Statistical tests** | **Data presentation** |
| Rotter (2020) [77] | Unsatisfactory | 4 | 0 | 0 | 0 | 1 | 0 | 1 | 0 | 1 | 1 | 0 |
| Xie (2020) [100] | Very Good | 9 | 1 | 1 | 1 | 1 | 1 | 1 | 0 | 1 | 1 | 1 |

**Table S5-3.** Randomized controlled trials (n=4)

| **Study** | **Quality Tier** | **Randomization process** | **Deviations from intended interventions** | **Missing outcome data** | **Measurement of the outcome** | **Selection of the reported result** |
| --- | --- | --- | --- | --- | --- | --- |
| Hall (2015) [23] | Some Concerns | Some Concerns | Low Risk | Low Risk | Low Risk | Some concerns |
| N. Panday (2020) [58] | Some Concerns | Low Risk | Some concerns | Low Risk | Low Risk | Some concerns |
| Oliveira (2002) [61] | Some Concerns | Low Risk | Low Risk | Low Risk | Low Risk | Some concerns |
| Rygard (2017) [78] | Some Concerns | Low Risk | Low Risk | Low Risk | Low Risk | Some concerns |

**Table S5-4.** Included studies in subgroup analysis of shared cohorts (n=12)

| **Study** | **Total Stars** | **Quality Tier** | **Selection** | | | | **Comparability** | | **Outcome** | | |
| --- | --- | --- | --- | --- | --- | --- | --- | --- | --- | --- | --- |
|  |  |  | **Representativeness** | **Selection** | **Ascertainment** | **Outcome not at Start** | **Comparability** | **Critical Factors** | **Assessment** | **Follow-up** | **Adequacy** |
| Garnacho M. (2006) [107] | 5 | Moderate | 0 | 1 | 1 | 0 | 0 | 0 | 1 | 1 | 1 |
| Jouffroy (2021) [108] | 5 | Moderate | 1 | 1 | 0 | 0 | 0 | 0 | 1 | 1 | 1 |
| Lai (2023) [109] | 4 | Moderate | 0 | 1 | 0 | 0 | 1 | 0 | 1 | 1 | 0 |
| Li (2024a) [110] | 6 | Moderate | 0 | 1 | 1 | 0 | 1 | 0 | 1 | 1 | 1 |
| Li (2024b) [111] | 4 | Moderate | 0 | 1 | 0 | 0 | 0 | 0 | 1 | 1 | 1 |
| Liu (2022) [112] | 5 | Moderate | 0 | 1 | 1 | 0 | 0 | 0 | 1 | 1 | 1 |
| Ma (2025) [113] | 5 | Moderate | 0 | 1 | 1 | 0 | 0 | 0 | 1 | 1 | 1 |
| Mu (2025) [114] | 4 | Moderate | 0 | 1 | 1 | 0 | 0 | 0 | 1 | 1 | 0 |
| Peng (2021) [115] | 3 | Low | 0 | 1 | 0 | 0 | 1 | 0 | 1 | 0 | 0 |
| Peng (2022) [116] | 5 | Moderate | 0 | 1 | 1 | 0 | 0 | 0 | 1 | 1 | 1 |
| Weng (2021) [117] | 7 | High | 1 | 1 | 1 | 0 | 1 | 0 | 1 | 1 | 1 |
| Zhang (2025) [118] | 4 | Moderate | 0 | 1 | 0 | 0 | 0 | 0 | 1 | 1 | 1 |

**Supplementary Material 6.** Publication bias assessment.

**Figure 6A.** Doi plots of mortality associations across stratified endpoints

| A. Intensive care unit    | B. In‑hospital    | C. Early    |
| --- | --- | --- |
| D. Intermediate    | E. Extended follow-up    |  |

**Figure 6B.** Funnel plots of mortality associations across stratified endpoints

| A. Intensive care unit    | B. In‑hospital (Egger test p-value=0.09)    | C. Early (Egger test p-value=0.46)    |
| --- | --- | --- |
| D. Intermediate (Egger test p-value=0.51)    | E. Extended follow-up    |  |

**Supplementary Material 7.** Descriptive absolute mortality among studies reporting raw event counts.

| **Endpoint category** | **ICU** | **In-hospital** | **Early**  **(28/30‑day)** | **Intermediate**  **(60/90-day)** | **Extended follow-up (≥180‑day)** |
| --- | --- | --- | --- | --- | --- |
| No. of studies with raw data | 9 | 40 | 33 | 11 | 4 |
| Control mortality, % | 28.2 | 12.5 | 18.5 | 27.8 | 42.4 |
| Cancer mortality, % | 31.2 | 18.5 | 31.8 | 41.5 | 61.1 |
| Absolute risk difference, % | 3.0 | 6.0 | 13.4 | 13.7 | 18.7 |
| Excess deaths per 1,000 | 30 | 60 | 134 | 137 | 187 |

**Supplementary Material 8.** Sensitivity analyses.

| **Endpoint category^a^** | **ICU** | **In-hospital** | **Early**  **(28/30‑day)** | **Intermediate**  **(60/90-day)** | **Extended follow-up (≥180‑day)** |
| --- | --- | --- | --- | --- | --- |
| **Primary and OR-based sensitivity analyses** | | | | | |
| Primary analysis | 1.21 (0.94–1.56; 50.6) | 1.90 (1.69–2.13; 97.2) | 1.81 (1.59–2.06; 43.4) | 2.28 (1.72–3.03; 88.8) | 2.41 (1.70–3.42; 59.5) |
| Cancer as primary exposure only | 1.21 (0.88–1.67; 61.6) | 1.69 (1.39–2.04; 97.5) | 1.75 (1.23–2.48; 67.6) | 2.52 (1.61–3.94; 66.4) | 2.15 (1.49–3.10; 55.0) |
| Robust variance estimation | 1.42 (1.10–1.83; p<0.05) | 1.89 (1.69–2.12; p<0.001) | 1.85 (1.63–2.11; p<0.001) | 2.21 (1.89–2.59; p<0.001) | 2.74 (1.95–3.86; p<0.01) |
| Limited to Sepsis‑1/2/3 cohorts | 1.23 (0.88–1.73; 61.6) | 1.95 (1.72–2.21; 97.3) | 1.93 (1.69–2.21; 33.5) | 2.42 (1.82–3.20; 89.6) | — |
| Sample size >10,000 excluded | 1.21 (0.94–1.56; 50.6) | 1.93 (1.68–2.21; 69.4) | 1.80 (1.56–2.06; 41.2) | 2.43 (1.69–3.48; 77.7) | 2.41 (1.70–3.42; 59.5) |
| **Adjusted OR-based analyses** | | | | | |
| Adjusted ORs | — | 2.16 (1.78–2.63; 98.8) | 2.06 (1.18–3.61; 78.2) | — | — |
| **Alternative effect-measure analyses** | | | | | |
| Unadjusted RRs based | 1.16 (1.00–1.35; 42.5) | 1.48 (1.36–1.61; 96.5) | 1.50 (1.36–1.66; 67.4) | 1.69 (1.34–2.15; 92.3) | 1.56 (1.40–1.73; 34.0) |
| Unadjusted HRs based | — | — | 1.85 (1.47–2.33; 10.4) | 1.63 (1.16–2.28; 84.0) | — |
| Adjusted HRs based | — | 1.62 (1.15–2.29; 76.7) | — | — | 1.67 (1.19–2.36; 97.1)^b^ |

^a^ Values are pooled estimates with 95% confidence intervals. I² is reported for conventional random-effects models, whereas model p values are reported for robust variance estimation models. Odds ratio (OR)-, relative risk (RR)-, and hazard ratio (HR)-based estimates should be interpreted directionally rather than as numerically equivalent.

^b^ HR-based estimates reflect time-to-event associations and were reported across heterogeneous endpoints, including 1-year, 2-year, and 6-year endpoints.

**Supplementary Material 9.** Sepsis-1/2 versus Sepsis-3 subgroup analysis.

| **Mortality endpoint** | **Sepsis-1/2** | **Sepsis-3** | **P for subgroup difference** |
| --- | --- | --- | --- |
| ICU | 1.29 (0.94–1.78; 0.0) | 1.09 (0.58–2.09; 84.6) | 0.64 |
| In-hospital | 2.29 (1.86–2.82; 65.0) | 1.92 (1.60–2.30; 86.8) | 0.21 |
| Early | 1.96 (1.64–2.34; 33.1) | 1.89 (1.49–2.39; 39.9) | 0.81 |
| Intermediate | 2.18 (1.51–3.14; 86.3) | 3.05 (1.91–4.85; 49.6) | 0.27 |

Values are pooled odds ratios (ORs) with 95% confidence intervals and I² values. P values represent tests for subgroup differences between Sepsis-1/2 and Sepsis-3 using random-effects models.

**Supplementary Material 10.** Exploratory univariable meta-regression analyses.

| **Covariate** | **k** | **β (SE)** | **95% CI** | **QM p** | **R² (%)** |
| --- | --- | --- | --- | --- | --- |
| **Study-level** **characteristics** |  |  |  |  |  |
| Study year (centered) | 71 | -0.000 (0.007) | -0.014 to 0.013 | 0.96 | 0.0 |
| Sepsis definition | 73 | — | — | 0.11 | 5.3 |
| Analytic context | 73 | — | — | 0.42 | 0.0 |
| Enrollment setting | 73 | — | — | 0.39 | 0.9 |
| Geographic region | 73 | — | — | 0.24 | 2.5 |
| **Clinical severity indicators** |  |  |  |  |  |
| SOFA score (centered) | 36 | -0.008 (0.025) | -0.057 to 0.042 | 0.76 | 0.0 |
| Septic shock (per 10%) | 32 | -0.000 (0.011) | -0.022 to 0.021 | 0.97 | 0.0 |
| ICU admission (per 10%) | 53 | 0.029 (0.014) | 0.003 to 0.056 | **0.03** | 16.8 |
| Mechanical ventilation (per 10%) | 25 | 0.059 (0.025) | 0.010 to 0.109 | **0.02** | 20.2 |
| Renal replacement therapy (per 10%) | 19 | -0.004 (0.026) | -0.056 to 0.048 | 0.87 | 0.0 |
| **Population characteristics** |  |  |  |  |  |
| Age (centered) | 72 | -0.012 (0.008) | -0.027 to 0.003 | 0.11 | 7.1 |
| Female (%) | 72 | 0.004 (0.006) | -0.008 to 0.016 | 0.54 | 0.0 |
| Cancer proportion (per 10%) | 73 | -0.119 (0.034) | -0.185 to -0.053 | **<0.001** | 21.0 |
| Solid tumor (per 10%) | 14 | -0.044 (0.050) | -0.142 to 0.055 | 0.39 | 0.0 |
| Hematologic malignancy (per 10%) | 20 | 0.047 (0.060) | -0.071 to 0.165 | 0.43 | 0.0 |

β coefficients represent change in log odds ratio. QM p value indicates overall test of moderators. R² represents proportion of between-study heterogeneity explained. If the study year contained multiple years, the median year was used as the reference study year prior to centering. ICU, intensive care unit; SOFA, sequential organ failure assessment.

**Supplementary Material 11.** Exploratory multivariable meta-regression analyses.

| **Model summary** | | | | | | |
| --- | --- | --- | --- | --- | --- | --- |
| **Model** | **Variables included** | | | **k** | **R² (%)** | **QM p** **for model** |
| Main model | Age, cancer proportion (per 10%), sepsis definition | | | 72 | 24.2 | **<0.01** |
| Sensitivity analysis 1 | Main model + septic shock (per 10%) | | | 31 | 4.3 | **0.04** |
| Sensitivity analysis 2 | Main model+ mechanical ventilation (per 10%) | | | 25 | 30.1 | 0.18 |
| Sensitivity analysis 3 | Main model+ ICU admission (per 10%) | | | 52 | 40.7 | **<0.01** |
| Sensitivity analysis 4 | Main model + SOFA | | | 35 | 53.5 | **0.04** |
| Sensitivity analysis 5 | Main model+ renal replacement therapy (per 10%) | | | 19 | 72.0 | **0.05** |
| Main model A1 | Age, cancer proportion (per 10%), enrollment setting | | | 72 | 24.4 | **<0.01** |
| Main model A2 | Age, cancer proportion (per 10%), geographic region | | | 72 | 32.6 | **<0.0001** |
| Main model A3 | Age, cancer proportion (per 10%), analytic context | | | 72 | 22.9 | **<0.01** |
| **Variable** | | **β (SE)** | **95% CI** | **p** | | |
| **Main model** | | | | | | |
| Age (centered) | | -0.011 (0.008) | -0.026 to 0.004 | 0.16 | | |
| Sepsis definition (using other definition group as reference) | | | | | | |
| Sepsis-1 | | 0.347 (0.191) | -0.028 to 0.722 | 0.07 | | |
| Sepsis-2 | | 0.299 (0.176) | -0.045 to 0.644 | 0.09 | | |
| Sepsis-3 | | 0.261 (0.173) | -0.078 to 0.599 | 0.13 | | |
| ICD-code | | 0.182 (0.180) | -0.170 to 0.534 | 0.31 | | |
| Cancer proportion (per 10%) | | -0.096 (0.035) | -0.164 to -0.028 | **<0.01** | | |
| **Sensitivity analysis 1** | | | | | | |
| Septic shock (per 10%) | | -0.000 (0.013) | -0.025 to 0.024 | 0.97 | | |
| Age (centered) | | -0.001 (0.012) | -0.023 to 0.022 | 0.96 | | |
| Sepsis definition (using other definition group as reference) | | | | | | |
| Sepsis-1 | | 0.620 (0.209) | 0.210 to 1.029 | **<0.01** | | |
| Sepsis-2 | | 0.482 (0.219) | 0.053 to 0.911 | **0.03** | | |
| Sepsis-3 | | 0.340 (0.231) | -0.113 to 0.793 | 0.14 | | |
| ICD-code | | 0.420 (0.249) | -0.069 to 0.909 | 0.09 | | |
| Cancer proportion (per 10%) | | -0.044 (0.039) | -0.121 to 0.032 | 0.26 | | |
| **Sensitivity analysis 2** | |  |  |  | | |
| Mechanical ventilation (per 10%) | | 0.045 (0.030) | -0.014 to 0.104 | 0.13 | | |
| Age (centered) | | -0.004 (0.017) | -0.038 to 0.030 | 0.83 | | |
| Sepsis definition (using other definition group as reference) | | | | | | |
| Sepsis-1 | | -0.161 (0.313) | -0.775 to 0.452 | 0.61 | | |
| Sepsis-2 | | 0.029 (0.221) | -0.404 to 0.462 | 0.90 | | |
| Sepsis-3 | | 0.091 (0.203) | -0.307 to 0.489 | 0.65 | | |
| ICD-code | | -0.047 (0.189) | -0.417 to 0.323 | 0.80 | | |
| Cancer proportion (per 10%) | | -0.061 (0.041) | -0.142 to 0.020 | 0.14 | | |
| **Sensitivity analysis 3** | | | | | | |
| ICU admission (per 10%) | | 0.030 (0.017) | -0.003 to 0.063 | 0.08 | | |
| Age (centered) | | 0.005 (0.011) | -0.018 to 0.027 | 0.69 | | |
| Sepsis definition (using other definition group as reference) | | | | | | |
| Sepsis-1 | | 0.190 (0.175) | -0.154 to 0.533 | 0.28 | | |
| Sepsis-2 | | 0.101 (0.172) | -0.236 to 0.439 | 0.56 | | |
| Sepsis-3 | | 0.151 (0.160) | -0.162 to 0.464 | 0.34 | | |
| ICD-code | | 0.068 (0.192) | -0.309 to 0.445 | 0.72 | | |
| Cancer proportion (per 10%) | | -0.109 (0.038) | -0.182 to -0.035 | **<0.01** | | |
| **Sensitivity analysis 4** | | | | | | |
| SOFA (centered) | | -0.007 (0.026) | -0.057 to 0.043 | 0.78 | | |
| Age (centered) | | -0.020 (0.016) | -0.050 to 0.011 | 0.21 | | |
| Sepsis definition (using other definition group as reference) | | | | | | |
| Sepsis-1 | | -0.215 (0.286) | -0.775 to 0.345 | 0.45 | | |
| Sepsis-2 | | -0.183 (0.273) | -0.717 to 0.351 | 0.50 | | |
| Sepsis-3 | | -0.379 (0.272) | -0.912 to 0.155 | 0.16 | | |
| ICD-code | | -0.592 (0.530) | -1.630 to 0.446 | 0.26 | | |
| Cancer proportion (per 10%) | | -0.125 (0.042) | -0.208 to -0.042 | **<0.01** | | |
| **Sensitivity analysis 5** | | | | | | |
| Renal replacement therapy (per 10%) | | 0.014 (0.030) | -0.044 to 0.072 | 0.64 | | |
| Age (centered) | | -0.037 (0.020) | -0.076 to 0.003 | 0.07 | | |
| Sepsis definition (using other definition group as reference) | | | | | | |
| Sepsis-1 | | -0.350 (0.304) | -0.946 to 0.246 | 0.25 | | |
| Sepsis-2 | | -0.183 (0.265) | -0.702 to 0.336 | 0.49 | | |
| Sepsis-3 | | -0.206 (0.270) | -0.734 to 0.322 | 0.45 | | |
| ICD-code | | -0.379 (0.233) | -0.836 to 0.079 | 0.10 | | |
| Cancer proportion (per 10%) | | -0.039 (0.042) | -0.121 to 0.043 | 0.36 | | |
| **Main model A1** | |  |  |  | | |
| Age (centered) | | -0.007 (0.008) | -0.022 to 0.008 | 0.38 | | |
| Enrollment setting (using ED-based group as reference) | | | | | | |
| ICU-restricted | | 0.153 (0.125) | -0.091 to 0.397 | 0.22 | | |
| General inpatient | | -0.001 (0.129) | -0.253 to 0.251 | 1.00 | | |
| Mixed setting | | -0.102 (0.237) | -0.566 to 0.362 | 0.67 | | |
| Cancer proportion (per 10%) | | -0.117 (0.034) | -0.183 to -0.051 | **<0.001** | | |
| **Main model A2** | | | | | | |
| Age (centered) | | -0.019 (0.008) | -0.035 to -0.002 | **0.03** | | |
| Geographic region (using other continent group as reference) | | | | | | |
| Asia | | 0.171 (0.199) | -0.218 to 0.561 | 0.39 | | |
| Europe | | 0.089 (0.207) | -0.317 to 0.495 | 0.67 | | |
| North America | | -0.139 (0.194) | -0.519 to 0.242 | 0.48 | | |
| Cancer proportion (per 10%) | | -0.107 (0.033) | -0.172 to -0.043 | **<0.01** | | |
| **Main model A3** | |  |  |  | | |
| Age (centered) | | -0.010 (0.007) | -0.024 to 0.005 | 0.18 | | |
| Analytic context (using comorbidity group as reference) | | | | | | |
| Cancer sepsis | | 0.015 (0.110) | -0.200 to 0.230 | 0.89 | | |
| Cancer proportion (per 10%) | | -0.115 (0.035) | -0.184 to -0.047 | **<0.001** | | |

ED, emergency department; ICD, International Classification of Diseases; ICU, intensive care unit; SOFA, sequential organ failure assessment.

**Supplementary Material 12.** Cumulative meta-analysis.

**References**

1. Acar C, Kilinçer Bozgül ŞM, Bozkurt D: Evaluation of Factors Associated with Adult Sepsis Prognosis. *Namik Kemal Med J* 2024, 12(2):128-133.
2. Ahlstrom B, Frithiof R, Larsson IM, Strandberg G, Lipcsey M, Hultstrom M: A comparison of impact of comorbidities and demographics on 60-day mortality in ICU patients with COVID-19, sepsis and acute respiratory distress syndrome. *Sci Rep* 2022, 12(1):15703.
3. Al Omar S, Alshraideh JA, Oweidat I, Al Qadire M, Khalaf A, Sumaqa YA, Al-Mugheed K, Alabdullah AAS, Abdelaliem SMF: Mortality of patients with sepsis in intensive care units at tertiary hospitals in Jordan Prospective cohort study. *Medicine (Baltimore)* 2024, 103(43):e40169.
4. Artero A, Zaragoza R, Camarena JJ, Sancho S, González R, Nogueira JM: Prognostic factors of mortality in patients with community-acquired bloodstream infection with severe sepsis and septic shock. *J Crit Care* 2010, 25(2):276-281.
5. Beck V, Chateau D, Bryson GL, Pisipati A, Zanotti S, Parrillo JE, Kumar A, Cooperative Antimicrobial Therapy of Septic Shock Database Research G: Timing of vasopressor initiation and mortality in septic shock: a cohort study. *Crit Care* 2014, 18(3):R97.
6. Boonmee P, Ruangsomboon O, Limsuwat C, Chakorn T: Predictors of Mortality in Elderly and Very Elderly Emergency Patients with Sepsis: A Retrospective Study. *West J Emerg Med* 2020, 21(6):210-218.
7. Burnham JP, Lane MA, Kollef MH: Impact of Sepsis Classification and Multidrug-Resistance Status on Outcome Among Patients Treated With Appropriate Therapy. *Crit Care Med* 2015, 43(8):1580-1586.
8. Busani S, Serafini G, Mantovani E, Venturelli C, Giannella M, Viale P, Mussini C, Cossarizza A, Girardis M: Mortality in Patients With Septic Shock by Multidrug Resistant Bacteria: Risk Factors and Impact of Sepsis Treatments. *J Intensive Care Med* 2019, 34(1):48-54.
9. Camou F, Didier M, Leguay T, Milpied N, Daste A, Ravaud A, Mourissoux G, Guisset O, Issa N: Long-term prognosis of septic shock in cancer patients. *Supportive Care Cancer* 2020, 28(3):1325-1333.
10. Cebeci Y, Bayraktar YŞ: Association between Platelet-to-Lymphocyte Ratio, C-reactive Protein to Albumin Ratio, Red Cell Distribution Width, and APACHE II Score in Predicting Prognosis and Mortality in Sepsis. *Eurasian J Emerg Med* 2025, 24(2):89-94.
11. Chebl RB, Geha M, Assaf M, Kattouf N, Haidar S, Abdeldaem K, Halawi N, Khamis M, Makki M, Tamim H *et al*: The prognostic value of the lactate/albumin ratio for predicting mortality in septic patients presenting to the emergency department: a prospective study. *Ann Med* 2021, 53(1):2268-2277.
12. Chen CM, Cheng KC, Chan KS, Yu WL: Age May Not Influence the Outcome of Patients with Severe Sepsis in Intensive Care Units. *Int J Gerontol* 2014, 8(1):22-26.
13. Chen SH, Wang YC, Chao A, Liu CM, Chiu CT, Wang MJ, Yeh YC: Early prediction of survival at different time intervals in sepsis patients: A visualized prediction model with nomogram and observation study. *Tzu Chi Med J* 2022, 34(1):55-61.
14. Cheng BL, Xie GH, Yao SL, Wu XM, Guo QL, Gu MN, Fang Q, Xu QP, Wang DX, Jin YH *et al*: Epidemiology of severe sepsis in critically ill surgical patients in ten university hospitals in China. *Crit Care Med* 2007, 35(11):2538-2546.
15. Cooper AJ, Keller SP, Chan C, Glotzbecker BE, Klompas M, Baron RM, Rhee C: Improvements in sepsis-associated mortality in hospitalized patients with cancer versus those without cancer: A 12-year analysis using clinical data. *Ann Am Thora Soc* 2020, 17(4):466-473.
16. Fabbri A, Marchesini G, Benazzi B, Morelli A, Montesi D, Bini C, Rizzo SG: Old subjects with sepsis in the emergency department: trend analysis of case fatality rate. *BMC Geriatr* 2019, 19(1).
17. Fang WF, Chen YM, Lin CY, Huang KT, Kao HC, Fang YT, Huang CH, Chang YT, Wang YH, Wang CC *et al*: Immune profiles and clinical outcomes between sepsis patients with or without active cancer requiring admission to intensive care units. *PloS one* 2017, 12(7):e0179749.
18. Firempong AO, Shaheen MA, Pan DY, Drazin D: Racial and ethnic disparities in the incidence and mortality from septic shock and respiratory failure among elective neurosurgery patients. *Neurol Res* 2014, 36(10):857-865.
19. Gameiro J, Gonçalves M, Pereira M, Rodrigues N, Godinho I, Neves M, Gouveia J, e Silva ZC, Jorge S, Lopes JA: Obesity, acute kidney injury and mortality in patients with sepsis: A cohort analysis. *Ren Fail* 2018, 40(1):120-126.
20. García de Guadiana-Romualdo L, Botella LA, Rodríguez Rojas C, Puche Candel A, Jimenez Sánchez R, Conesa Zamora P, Albaladejo-Otón MD, Allegue-Gallego JM: Mortality prediction model from combined serial lactate, procalcitonin and calprotectin levels in critically ill patients with sepsis: A retrospective study according to Sepsis-3 definition. *Med Intensiva* 2024, 48(11):629-638.
21. Garnacho-Montero J, Palacios-Garcia I, Diaz-Martin A, Gutierrez-Pizarraya A, Lopez-Sanchez JM, Gomez EA, Cordero MD: Sequential Changes of NLRP3 Inflammasome Activation in Sepsis and its Relationship With Death. *Shock* 2020, 54(3):294-300.
22. Gonzalez C, Begot E, Dalmay F, Pichon N, François B, Fedou AL, Chapellas C, Galy A, Mancia C, Daix T *et al*: Prognostic impact of left ventricular diastolic function in patients with septic shock. *Ann Intensive Care* 2016, 6(1).
23. Hall TC, Bilku DK, Al-Leswas D, Neal CP, Horst C, Cooke J, Metcalfe MS, Dennison AR: A randomized controlled trial investigating the effects of parenteral fish oil on survival outcomes in critically ill patients with sepsis: a pilot study. *JPEN J Parenter Enteral Nutr* 2015, 39(3):301-312.
24. He M, Huang J, Li X, Liang S, Wang Q, Zhang H: Risk Factors for Mortality in Sepsis Patients without Lactate Levels Increasing Early. *Emerg Med Int* 2023, 2023:6620157.
25. Holmbom M, Andersson M, Grabe M, Peeker R, Saudi A, Styrke J, Aljabery F: Community-onset urosepsis: incidence and risk factors for 30-day mortality - a retrospective cohort study. *Scand J Urol* 2022, 56(5-6):414-420.
26. Hsieh MS, Hu SY, Liao SH, Chang CM, Hsieh VCR, How CK: Type 2 Diabetic Sepsis Patients Have a Lower Mortality Rate in Pioglitazone Use: A Nationwide 15-Year Propensity Score Matching Observational Study in Taiwan. *Emerg Med Int* 2021, 2021.
27. Huang CT, Ruan SY, Tsai YJ, Ku SC, Yu CJ: Clinical Trajectories and Causes of Death in Septic Patients with a Low APACHE II Score. *J Clin Med* 2019, 8(7).
28. Huang AC, Lee TY, Ko MC, Huang CH, Wang TY, Lin TY, Lin SM: Fluid balance correlates with clinical course of multiple organ dysfunction syndrome and mortality in patients with septic shock. *PLoS One* 2019, 14(12):e0225423.
29. Inchai J, Pothirat C, Liwsrisakun C, Deesomchok A, Kositsakulchai W, Chalermpanchai N: Ventilator-associated pneumonia: epidemiology and prognostic indicators of 30-day mortality. *Jpn J Infect Dis* 2015, 68(3):181-186.
30. Jamme M, Daviaud F, Charpentier J, Marin N, Thy M, Hourmant Y, Mira JP, Pène F: Time Course of Septic Shock in Immunocompromised and Nonimmunocompromised Patients. *Crit Care Med* 2017, 45(12):2031-2039.
31. Jouffroy R, Gilbert B, Tourtier JP, Bloch-Laine E, Ecollan P, Boularan J, Bounes V, Vivien B, Gueye P: Prehospital pulse pressure and mortality of septic shock patients cared for by a mobile intensive care unit. *BMC Emerg Med* 2023, 23(1).
32. Kang CSE, Choi S, Jang EJ, Joo S, Jeong JH, Oh SY, Ryu HG, Lee HN: Prevalence and outcomes of chronic comorbid conditions in patients with sepsis in Korea: a nationwide cohort study from 2011 to 2016. *BMC Infect Dis* 2024, 24(1).
33. Kattouf N, Assaf M, Haidar S, Bachir R, El Sayed M, Bouchebl R: The Risk Factors for Mortality among Septic Trauma Patients: A Retrospective Cohort Study Using the National Trauma Data Bank. *Genet Res* 2022, 2022.
34. Khwannimit B, Bhurayanontachai R: The epidemiology of, and risk factors for, mortality from severe sepsis and septic shock in a tertiary-care university hospital setting. *Epidemiol. Infect* 2009, 137(9):1333-1341.
35. Kikuchi K, Kazuma S, Masuda Y: Improvement in ICU Mortality From Sepsis Associated With Recuperation From Septic Multiple-Organ Failure: A Retrospective, Single-Center, Cohort Study. *Cureus* 2024, 16(3).
36. Kim T, Suh GJ, Kwon WY, Kim KS, Jung YS, Shin SM, Jeong H: Lower serum kallistatin level is associated with 28-day mortality in patients with septic shock. *Intensive Care Med Exp* 2018, 6.
37. Kim IY, Kim S, Ye BM, Kim MJ, Kim SR, Lee DW, Lee SB: Procalcitonin decrease predicts survival and recovery from dialysis at 28 days in patients with sepsis-induced acute kidney injury receiving continuous renal replacement therapy. *PLoS ONE* 2022, 17(12 December).
38. Koçak A, Urfalı S: A Retrospective Analysis of the Predictive Role of RDW, MPV, and MPV/PLT Values in 28-Day Mortality of Geriatric Sepsis Patients: Associations with APACHE II and SAPS II Scores. *Medicina (Kaunas)* 2025, 61(8).
39. Korkmaz P, Erarslan S, Toka O: Evaluation of the association between the neutrophil to lymphocyte ratio and mortality in the patients followed up with the diagnosis of sepsis. *J Clin Anal Med* 2017, 8(3):211-215.
40. Kung CT, Hsiao SY, Tsai TC, Su CM, Chang WN, Huang CR, Wang HC, Lin WC, Chang HW, Lin YJ *et al*: Plasma nuclear and mitochondrial DNA levels as predictors of outcome in severe sepsis patients in the emergency room. *J Transl Med* 2012, 10.
41. Kuperman EF, Showalter JW, Lehman EB, Leib AE, Kraschnewski JL: The impact of obesity on sepsis mortality: a retrospective review. *BMC Infect Dis* 2013, 13:377.
42. Le Borgne P, Feuillassier L, Schenck M, Herbrecht JE, Janssen-Langenstein R, Simand C, Gantzer J, Nannini S, Fornecker LM, Alame K *et al*: Comparison of Short- and Long-Term Mortality in Patients with or without Cancer Admitted to the ICU for Septic Shock: A Retrospective Observational Study. *Cancers (Basel)* 2022, 14(13).
43. Lee JS, Choi HS, Ko YG, Yun DH: Performance of the Geriatric Nutritional Risk Index in predicting 28-day hospital mortality in older adult patients with sepsis. *Clin Nutr* 2013, 32(5):843-848.
44. Lee JE, Kang DH, Ju H, Oh DK, Lee SY, Park MH, Lim CM, Lee SI, Korean sepsis Alliance i: Epidemiology and risk factors of fungal pathogens in sepsis: a prospective nationwide multicenter cohort study. *BMC infect dis* 2025, 25(1):331.
45. Li JY, Yao RQ, Liu SQ, Zhang YF, Yao YM, Tian YP: Efficiency of Monocyte/High-Density Lipoprotein Cholesterol Ratio Combined With Neutrophil/Lymphocyte Ratio in Predicting 28-Day Mortality in Patients With Sepsis. *Front Med* 2021, 8.
46. Lin CY, Wang YH, Chen YM, Hung KY, Chang YC, Fang YT, Chang YT, Chen HC, Huang KT, Chang HC *et al*: Dynamic monitoring of kidney injury status over 3 days in the intensive care unit as a sepsis phenotype associated with hospital mortality and hyperinflammation. *Biomed J* 2022, 45(4):665-674.
47. Liu G, Shang Z, Ning N, Li J, Sun W, Fan Y, Guo Y, Ye J, Zhou W, Qian J *et al*: EVALUATION OF PROGNOSTIC RISK MODELS BASED ON AGE AND COMORBIDITY IN SEPTIC PATIENTS: INSIGHTS FROM MACHINE LEARNING AND TRADITIONAL METHODS IN A LARGE-SCALE, MULTICENTER, RETROSPECTIVE STUDY. *Shock* 2025, 64(1):56-64.
48. López R, Pérez-Araos R, Baus F, Moscoso C, Salazar A, Graf J, Montes JM, Samtani S: Outcomes of Sepsis and Septic Shock in Cancer Patients: Focus on Lactate. *Front Med* 2021, 8.
49. López-Mestanza C, Andaluz-Ojeda D, Gómez-López JR, Bermejo-Martín JF: Clinical factors influencing mortality risk in hospital-acquired sepsis. *J Hosp Infect* 2018, 98(2):194-201.
50. Machado Lessa CL, Branchini G, Moreira Delfino I, Ramos Voos MH, Teixeira C, Hoher JA, Nunes FB: Comparison of Sepsis-1, 2 and 3 for Predicting Mortality in Septic Patients of a Middle-Income Country: A Retrospective Observational Cohort Study. *J Intensive Care Med* 2024, 39(4):349-357.
51. MacPhail A, Dendle C, Slavin M, Weinkove R, Bailey M, Pilcher D, McQuilten Z: Sepsis mortality among patients with haematological malignancy admitted to intensive care 2000-2022: a binational cohort study. *Crit Care* 2024, 28(1).
52. Maia MO, da Silveira CDG, Gomes M, Fernandes SES, de Santana RB, de Oliveira DQ, Amorim FFP, Neves FAR, Amorim FF: Multidrug-Resistant Bacteria on Critically Ill Patients with Sepsis at Hospital Admission: Risk Factors and Effects on Hospital Mortality. *Infect Drug Resist* 2023, 16:1693-1704.
53. Man MY, Shum HP, Lam SM, Li J, Yan WW, Yeung MW: Effect of the underlying malignancy on critically ill septic patient's outcome. *Asia-Pac J Clin Oncol* 2022, 18(4):473-480.
54. Matos J, Struja T, Woite NL, Restrepo D, Waschka AK, Celi LA, Sauer CM: A causal inference framework to compare the effectiveness of life-sustaining ICU therapies-using the example of cancer patients with sepsis. *Int J Cancer* 2025.
55. Mazzone A, Dentali F, La Regina M, Foglia E, Gambacorta M, Garagiola E, Bonardi G, Clerici P, Concia E, Colombo F *et al*: Clinical Features, Short-Term Mortality, and Prognostic Risk Factors of Septic Patients Admitted to Internal Medicine Units Results of an Italian Multicenter Prospective Study. *Medicine (Baltimore)* 2016, 95(4).
56. Mușat F, Păduraru DN, Bolocan A, Palcău CA, Bunea AA, Ion D, Andronic O: Sepsis Burden in a Major Romanian Emergency Center-An 18-Year Retrospective Analysis of Mortality and Risk Factors. *Medicina (Kaunas)* 2025, 61(5).
57. Nam H, Jang SH, Hwang YI, Kim JH, Park JY, Park S: Nonpulmonary risk factors of acute respiratory distress syndrome in patients with septic bacteraemia. *Korean J Intern Med* 2019, 34(1):116-124.
58. Nannan Panday RS, Wang S, Schermer EH, Cooksley T, Alam N, Nanayakkara PWB: Septic patients with cancer: Do prehospital antibiotics improve survival? A sub-analysis of the PHANTASi trial. *Neth J Med* 2020, 78(1):3-9.
59. Nielsen FE, Chafranska L, Sørensen RH, Schmidt TA, Abdullah OB: Two-Year Mortality and Prognostic Factors in Sepsis: A Prospective Cohort Study of 714 Danish Emergency Department Patients. *Clin Epidemiol* 2025, 17:581-592.
60. Nygård ST, Langeland N, Flaatten HK, Fanebust R, Haugen O, Skrede S: Aetiology, antimicrobial therapy and outcome of patients with community acquired severe sepsis: a prospective study in a Norwegian university hospital. *BMC* *Infect Dis* 2014, 14.
61. Oliveira RP, Weingartner R, Ribas EO, Moraes RS, Friedman G: Acute haemodynamic effects of a hypertonic saline/dextran solution in stable patients with severe sepsis. *Intensive Care Med* 2002, 28(11):1574-1581.
62. Oliveros H, Tuta-Quintero E, Piñeros M, Guesguan A, Reyes LF: One-year survival of patients admitted for sepsis to intensive care units in Colombia. *BMC* *Infect Dis* 2024, 24(1).
63. Orak M, Karakoç Y, Üstündag M, Yildirim Y, Çelen MK, Güloglu C: An Investigation of the Effects of the Mean Platelet Volume, Platelet Distribution Width, Platelet/Lymphocyte Ratio, and Platelet Counts on Mortality in Patents with Sepsis who applied to the Emergency Department. *Niger J Clin Pract* 2018, 21(5):667-671.
64. Orsetti E, Brescini L, Mazzanti S, Trave F, Morroni G, Pocognoli A, Berardi R, Barchiesi F: Central venous catheter unrelated candidemia influences the outcome of infection in patients with solid tumors. *Eur J Clin Microbiol Infect Dis* 2019, 38(8):1499-1505.
65. Pansiritanachot W, Ruangsomboon O, Limsuwat C, Chakorn T: Independent risk factors of mortality in patients with sepsis receiving single-dose etomidate as an induction agent during rapid sequence intubation in a large tertiary emergency department in Thailand. *BMC Emerg Med* 2022, 22(1).
66. Park DW, Chunx BC, Kim JM, Sohn JW, Peck KR, Kim YS, Choi YH, Choi JY, Kim SI, Eom JS *et al*: Epidemiological and clinical characteristics of community-acquired severe sepsis and septic shock: A prospective observational study in 12 university hospitals in Korea. *J Korean Med Sci* 2012, 27(11):1308-1314.
67. Paulsen J, Mehl A, Askim Å, Solligård E, Åsvold BO, Damås JK: Epidemiology and outcome of Staphylococcus aureus bloodstream infection and sepsis in a Norwegian county 1996-2011: an observational study. *BMC Infect Dis* 2015, 15:116.
68. Pittet D, Thiévent B, Wenzel RP, Li N, Gurman G, Suter PM: Importance of pre-existing co-morbidities for prognosis of septicemia in critically ill patients. *Intensive Care Med* 1993, 19(5):265-272.
69. Proaño-Zamudio JA, Argandykov D, Gebran A, Renne A, Paranjape CN, Maroney SJ, Onyewadume L, Kaafarani HMA, King DR, Velmahos GC *et al*: Open Abdomen in Elderly Patients With Surgical Sepsis: Predictors of Mortality. *J Surg Res* 2023, 287:160-167.
70. Qu ZY, Zhu YB, Wang MP, Li W, Zhu B, Jiang L, Xi XM: Prognosis and Risk Factors of Sepsis Patients in Chinese ICUs: A Retrospective Analysis of a Cohort Database. *Shock* 2021, 56(6):921-926.
71. Rannikko J, Syrjänen J, Seiskari T, Aittoniemi J, Huttunen R: Sepsis-related mortality in 497 cases with blood culture-positive sepsis in an emergency department. *Int J Infec Dis* 2017, 58:52-57.
72. Ravetti CG, Moura AD, Vieira TL, Pedroso ERP, Teixeira AL: STREM-1 predicts intensive care unit and 28-day mortality in cancer patients with severe sepsis and septic shock. *J Crit Care* 2015, 30(2):440.e447-440.e413.
73. Reaven MS, Rozario NL, McCarter MSJ, Heffner AC: Incidence and risk factors associated with early death in patients with emergency department septic shock. *Acute Crit Care* 2022, 37(2):193-201.
74. Riché FC, Dray X, Laisné MJ, Matéo J, Raskine L, Sanson-Le Pors MJ, Payen D, Valleur P, Cholley BP: Factors associated with septic shock and mortality in generalized peritonitis: Comparison between community-acquired and postoperative peritonitis. *Crit Care* 2009, 13(3).
75. Ripa M, Rodríguez-Núñez O, Cardozo C, Naharro-Abellán A, Almela M, Marco F, Morata L, De La Calle C, Del Rio A, Garcia-Vidal C *et al*: Influence of empirical double-active combination antimicrobial therapy compared with active monotherapy on mortality in patients with septic shock: A propensity score-adjusted and matched analysis. *J Antimicrob Chemother* 2017, 72(12):3443-3452.
76. Roh J, Jo EJ, Eom JS, Mok J, Kim MH, Kim KU, Park HK, Lee MK, Yeom S, Lee K: Factors predicting long-term survival of patients with sepsis on arrival at the emergency department: A single-center, observational study. *Medicine (Baltimore)* 2019, 98(33).
77. Rotter J, Dowlati E, Cobourn K, Kalhorn C: A cross-sectional analysis of variables associated with morbidity and mortality in postoperative neurosurgical patients diagnosed with sepsis. *Acta Neurochir* 2020, 162(11):2837-2848.
78. Rygård SL, Holst LB, Wetterslev J, Johansson PI, Perner A: Higher vs. lower haemoglobin threshold for transfusion in septic shock: subgroup analyses of the TRISS trial. *Acta Anaesthesiol Scand* 2017, 61(2):166-175.
79. Ryoo SM, Kim WY, Sohn CH, Seo DW, Koh JW, Oh BJ, Lim KS: Prognostic value of timing of antibiotic administration in patients with septic shock treated with early quantitative resuscitation. *Am J Med Sci* 2015, 349(4):328-333.
80. Sakr Y, Reinhart K, Vincent JL, Sprung CL, Moreno R, Ranieri VM, De Backer D, Payen D: Does dopamine administration in shock influence outcome? Results of the Sepsis Occurrence in Acutely Ill Patients (SOAP) Study. *Crit Care Med* 2006, 34(3):589-597.
81. Sakr Y, Maia VPL, Santos C, Stracke J, Zeidan M, Bayer O, Reinhart K: Adjuvant selenium supplementation in the form of sodium selenite in postoperative critically ill patients with severe sepsis. *Crit Care* 2014, 18(2).
82. Sartelli M, Abu-Zidan FM, Labricciosa FM, Kluger Y, Coccolini F, Ansaloni L, Leppäniemi A, Kirkpatrick AW, Tolonen M, Tranà C *et al*: Physiological parameters for Prognosis in Abdominal Sepsis (PIPAS) Study: a WSES observational study. *World J Emerg Surg* 2019, 14:34.
83. Schramm GE, Kashyap R, Mullon JJ, Gajic O, Afessa B: Septic shock: A multidisciplinary response team and weekly feedback to clinicians improve the process of care and mortality. *Critical Care Medicine* 2011, 39(2):252-258.
84. Schumer W: Steroids in the treatment of clinical septic shock. *Ann Surg* 1976, 184(3):333-341.
85. Sharma A, Nguyen P, Taha M, Soubani AO: Sepsis Hospitalizations with Versus without Cancer: Epidemiology, Outcomes, and Trends in Nationwide Analysis from 2008 to 2017. *Am J Clin Oncol* 2021, 44(10):505-511.
86. Shi W, Xie M, Mao E, Yang Z, Zhang Q, Chen E, Chen Y: Development and validation of a prediction model for in-hospital mortality in patients with sepsis. *Nurs Criti Care* 2025, 30(3):e70015.
87. Su CM, Kung CT, Chen FC, Cheng HH, Hsiao SY, Lai YR, Huang CC, Tsai NW, Lu CH: Manifestations and outcomes of patients with Parkinson's disease and serious infection in the emergency department. *Biomed Res Int* 2018, 2018.
88. Sundén-Cullberg J, Norrby-Teglund A, Rouhiainen A, Rauvala H, Herman G, Tracey KJ, Lee ML, Andersson J, Tokics L, Treutiger CJ: Persistent elevation of high mobility group box-1 protein (HMGB1) in patients with severe sepsis and septic shock. *Crit Care Med* 2005, 33(3):564-573.
89. Tai HY, Zhu ZY, Mei HF, Sun WB, Zhang W: Albumin-to-Fibrinogen Ratio Independently Predicts 28-Day Mortality in Patients with Peritonitis-Induced Sepsis. *Mediators Inflamm* 2020, 2020.
90. Thibaud P, Chow-Chine L, Gonzalez F, Bisbal M, Servan L, Sannini A, Tezier M, Tourret M, Cambon S, Pouliquen C *et al*: Septic shock and biliary sepsis: 90-day mortality and associated risk factors. *HPB* 2024, 26(2):270-281.
91. Tolsma V, Schwebel C, Azoulay E, Darmon M, Souweine B, Vesin A, Goldgran-Toledano D, Lugosi M, Jamali S, Cheval C *et al*: Sepsis severe or septic shock: outcome according to immune status and immunodeficiency profile. *Chest* 2014, 146(5):1205-1213.
92. Wang JT, McDonald LC, Chang SC, Ho M: Community-acquired Acinetobacter baumannii bacteremia in adult patients in Taiwan. *J Clin Microbiol* 2002, 40(4):1526-1529.
93. Wang HJ, Zhang PJ, Chen WJ, Feng D, Jia YH, Xie LX: Serum MicroRNA Signatures Identified by Solexa Sequencing Predict Sepsis Patients' Mortality: A Prospective Observational Study. *PLOS ONE* 2012, 7(6).
94. Wang HE, Szychowski JM, Griffin R, Safford MM, Shapiro NI, Howard G: Long-term mortality after community-acquired sepsis: a longitudinal population-based cohort study. *BMJ Open* 2014, 4(1):e004283.
95. Wang MP, Jiang L, Zhu B, Li W, Du B, Kang Y, Weng L, Qin TH, Ma XC, Zhu DM *et al*: The Prevalence, Risk Factors, and Outcomes of Sepsis in Critically Ill Patients in China: A Multicenter Prospective Cohort Study. *Front Med* 2020, 7.
96. Wang AY, Chang CK: Do -not -attempt resuscitation independently predict in -hospital mortality in septic patients. *Am J Emerg Med* 2020, 38(5):953-957.
97. Wang B, Chen J: Establishment and validation of a predictive model for mortality within 30 days in patients with sepsis-induced blood pressure drop: A retrospective analysis. *PLoS ONE* 2021, 16(5 May).
98. Wang H, Ji X, Wang AY, Wu PK, Liu Z, Dong L, Liu J, Duan M: Epidemiology of sepsis-associated acute kidney injury in Beijing, China: A descriptive analysis. *Int J Gen Med* 2021, 14:5631-5649.
99. Wu HP, Shih CC, Lin CY, Hua CC, Chuang DY: Serial increase of IL-12 response and human leukocyte antigen-DR expression in severe sepsis survivors. *Crit Care* 2011, 15(5):R224.
100. Xie J, Wang H, Kang Y, Zhou L, Liu Z, Qin B, Ma X, Cao X, Chen D, Lu W *et al*: The Epidemiology of Sepsis in Chinese ICUs: A National Cross-Sectional Survey. *Crit Care Med* 2020, 48(3):e209-e218.
101. Yin J, Chen Y, Huang JL, Yan L, Kuang ZS, Xue MM, Sun S, Xiang H, Hu YY, Dong ZM *et al*: Prognosis-related classification and dynamic monitoring of immune status in patients with sepsis: A prospective observational study. *World J Emerg Med* 2021, 12(3):185-191.
102. Zhang H, Wang Y, Zhang X, Xu C, Xu D, Shen H, Jin H, Yang J, Zhang X: Carbapenem-resistant Enterobacterales sepsis following endoscopic retrograde cholangiopancreatography: risk factors for 30-day all-cause mortality and the development of a nomogram based on a retrospective cohort. *Antimicrob Resist Infect Control* 2024, 13(1).
103. Zhang J, Xu P, Huang Y, Li J, Ma C, Liu G, Ye J, Guo Y, Feng Z, Jiang B *et al*: Diagnostic Value of RDW-Albumin Ratio for the Prediction of Mortality in Sepsis Associated Nonthyroidal Illness Syndrome Patients: A Retrospective Cohort Study. *J Inflamm Res* 2024, 17:11305-11318.
104. Zhou J, Qian C, Zhao M, Yu X, Kang Y, Ma X, Ai Y, Xu Y, Liu D, An Y *et al*: Epidemiology and outcome of severe sepsis and septic shock in intensive care units in Mainland China. *PLoS ONE* 2014, 9(9).
105. Zhou X, Zeng N, Liu P, Liu Z, Duan M: Sex Differences in In-hospital Mortality of Patients With Septic Shock: An Observational Study Based on Data Analysis From a Cover Sheet of Medical Records in Beijing. *Front Med* 2021, 8.
106. Wan X, Wang W, Liu J, Tong T: Estimating the sample mean and standard deviation from the sample size, median, range and/or interquartile range. *BMC Med Res Methodol* 2014, 14:135.
107. Garnacho-Montero J, Aldabo-Pallas T, Garnacho-Montero C, Cayuela A, Jiménez R, Barroso S, Ortiz-Leyba C: Timing of adequate antibiotic therapy is a greater determinant of outcome than are TNF and IL-10 polymorphisms in patients with sepsis. *Crit Care* 2006, 10(4):R111.
108. Jouffroy R, Gilbert B, Tourtier JP, Bloch-Laine E, Ecollan P, Bounes V, Boularan J, Gueye-Ngalgou P, Vivien B: The prehospital SIGARC score to assess septic shock in-hospital, 30-day and 90-day mortality. *Am J Emerg Med* 2021, 46:355-360.
109. Lai H, Wu G, Zhong Y, Chen G, Zhang W, Shi S, Xia Z: Red blood cell distribution width improves the prediction of 28-day mortality for patients with sepsis-induced acute kidney injury: A retrospective analysis from MIMIC-IV database using propensity score matching. *J Intensive Med* 2023, 3(3):275-282.
110. Li H, Zu Y, Wang Q, Zi T, Qin X, Zhao Y, Jiang W, Wang X, Xu C, Chen X *et al*: Risk factor analysis and nomogram development for predicting 28-day mortality in elderly ICU patients with sepsis and type 2 diabetes mellitus. *Eur J Inflamm* 2024, 22.
111. Li C, Zhao K, Ren Q, Chen L, Zhang Y, Wang G, Xie K: Development and validation of a model for predicting in-hospital mortality in patients with sepsis-associated kidney injury receiving renal replacement therapy: a retrospective cohort study based on the MIMIC-IV database. *Front Cell Infect Microbiol* 2024, 14.
112. Liu Y, Sun R, Jiang H, Liang G, Huang Z, Qi L, Lu J: Development and validation of a predictive model for in-hospital mortality in patients with sepsis-associated liver injury. *Ann Transl Med* 2022, 10(18).
113. Ma C, Zhang L, Wang M, Zhang F, Zhang L: Prophylactic proton pump inhibitor use and all-cause mortality in adult sepsis patients: a retrospective analysis based on the MIMIC-IV database. *Front Pharmaco* 2025, 16.
114. Mu S, Yan D, Tang J, Zheng Z: Predicting Mortality in Sepsis-Associated Acute Respiratory Distress Syndrome: A Machine Learning Approach Using the MIMIC-III Database. *J Intensive Care Med* 2025, 40(3):294-302.
115. Peng M, Deng F, Qi D, Hu Z, Zhang L: The Hyperbilirubinemia and Potential Predictors Influence on Long-Term Outcomes in Sepsis: A Population-Based Propensity Score-Matched Study. *Front Med* 2021, 8.
116. Peng L, Peng C, Yang F, Wang J, Zuo W, Cheng C, Mao Z, Jin Z, Li W: Machine learning approach for the prediction of 30-day mortality in patients with sepsis-associated encephalopathy. *BMC Med Res Methodol* 2022, 22(1):183.
117. Weng J, Hou RN, Zhou XM, Xu Z, Zhou ZL, Wang P, Wang L, Chen C, Wu JY, Wang ZY: Development and validation of a score to predict mortality in ICU patients with sepsis: a multicenter retrospective study. *J Transl Med* 2021, 19(1).
118. Zhang L, Zhang H, Huang Z, Ye S, Sun X, Lin R, Li S, Lan J, Yang D: Relationship between time-weighted average anion gap and mortality in septic shock patients: a retrospective analysis of the MIMIC-IV database. *BMC* *Infect Dis* 2025, 25(1).
